# Tracking Development Assistance for Health: A Comparative Study of the 29 Development Assistance Committee Countries, 2011–2019

**DOI:** 10.3390/ijerph18168519

**Published:** 2021-08-12

**Authors:** Shuhei Nomura, Haruka Sakamoto, Aya Ishizuka, Kenji Shibuya

**Affiliations:** 1 Department of Health Policy and Management, School of Medicine, Keio University, 35 Shinanomachi, Shinjuku-ku, Tokyo 160-8582, Japan; harukask@m.u-tokyo.ac.jp (H.S.); aishizuka@m.u-tokyo.ac.jp (A.I.); 2 Department of Global Health Policy, Graduate School of Medicine, The University of Tokyo, 7-3-1 Hongo, Bunkyo-ku, Tokyo 113-0033, Japan; kenjishibuya1@gmail.com; 3 Soma COVID Vaccination Medical Center, 63-3 Nakamura Kitamachi, Fukushima, Soma City 976-8601, Japan

**Keywords:** official development assistance, development assistance for health, Development Assistance Committee

## Abstract

Development assistance for health (DAH) is an important part of financing healthcare in low- and middle-income countries. We estimated the gross disbursement of DAH of the 29 Development Assistance Committee (DAC) member countries of the Organisation for Economic Co-operation and Development (OECD) for 2011–2019; and clarified its flows, including aid type, channel, target region, and target health focus area. Data from the OECD iLibrary were used. The DAH definition was based on the OECD sector classification. For core funding to non-health-specific multilateral agencies, we estimated DAH and its flows based on the OECD methodology for calculating imputed multilateral official development assistance (ODA). The total amount of DAH for all countries combined was 18.5 billion USD in 2019, at 17.4 USD per capita, with the 2011–2019 average of 19.7 billion USD. The average share of DAH in ODA for the 29 countries was about 7.9% in 2019. Between 2011 and 2019, most DAC countries allocated approximately 60% of their DAH to primary health care, with the remaining 40% allocated to health system strengthening. We expect that the estimates of this study will help DAC member countries strategize future DAH wisely, efficiently, and effectively while ensuring transparency.

## 1. Introduction

The novel coronavirus disease (COVID-19) pandemic has demonstrated the importance of a resilient health system and universal health coverage (UHC) in ensuring equitable access to health, especially at times of emergency. However, vulnerable populations across the globe, such as women, children, the elderly, refugees, and the poor, faced increased difficulty accessing basic health services, thereby offsetting the potential to achieve the Sustainable Development Goals by 2030 [1].

Traditionally, donor countries have utilized Development Assistance for Health (DAH) for promoting key health agendas, such as UHC. Working off of a global commitment to UHC made at the 2019 United Nations General Assembly High Level Meeting on UHC [2], heads of states, finance and health ministers of 20 countries and other world leaders at the 2019 Group of 20 (G20) Summit and Ministerial Meetings in Osaka, Japan [3], declared a shared understanding of the importance of UHC financing in developing countries, along with noting the importance of sustainable financing for health [4]. This shared understanding encourages further investment in primary health care (PHC) services by promoting UHC through the preferential use of fair and equitable domestic funding, while also suggesting that the DAH should be strategically mobilized in areas that cannot be addressed by recipient countries themselves and should be harmonized with the health financing and development needs of recipient countries.

Today, DAH must adapt to the complex conditions surrounding health issues, such as the growing health impact of climate change, the complications of conflict and migration, and global political trends that emphasize national interests [5,6]. In addition, DAH needs to be tailored to consider the demographic and epidemiologic transitions that many low-income countries are undergoing, where people are living longer and enduring a greater variety of diseases. 

In addition to supporting global health improvements, DAH is an important component of foreign policy for many donors [7,8]. By improving health and promoting economic development, DAH can enhance and support security, trade, and the development and use of global public goods [9]. In an increasingly interconnected world, outbreaks of emerging diseases, antimicrobial resistance (AMR), and other health threats can easily spread across the globe, and efforts to prevent or limit these threats in one country often benefit neighbouring and distant countries [10]. DAH can deliver benefits to donors and recipients alike and provide a path to shared global prosperity [11]. In particular, the COVID-19 outbreak has highlighted the importance of reconsidering the appropriateness of existing support and diplomatic strategies for countries with diverse population structures, varying public health needs, limited health resources, and fragile economies [12,13].

Organisation for Economic Co-operation and Development (OECD) member countries are often high-income countries and provide large amounts of DAH. The objective of this paper is to present an overview of the DAH implemented by the member countries of OECD’s Development Assistance Committee (DAC) by estimating the gross disbursement of DAH of the 29 DAC member countries for 2011–2019 and clarifying its flows, including aid type, channel, target region, and target health focus area. The results of this study will contribute to re-examining strategic decision-making and the effective implementation of DAH by the DAC countries.

## 2. Materials and Methods

### 2.1. Data

Data on ODA projects from 2011 to 2019, implemented by the governments of all 29 DAC countries, were considered and downloaded from the OECD iLibrary [14]. These data contain the gross disbursements of ODA, aid type, target country or region, and target health focus area for each project and year. Aid type includes bilateral grants, such as technical assistance, bilateral loan, earmarked funding (bi-multi) to multilateral agencies, and core funding to multilateral agencies (i.e., non-earmarked funding and assessed contributions). A country’s eligibility to receive ODA is based on its GNI per capita as calculated by the World Bank. The DAC list of ODA recipients, which is updated every three years, shows all countries eligible to receive ODA [15]. The health focus area was based on purpose codes for sector classification defined by OECD [16], known as Creditor Reporting System [CRS] codes. According to OECD, aid activities are grouped into broad three-digit sector categories, each of which is further classified into five-digit purpose codes. In the present study, we defined DAH as ODA in 120 (Health) and 130 (Population policies/Programs and reproductive health) sector categories based on the previous studies, including the 23 five-digit purpose codes [17,18,19,20,21]. Until 2019, there were no purpose codes that specifically addressed noncommunicable diseases (NCDs) and no systematic means to ensure accountability for donors’ commitments for NCDs. Starting with the 2019 reporting system for the 2018 ODA flows, however, new six purpose codes were adopted exclusively for NCDs and some of their risk factors for donors to report on the ODA flow for NCDs [22]. Until the 2017 ODA flows, projects related to NCDs were reported as part of medical research and services [22].

The gross domestic product (GDP) deflator from the OECD national accounts was employed to convert current prices for 2011–2018 to constant prices at 2019 [23]. For the estimation of per-capita DAH, we used the population data from the Population Division within the Department of Economic and Social Affairs of the United Nations [24]. For Hungary, Poland, and the Slovak Republic, data for 2011–2013, 2011–2012, and 2011–2012 were not included in the OECD iLibrary, partly because their membership in the DAC has begun after 2011. 

### 2.2. Imputed Multilateral Aid to Health

With regard to core funding to multilateral agencies that are not specialized in the health sector (e.g., the World Bank), it was not possible to directly identify DAH, its target country/region, and health focus area among ODA. We, therefore, estimated the DAH based on the OECD methodology for calculating imputed multilateral ODA as follows [25]. First, we calculated the ODA flows to the health sector of each agency (i.e., DAH) as a proportion of total ODA disbursements (α: health sector share of the total ODA of the agency), in accordance with reports from multilateral agencies to the OECD [14]. Similarly, each agency’s DAH flows to each target country/region and each health focus area were also calculated (β: share of target country/region of the agency’s DAH, and γ: share of the health focus area of the agency’s DAH). Then, the total ODA of each DAC member country was multiplied by α and β or γ to estimate flows of countries’ DAH through the agency. For instance, the multilateral DAH from Japan through the World Bank was estimated by multiplying Japan’s total ODA to the World Bank by α. Additionally, Japan’s DAH through the World Bank was estimated as total ODA × α × β and total ODA × α × γ for specific target country/region and health focus area, respectively. This methodology was validated in our previous work [26,27] and elsewhere [28].

### 2.3. Primary Health Care and Health System Strengthening

In the spirit of Alma Ata and Astana, a well-functioning PHC system is considered to be the cornerstone of a country that is successfully financing high-quality health services for the entire population, which is essential to achieve UHC [29,30]. While there is no standardized measurement of DAH for PHC on the current CRS system, Shaw et al. (2015) and our previous work used CRS purpose code data to define DAH on “PHC delivery” and on “health system strengthening (HSS)” in support of PHC delivery. The present study also followed this definition and methodology and estimated how much 29 countries’ DAH was invested in PHC and HSS [26,31]. Our working definition of PHC focused only on inputs under the control of the health system itself, not on the intersectoral interventions (e.g., safe water, sanitation, and hygiene). Therefore, the scope of PHC included treatment of diseases and injuries, infectious disease control, reproductive health, and public health measures targeting prevention. DAH for such scope was referred to as being most relevant to “PHC delivery” in this study.

On the other hand, proper functioning PHC requires system-wide investments, including an effective prioritization mechanism, sound management, sufficient human and institutional capacities to carry out operational, financial, and technical tasks, updated health information systems for policy and program monitoring and evaluation, and appropriate regulatory, governance, financial, and accountability mechanisms. As in previous studies, this study referred to DAH for such investment as being most relevant to “HSS” in support of PHC delivery [26,31]. Therefore, our working definition of HSS is narrower than the extensive discussion of HSS that is common in the literature, where most public expenditures for improving health care can be interpreted as HSS. Detailed definitions of PHC and HSS and their validity have been discussed in previous studies [26,31]. A list of CRS purpose codes corresponding to PHC and HSS is provided in the table of results of this study.

## 3. Results

The estimated DAH for the 29 DAC member countries and all countries combined in constant prices at 2019 from 2011 to 2019 as well as those per capita is shown in Table 1. The total amount of DAH for all countries combined was 18.5 billion USD in 2019, at 17.4 USD per capita; the 2011–2019 average was found to be 19.7 billion USD with a standard deviation of 1.2 billion USD. The highest amount in 2019 was 8.0 billion USD for the United States (accounting for about 43.4% of the total in the 29 countries), followed by 2.9 USD billion for the United Kingdom, 1.4 billion USD for Germany, 1.0 billion USD for Japan, and 0.9 billion USD for France. On the other hand, in terms of per capita value, the highest value was found in Luxembourg at 109.1 USD, followed by Norway at 92.6 USD, Sweden at 45.9 USD, the United Kingdom at 42.2 USD, and the Netherlands at 30.6 USD. 

**Figure 1:**
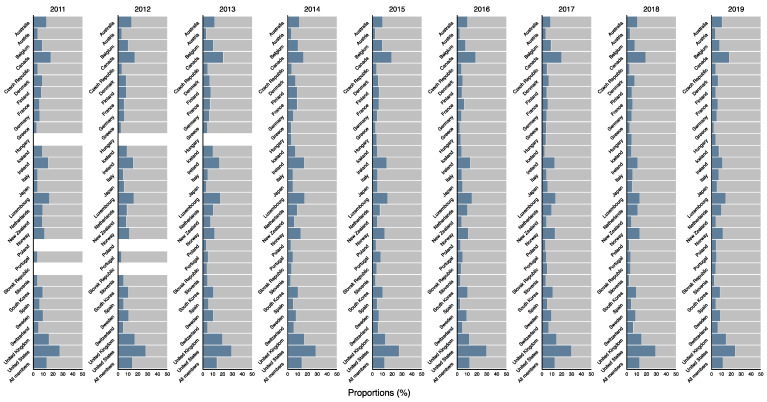
Proportion of estimated DAH in ODA for all the 29 DAC member countries, 2011–2019. Blue: DAH; Gray: ODA for non-health areas; DAH: development assistance for health; DAC: Development Assistance Committee.

In terms of the proportion of DAH in total ODA by country, the largest proportion in 2019 was 23.9% in the United States, followed by 17.8% in Canada, 14.6% in the United Kingdom, 14.3% in Luxembourg, and 11.6% in Norway. The average proportion among the 29 countries was about 7.9%. Among the total ODA in the 29 countries, the proportion of DAH was 11.5% in 2019, and there has been no remarkable change since 2011. The distribution of the proportions in the 29 countries has also not changed much since 2011 (Figure 1). Detailed data can be found in Table S1.

**Figure 2:**
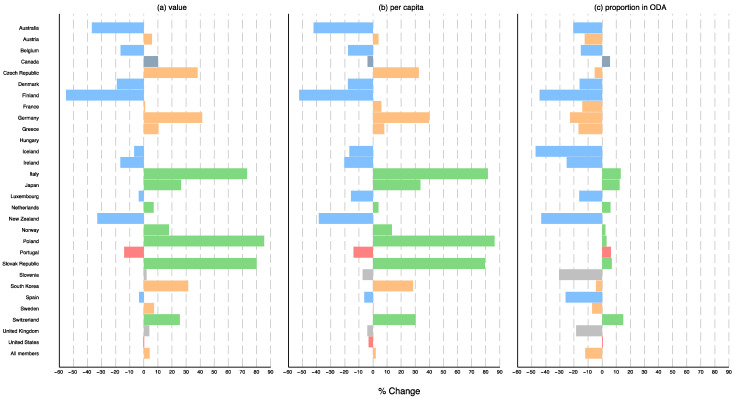
Percentage change in the average of estimated DAH for 2016–2019 from 2011–2014 for all the 29 DAC member countries: (**a**) value, (**b**) per capita and (**c**) proportion of DAH in ODA. Blue: all decreased; Red: (**a**,**b**) decreased and (**c**) increased; Gray: (**a**) increased and (**b**,**c**) decreased; Orange: (**a**,**b**) increased and (**c**) decreased; Navy: (**a**,**c**) increased and (**b**) decreased; Green: all increased; DAH: development assistance for health; DAC: Development Assistance Committee; ODA: official development assistance.

Figure 2 shows the percentage change in the averages of estimated total DAH, per capita DAH, and DAH as a proportion of ODA between 2011–2014 and 2016–2019 for the 28 countries (excluding Hungary, which had no data for the first three years) and all countries combined. Nine out of the 28 countries (Australia, Belgium, Denmark, Finland, Iceland, Ireland, Luxembourg, New Zealand, and Spain) showed an adverse change in all. On the other hand, seven countries (Italy, Japan, Netherlands, Norway, Poland, Slovak Republic, and Switzerland) showed an upward trend for all. Austria, Czech Republic, France, Germany, Greece, South Korea, and Sweden had an increase in the total DAH and DAH per capita but a decrease in the DAH percentage. Detailed data can be found in Table S2.

**Figure 3:**
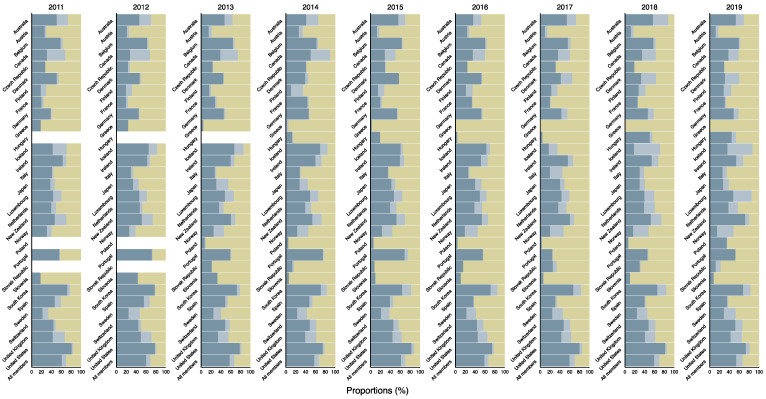
Estimated DAH from all the 29 DAC member countries by development type, 2011–2019. Blue: bilateral aid (excluding bi-multi); Light blue: bi-multi aid; Yellow: multilateral aid; DAH: development assistance for health; DAC: Development Assistance Committee.

Figure 3 shows the 2011–2019 trends in DAH from the 29 countries and all countries combined by aid type. Among total DAH in the 29 countries, about 30% of the aid was multilateral aid, and 70% was bilateral aid between 2011 and 2019. Bi-multi aid, which is part of bilateral aid, accounted for about 10 percent of the total. The distribution of aid types varied by country. In Greece and Slovenia, multilateral aid accounted for nearly 90 percent of DAH since 2014. Meanwhile, more than 70% of DAH from the United States and South Korea was bilateral aid in the study period. Detailed data can be found in Table S3.

**Figure 4:**
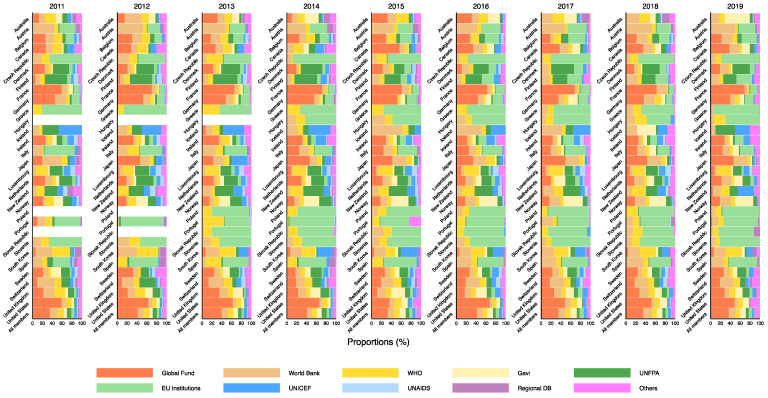
Estimated DAH from all the 29 DAC member countries, channelled through multilateral agencies, 2011–2019. DAH: development assistance for health; DAC: Development Assistance Committee; Global Fund: The Global Fund to Fight AIDS, Tuberculosis and Malaria; WHO: World Health Organization; Gavi: Gavi, The Vaccine Alliance; UNFPA: United Nations Population Fund; EU Institutions: Institutions of the European Union; UNICEF: United Nations Children’s Fund; UNAIDS: Joint United Nations Programme on HIV/AIDS; Regional DB: regional development banks.

Figure 4 shows the 2011–2019 trends in DAH channelled through multilateral agencies from the 29 countries and all countries combined. In 2011–2019, overall, the largest amount of DAH (around 30–40% of the total) was channelled through the Global Fund to Fight AIDS, Tuberculosis and Malaria (Global Fund), followed by the World Bank, Gavi, the Vaccine Alliance (Gavi), and World Health Organization (WHO) with approximately 10% each. The United States was the largest donor to the WHO, accounting for 20–30% of total DAC contributions in recent years. The trends varied widely from country to country, with the Group of Seven (G7) roughly contributing considerably to the Global Fund, while most other European countries contributed substantially to the institutions of the European Union. Detailed data can be found in Table S4.

Trends in DAH from 2011 to 2019 by region for the 29 countries and all countries combined illustrated that Africa was the main target region for most countries, followed by Asia, while Australia and New Zealand also made a relatively large contribution to the Oceania region (Figure S1). Among total DAH in the 29 countries, about 50% and 20% were allocated to Africa and Asia over 2011–2019. Detailed data can be found in Table S5.

Trends in DAH from 2011–2019 by health focus area for the 29 countries and all countries combined showed that sexually transmitted diseases (STD) control, including HIV/AIDS, was the most common (25.8% in 2019) overall, followed by basic healthcare (11.8%), malaria control (11.0%), and infectious disease control (excluding malaria, tuberculosis, and STD) (9.6%) (Figure S2). There was a large variation among countries, but the shares of basic health care and health policy and administrative management were relatively high in many countries. The share of NCDs area in 2018 was less than 1% (average 0.6%) in all countries except for New Zealand (12.5%), and the average in 2019 was 1.6%. Detailed data can be found in Table S6.

As reported in Figure S3 with exact values presented in Table S7, out of the 29 countries, 23 countries allocate more than 60% of DAH to PHC, with the remaining 40% allocated to HSS, across the study periods.

## 4. Discussion

This study provides comparable estimates of DAH disbursements and their flows from 29 OECD’s DAC member countries based on the OECD CRS purpose code structure. The total DAH of 29 countries in 2019 was found to be approximately 18.5 billion USD. The United States was the largest donor, accounting for about 43.4% of the total in 2019. The average share of DAH in ODA for the 29 countries was about 7.9%. The share varied widely among countries, with the largest being the United States at 23.9%. This study evaluated changes in DAH since 2011 in terms of value, per capita, and health sector share and found that nine countries showed a downward trend in all indicators. On the other hand, seven countries showed an upward trend in all indicators.

In this study, we investigated the distribution of earmarked funding (bi-multi) and core funding to multilateral agencies for DAH from 2011 to 2019. Bi-multi funding refers to the provision of earmarked funds to multilateral agencies, where donors have some control over decisions regarding the disposal of funds. These flows may cover specific countries, projects, regions, sectors, or themes. This type of assistance for bilateral functions through multilateral agencies is considered by the OECD and other organizations to be a part of bilateral ODA [32]. Core funding for multilateral agencies, on the other hand, is flexible funding used for a variety of purposes, including global functions (providing global public goods, managing cross-border externalities, and fostering stewardship and leadership). Recent literature demonstrated that the total share of core funding in 2013 for global functions for health was estimated to be 62% for WHO, 40% for UNAIDS, 22% for UNFPA, 20% for Gavi; 12% for UNICEF, 10% for the Global Fund, and 5% for the World Bank [21]. Experience from multiple recent outbreaks, including the 2009 H1N1 influenza pandemic and the later 2014–15 Ebola outbreak, provided strong indications that the world needs sustainable financing for global functions [33]. Many of the tasks involved in controlling highly contagious infectious diseases such as COVID-19 are global public goods, those that flow across national borders and have far-reaching consequences and can be delivered through global cooperation. An example of the kind of cooperation needed is the research and development of drugs, vaccines, diagnostics, and other health tools; they must be manufactured and distributed at scale in a globally fair and equitable fashion [21,33].

In all 29 DAC countries, core funding accounted for the majority of DAH through multilateral agencies. In particular, the United States held the largest share, accounting for 20–30% of the total contributions of the 29 DAC countries to WHO. In April 2020, the United States announced that it would suspend its contributions to the WHO as a poignant criticism of WHO’s response to COVID-19 [34]. According to the WHO, the United States’ contribution accounts for about 15% of the overall WHO budget in 2018 and 2019 [35]. Specifically, the United States’ contribution accounted for 15% of the WHO’s budget for polio eradication; 40% of tuberculosis control, 31% of HIV/AIDS, 15% of malaria control, 13% of vaccine-preventable diseases, and 26% of emergency health operations programme budgets [35]. In January 2021, the new President of the United States, Joe Biden, announced that the United States would resume funding the WHO [36]. This example implies that a sector that relies heavily on contributions from certain countries can easily risk its financial sustainability for political, economic, and social reasons.

As for the trend where about half of the DAH was allocated to Africa, contributions by the former colonial powers, alongside the United States, Japan, and other emerging donors, can be noted. In particular, Japan and China have strategically stepped up their support to Africa in recent years, intending to strengthen political and economic relations in the region. For example, in 2016, Japan hosted Tokyo International Conference on African Development (TICAD) VI in Kenya and launched the “UHC in Africa: Framework for Action” in partnership with the World Bank, the Global Fund, WHO, and the African Development Bank [37]. This represented a roadmap for African countries to accelerate progress toward UHC and to monitor and assess their progress. Furthermore, at TICAD VII held in Yokohama, Japan, in August 2019, the Yokohama Declaration 2019 was adopted to promote a strong and sustainable society for human security in Africa, including the achievement of the Sustainable Development Goals (SDGs) and the African Union Agenda 2063 as well as UHC [38]. China has increased its support to the continent of Africa through their Forum on China-Africa Cooperation (FOCAC) held every three years since 2000 and has committed to strengthening the surveillance and response to emerging diseases in the continent and strengthening the health system overall [39]. China has spearheaded the global response to expand COVID-19 vaccination in Africa and has committed to providing the vaccine to more than 40 African countries as of June 2021 [40].

It was also shown that between 2011 and 2019, most DAC countries allocated approximately 60% of their DAH to PHC, with the remaining 40% allocated to HSS. The effective distribution of DAH has long been debated, but it does not necessarily coincide with disease burdens and the cost-effectiveness of interventions in recipient countries [41]. The DAH allocation can be guided by many factors, including diplomatic relations, geographical proximity, and trade-related considerations, particularly in bilateral aid. However, in the era of the SDGs, the DAH has become even more important for strengthening health systems [42]. Today, many recipient countries are experiencing a rising burden of morbidity, mortality, and associated health costs from NCDs in addition to the burden of high levels of communicable diseases [43]. Although PHCs have played a successful role in interventions to prevent and care for infectious diseases such as malaria, tuberculosis, and HIV/AIDS, countries in transition need to expand their existing PHC delivery for health promotion, disease prevention, and treatment in response to NCDs [44]. Several studies suggest that responding to NCDs requires a diagonal approach to HSS rather than vertical disease-specific programmes [45,46]. The HSS has even become an important focus of multilateral agencies such as the World Bank, the Global Fund, and Gavi.

There is a growing debate about why donors should invest more in NCDs [47]. This does not mean that funds for infectious disease control should be reduced in order to allocate and scale up for NCD measures through HSS. Instead, the allocation of DAHs should take full account of health transition in DAH recipient countries and make disease burden an important criterion for prioritizing resource allocation [6]. Dieleman et al. (2019) has shown that DAH per disability-adjusted life-year (a measure of total health burden) also varies markedly across countries [6]. Meanwhile, the DAH share to the NCDs area in this study was only 1–2% in 2019. Given that the NCD-specific purpose codes have only just been adopted, it might be possible that DAC countries are not yet properly reporting their ODA flows according to the objective codes’ descriptions.

While the ODA system is well known, its use has many complexities. Gross disbursements (the amount actually distributed) was considered in the study, rather than commitments (the amount the donor agreed to make available). Disbursements are more volatile than commitments and may depend on events occurring in a particular country (such as political or economic instability) and the absorptive capacity of recipient countries [48].

As noted in previous studies [26,31], it is difficult to draw a clear conclusion about the PHC and HSS shares for several reasons. First, there is no global consensus on measurable indications for PHC and HSS. There is also no normative description of the donor’s share of DAH in PHC and HSS. The methods developed in previous studies were reproducible using OECD/CRS data and may be useful for tracking donor resources allocated to PHC/HSS in the future. However, caution is necessary for understanding that our working definition of PHC adopted from these studies is very broad, and there may be some overlap with HSS.

## 5. Conclusions

The issue of global health has received attention at the highest levels in recent years, but it has also been recognized as a national security issue in the wake of recent human security challenges and the recurrence of epidemics. Nevertheless, still, DAH growth has stagnated over the past ten years globally, and limited funding is a universal constraint. Today, there is a need to expand international cooperation through ODA to combat a pandemic never seen in scale. In particular, support for global public goods (including the development of new drugs and vaccines for COVID-19) and long-term guidance for developing countries to reduce their socio-economic impacts of COVID-19 are needed. This paper, which evaluated the DAH trend before COVID-19, is expected to contribute to re-examining strategic decision-making and the effective implementation of future DAH by DAC countries while promoting transparency of DAH flows.

## Figures and Tables

**Table 1:** Estimated DAH in million USD at a constant price of 2019 (per capita) from all the 29 DAC member countries, 2011–2019.

Country	2011	2012	2013	2014	2015	2016	2017	2018	2019
Australia	480.6 (21.3)	558.8 (24.4)	461.8 (19.9)	440.6 (18.7)	367.7 (15.4)	345.0 (14.2)	249.0 (10.1)	325.4 (13.1)	306.3 (12.0)
Austria	46.7 (5.5)	39.1 (4.6)	38.6 (4.5)	42.2 (4.9)	38.6 (4.4)	44.7 (5.1)	47.9 (5.4)	38.0 (4.3)	45.6 (5.1)
Belgium	245.6 (22.3)	227.7 (20.5)	225.0 (20.2)	241.1 (21.5)	210.1 (18.6)	203.6 (17.9)	211.6 (18.5)	185.2 (16.1)	184.5 (15.9)
Canada	812.9 (23.5)	805.9 (23.1)	867.8 (24.6)	608.6 (17.1)	865.2 (24.0)	785.7 (21.6)	878.8 (23.9)	897.1 (24.2)	844.3 (22.4)
Czech Republic	10.0 (0.9)	8.3 (0.8)	9.8 (0.9)	8.5 (0.8)	9.8 (0.9)	12.7 (1.2)	16.8 (1.6)	8.3 (0.8)	12.8 (1.2)
Denmark	236.4 (42.3)	210.3 (37.5)	187.4 (33.2)	220.4 (38.9)	161.7 (28.4)	145.8 (25.5)	176.5 (30.8)	199.9 (34.8)	169.8 (29.3)
Finland	106.2 (19.7)	106.2 (19.6)	105.7 (19.4)	141.1 (25.8)	92.8 (16.9)	52.7 (9.6)	56.1 (10.2)	49.6 (9.0)	47.1 (8.5)
France	760.6 (12.0)	755.4 (11.9)	843.1 (13.2)	1074.3 (16.7)	745.2 (11.6)	849.5 (13.1)	824.1 (12.7)	875.6 (13.5)	913.0 (14.0)
Germany	917.2 (11.3)	882.9 (10.9)	942.7 (11.6)	988.5 (12.1)	1003.1 (12.3)	1175.2 (14.3)	1321.3 (16.0)	1364.6 (16.4)	1415.2 (16.9)
Greece	11.2 (1.0)	7.8 (0.7)	9.1 (0.8)	7.5 (0.7)	8.2 (0.8)	9.9 (0.9)	12.9 (1.2)	8.3 (0.8)	8.2 (0.8)
Hungary	NA	NA	NA	5.4 (0.6)	7.3 (0.7)	7.5 (0.8)	6.2 (0.6)	13.7 (1.4)	12.9 (1.3)
Iceland	2.8 (8.7)	2.9 (8.9)	4.3 (13.1)	3.2 (9.7)	2.3 (7.0)	2.7 (8.1)	1.7 (5.1)	3.2 (9.5)	4.7 (13.8)
Ireland	125.8 (27.4)	120.6 (26.2)	130.7 (28.3)	128.4 (27.8)	107.3 (23.1)	108.4 (23.1)	108.6 (22.8)	97.8 (20.3)	106.5 (21.6)
Italy	171.0 (2.9)	131.5 (2.2)	158.9 (2.6)	195.5 (3.2)	218.0 (3.6)	239.2 (3.9)	275.7 (4.5)	296.1 (4.9)	327.2 (5.4)
Japan	696.8 (5.4)	855.7 (6.7)	717.1 (5.6)	855.5 (6.7)	866.1 (6.8)	888.8 (7.0)	1096.2 (8.6)	948.5 (7.5)	1021.1 (8.1)
Luxembourg	63.0 (121.3)	62.9 (118.5)	71.3 (131.4)	67.1 (121.0)	60.4 (106.6)	63.4 (109.4)	61.5 (103.9)	61.6 (101.9)	68.3 (109.1)
Netherlands	556.3 (33.2)	488.0 (29.1)	534.9 (31.8)	504.3 (29.9)	488.9 (28.9)	569.2 (33.5)	524.1 (30.8)	611.4 (35.8)	523.6 (30.6)
New Zealand	36.6 (8.3)	35.4 (7.9)	32.6 (7.2)	28.7 (6.3)	22.2 (4.8)	19.7 (4.2)	20.4 (4.3)	25.1 (5.3)	24.1 (5.0)
Norway	379.1 (76.6)	391.3 (78.0)	474.9 (93.5)	506.3 (98.5)	522.2 (100.4)	508.5 (96.8)	538.6 (101.7)	514.9 (96.5)	502.1 (92.6)
Poland	NA	NA	15.2 (0.4)	13.9 (0.4)	17.8 (0.5)	24.4 (0.6)	24.6 (0.6)	18.5 (0.5)	35.6 (0.9)
Portugal	26.8 (2.5)	19.2 (1.8)	25.6 (2.4)	23.4 (2.2)	32.1 (3.1)	22.8 (2.2)	19.3 (1.9)	16.7 (1.6)	23.0 (2.3)
Slovak Republic	NA	NA	3.1 (0.6)	2.9 (0.5)	3.9 (0.7)	4.4 (0.8)	6.6 (1.2)	5.0 (0.9)	4.9 (0.9)
Slovenia	2.3 (1.1)	3.2 (1.6)	2.6 (1.3)	1.7 (0.8)	2.1 (1.0)	2.7 (1.3)	3.0 (1.4)	1.9 (0.9)	2.4 (1.2)
South Korea	139.5 (2.8)	180.7 (3.6)	200.5 (4.0)	197.4 (3.9)	209.6 (4.1)	246.6 (4.8)	242.4 (4.7)	228.6 (4.5)	226.9 (4.4)
Spain	235.8 (5.0)	106.7 (2.3)	125.6 (2.7)	104.4 (2.2)	88.5 (1.9)	141.6 (3.0)	158.4 (3.4)	125.1 (2.7)	128.1 (2.7)
Sweden	438.6 (46.3)	466.4 (48.9)	478.8 (49.8)	432.5 (44.6)	459.1 (47.0)	464.6 (47.2)	509.2 (51.4)	510.3 (51.2)	463.4 (45.9)
Switzerland	142.5 (18.0)	144.5 (18.0)	147.7 (18.2)	205.3 (25.0)	198.6 (23.9)	185.0 (22.1)	214.1 (25.3)	203.1 (23.8)	201.4 (23.3)
United Kingdom	2056.3 (32.1)	2183.1 (33.8)	3275.8 (50.4)	2819.9 (43.1)	2224.0 (33.8)	2194.4 (33.1)	2807.3 (42.1)	2874.3 (42.8)	2862.6 (42.2)
United States	9906.8 (31.8)	9890.0 (31.5)	10,225.3 (32.3)	10,463.2 (32.8)	9167.2 (28.6)	11,106.6 (34.4)	11,035.2 (33.9)	10,261.6 (31.4)	8049.7 (24.3)
All members	18,607.3 (18.1)	18,684.7 (18.1)	20,315.6 (19.6)	20,331.8 (19.5)	18,200.0 (17.4)	20,425.3 (19.4)	21,447.9 (20.3)	20,769.4 (19.6)	18,535.2 (17.4)

DAH: development assistance for health; DAH: Development Assistance Committee.

## Data Availability

No new data were created or analyzed in this study. Data sharing is not applicable to this article.

## Supplementary Materials

The following are available online at https://www.mdpi.com/article/10.3390/ijerph18168519/s1, Figure S1: Estimated DAH from all the 29 DAC member countries by target region, 2011–2019, Figure S2: Estimated DAH from all the 29 DAC member countries by health focus area, 2011–2019, Figure S3: Estimated DAH from all the 29 DAC member countries for PHC delivery and HSS in support of PHC delivery, 2011–2019, Table S1: Proportion of estimated DAH in ODA from all the 29 DAC member countries, 2011–2019, Table S2: Percentage change between the average of estimated DAH of for 2011–2014 and 2016–2019 for all the 29 DAC member countries: (a) total DAH, (b) DAH per capita, (c) proportion (%) of DAH in ODA, Table S3: Estimated DAH in constant prices at 2019 from all the 29 DAC member countries by development type (%), 2011–2019, Table S4: Estimated DAH in constant prices at 2019 from all the 29 DAC member countries, channeled through multilateral agencies (%), 2011–2019, Table S5: Estimated DAH in constant prices at 2019 from all the 29 DAC member countries by target region (%), 2011–2019, Table S6: Estimated DAH in constant prices at 2019 from all the 29 DAC member countries by health focus area (%), 2011–2019, Table S7: Estimated DAH in constant prices at 2019 from all the 29 DAC member countries for PHC delivery and HSS in support of PHC delivery (%), 2011–2019.
