# Supplementary material for: Tracking Development Assistance for Health: A Comparative Study of the 29 Development Assistance Committee Countries, 2011–2019
Source: Int J Environ Res Public Health. 2021 Aug 12;18(16):8519. doi: 10.3390/ijerph18168519 (PMC8394202; doi:10.3390/ijerph18168519)
Supplement: Supplementary file 1 [file ijerph-18-08519-s001.zip › ijerph-1319468-supplementary.pdf]

Figure S1: Estimated DAH from all the 29 DAC member countries by target region, 2011–2019

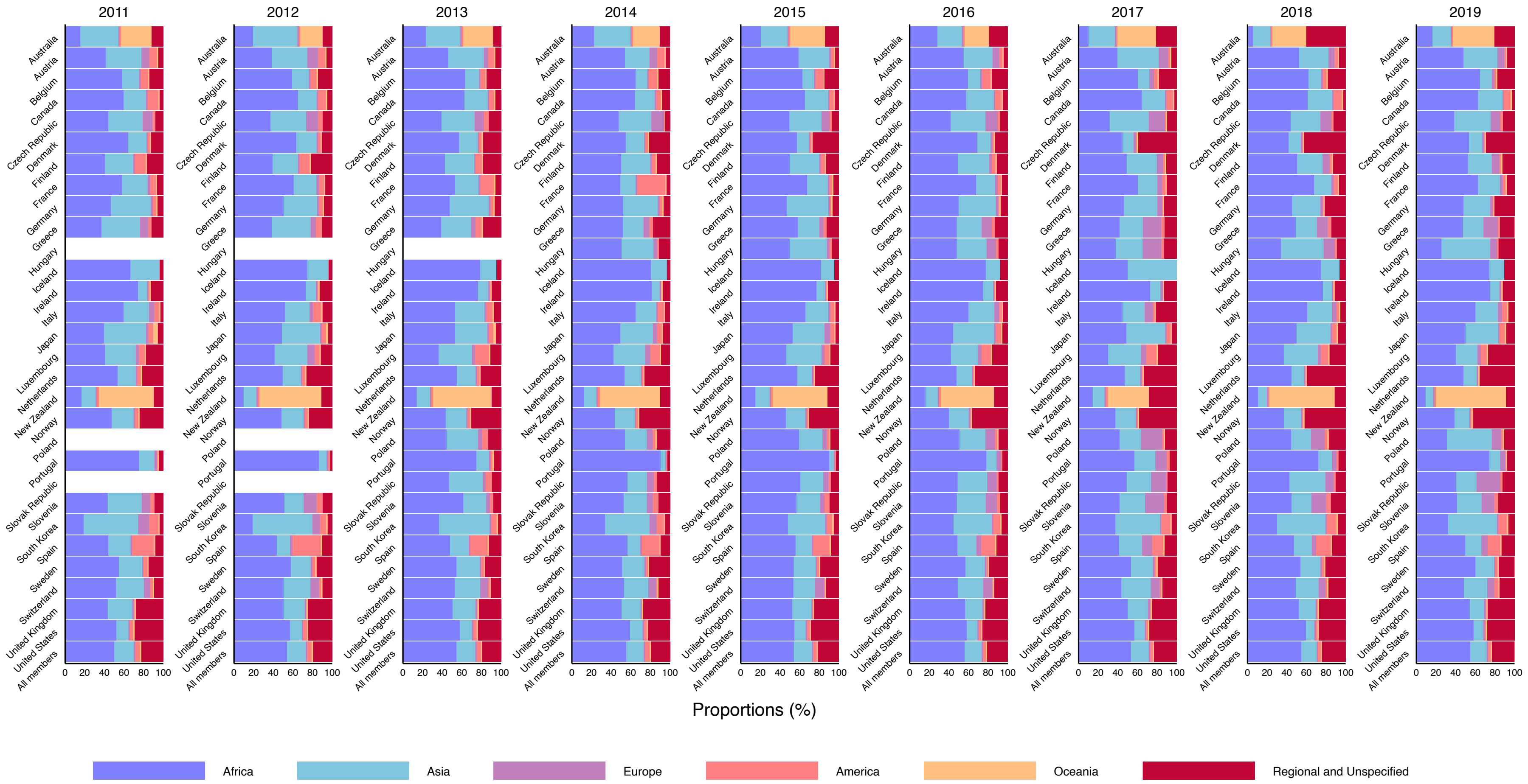

DAH: development assistance for health; DAC: Development Assistance.

Figure S2: Estimated DAH from all the 29 DAC member countries by health focus area, 2011–2019

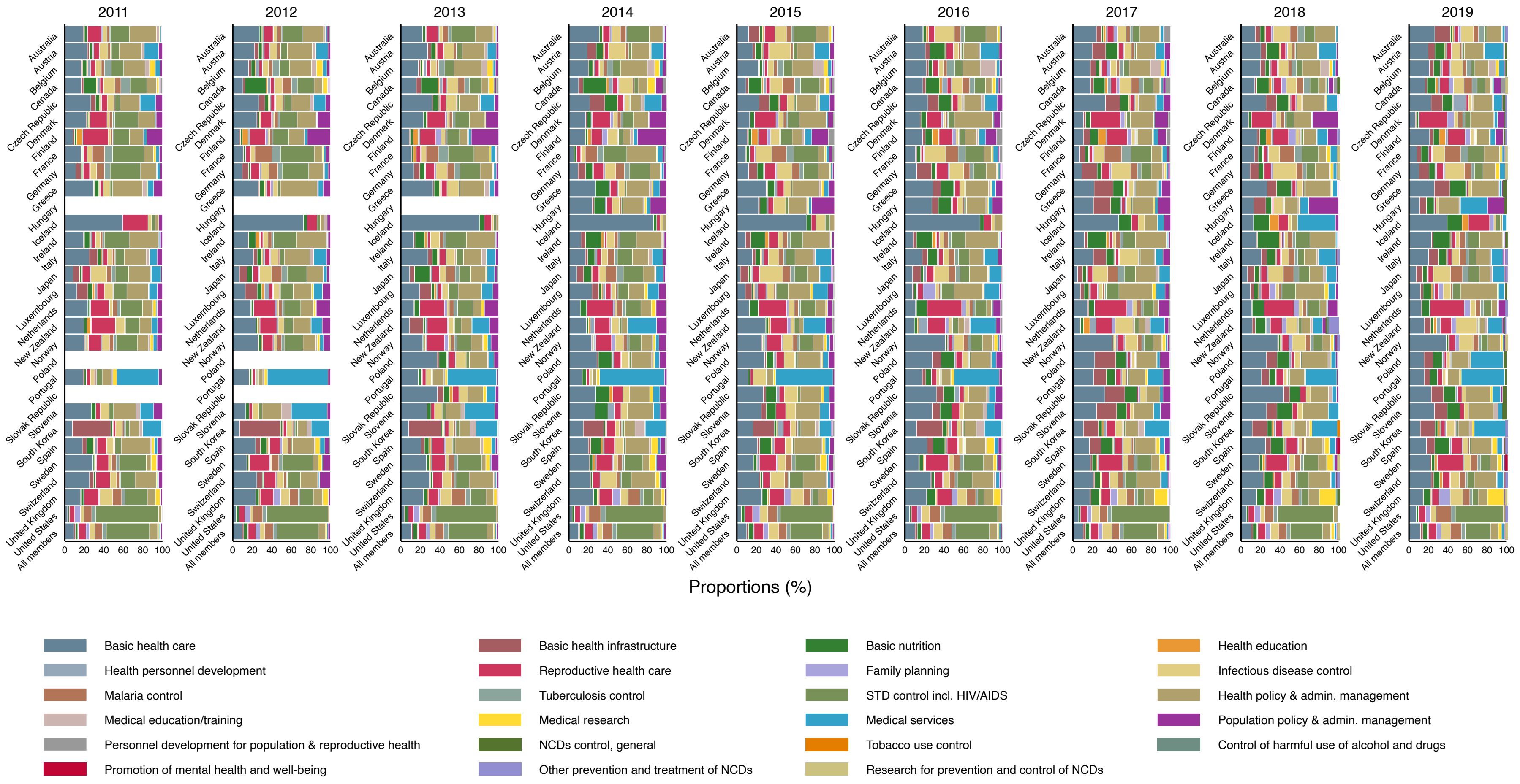

DAH: development assistance for health; DAC: Development Assistance Committee; STD: sexually transmitted disease. CRS purpose code: Basic health care = 12220; Basic health infrastructure = 12230; Basic nutrition = 12240; Health education = 12261; Health personnel development = 12281; Reproductive health care = 13020; Family planning = 13030; Infectious disease control = 12250; Malaria control = 12262; Tuberculosis control = 12263; STD control including HIV/AIDS = 13040; Health policy and administrative management = 12110; Medical education/training = 12181; Medical research=12182; Medical services = 12191; Population policy and administrative management = 13010; Personnel development for population and reproductive health = 13081; NCDs control, general = 12310; Tobacco use control = 12320; Control of harmful use of alcohol and drugs = 12330; Promotion of mental health and well-being = 12340; Other prevention and treatment of NCDs = 12350; Research for prevention and control of NCDs = 12382.

Figure S3: Estimated DAH from all the 29 DAC member countries for PHC delivery and HSS in support of PHC delivery, 2011–2019

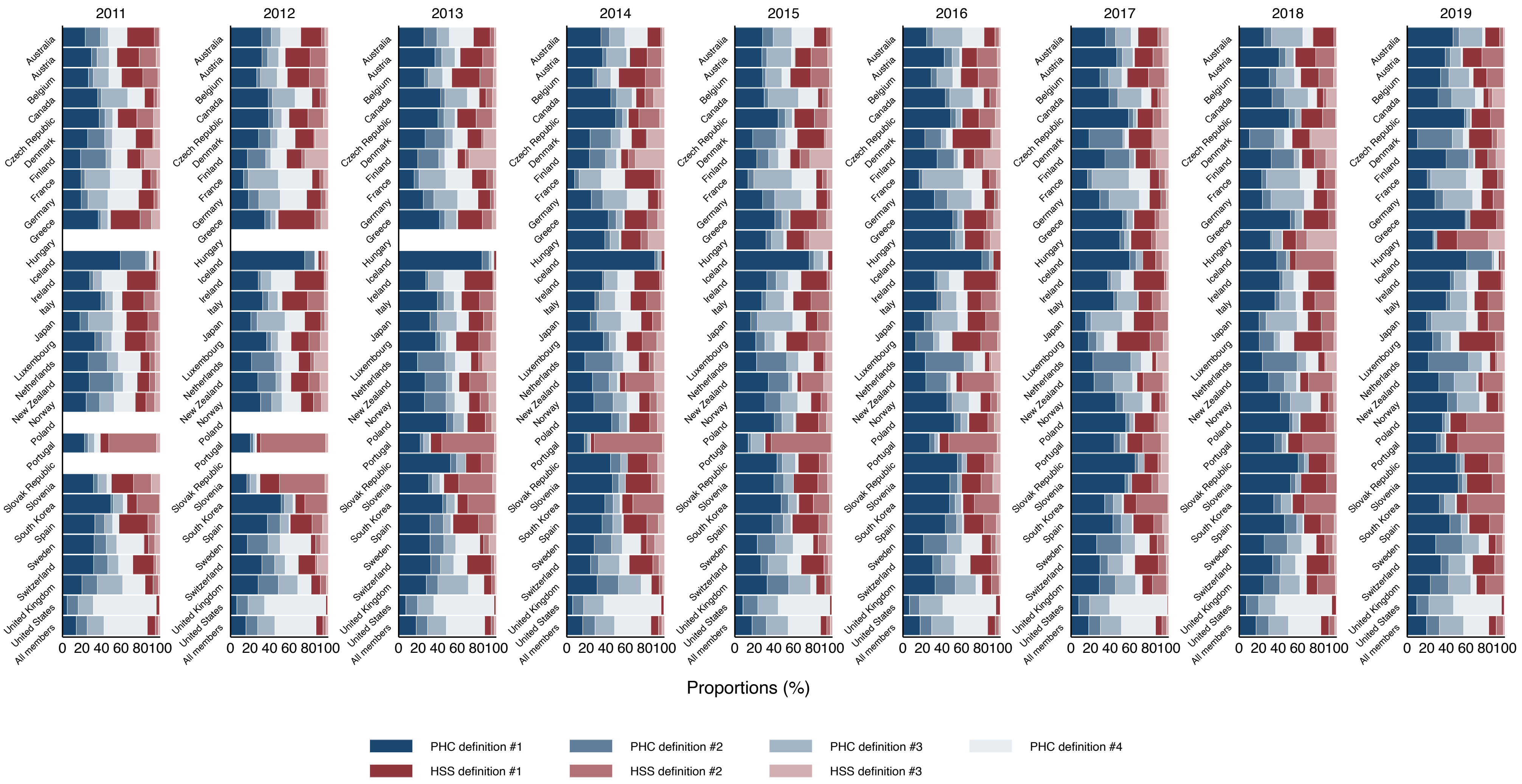

DAH: development assistance for health; DAH: Development Assistance Committee; PHC: public health care; HSS: health system strengthening. PHC definition #1 = Basic health care and infrastructure (CRS purpose codes: 12220, 12230, 12240, 12261, 12281); PHC definition #2 = Reproductive health care and family planning (13020, 13030); PHC definition #3 = Infectious disease control, including malaria and tuberculosis (12250, 12262, 12263); PHC definition #4 = Sexually transmitted disease (STD) control including

HIV/AIDS (13040); Broader PHC definition = Definition #1 + #2 + #3 + #4; HSS definition #1 = Health policy, administration & management (12110); HSS definition #2 = Medical services, training & research (12181, 12182, 12191, 12310, 12320, 12330, 12340, 12350, 12382); HSS definition #3 = Population policy & administration (13010, 13081); Broader HSS definition = Definition #1 + #2 + #3.

Table S1: Proportion of estimated DAH in ODA from all the 29 DAC member countries, 2011–2019

Country	2011	2012	2013	2014	2015	2016	2017	2018	2019
Australia	13.3	13.6	11.8	11.8	10.3	10.3	8.5	10.7	10.6
Austria	4.5	3.6	3.5	3.7	2.7	2.6	3.7	3.4	3.7
Belgium	8.8	10.0	10.4	10.6	9.9	8.2	9.2	8.1	8.3
Canada	17.8	17.0	20.7	16.0	19.4	18.6	19.9	19.2	17.8
Czech Republic	4.5	3.9	4.8	4.0	4.2	4.3	5.1	2.8	4.1
Denmark	9.0	8.1	6.8	8.0	5.8	5.5	6.9	7.9	6.5
Finland	8.0	8.2	8.0	9.6	6.7	4.7	4.9	5.1	4.1
France	6.1	6.0	7.4	9.7	6.5	7.0	5.9	5.9	6.3
Germany	6.4	6.2	6.3	5.6	4.8	4.1	4.6	4.9	5.3
Greece	3.4	2.8	4.6	3.5	3.4	2.6	4.1	3.0	2.2
Hungary	NA	NA	NA	4.0	4.3	3.4	4.1	4.9	4.1
Iceland	9.2	9.0	10.2	7.8	4.9	4.3	2.6	4.6	7.6
Ireland	15.0	15.3	16.6	16.9	14.4	13.0	12.8	11.0	11.0
Italy	4.3	5.0	5.1	5.4	5.2	4.5	4.5	6.0	7.4
Japan	4.6	6.0	3.4	5.4	5.2	5.3	5.7	5.4	5.4
Luxembourg	16.3	15.9	17.6	17.2	15.3	14.9	13.8	13.1	14.3
Netherlands	9.5	9.1	10.5	9.7	7.8	10.3	9.9	11.0	9.9
New Zealand	9.0	8.3	7.9	6.5	4.9	4.3	4.7	4.6	4.4
Norway	11.3	11.3	11.9	13.2	12.3	11.1	13.1	13.0	11.6
Poland	NA	NA	3.4	3.3	3.7	3.3	3.4	2.4	4.5
Portugal	4.0	3.2	5.3	5.4	8.5	5.5	4.4	3.9	5.2
Slovak Republic	NA	NA	4.1	4.0	4.3	3.9	5.3	3.7	4.2
Slovenia	4.0	5.6	4.7	3.0	3.1	3.1	3.8	2.4	2.7
South Korea	9.7	10.4	10.8	10.5	10.4	10.5	10.9	9.6	8.6
Spain	6.2	5.5	5.5	5.6	4.7	3.0	5.2	4.4	4.3
Sweden	9.8	10.7	10.4	8.5	6.6	9.5	9.5	8.8	8.8
Switzerland	5.2	5.0	5.0	6.2	5.7	5.1	6.7	6.5	6.3
United Kingdom	15.9	16.8	19.8	16.8	13.2	12.0	14.9	15.0	14.6
United States	26.8	28.0	29.0	28.5	26.9	29.8	29.9	29.2	23.9
All members	13.6	14.3	14.3	14.4	12.3	12.5	13.0	12.8	11.5

DAH: development assistance for health; DAH: Development Assistance Committee

Table S2: Percentage change between the average of estimated DAH of for 2011–2014 and 2016–2019 for all the 29 DAC member countries: (a) total DAH, (b) DAH per capita, (c) proportion (%) of DAH in ODA

Country	(a) total DAH	(b) DAH per capita	(c) % of DAH in ODA
Australia	-36.9	-42.1	-20.6
Austria	5.8	3.8	-12.4
Belgium	-16.4	-17.6	-15.1
Canada	10.0	-3.9	5.6
Czech Republic	38.3	32.6	-5.2
Denmark	-19.0	-17.7	-16.0
Finland	-55.2	-52.4	-44.4
France	0.8	6.0	-14.0
Germany	41.4	39.8	-22.9
Greece	10.4	8.0	-16.8
Hungary	NA	NA	NA
Iceland	-6.8	-16.8	-47.2
Ireland	-16.7	-20.3	-25.1
Italy	73.3	81.5	13.1
Japan	26.5	33.6	12.4
Luxembourg	-3.6	-15.6	-16.3
Netherlands	6.9	3.9	5.9
New Zealand	-33.0	-38.4	-43.2
Norway	17.8	13.5	2.3
Poland	85.4	86.3	3.0
Portugal	-13.9	-13.8	6.1
Slovak Republic	80.2	79.5	6.9
Slovenia	2.0	-7.3	-30.6
South Korea	31.5	28.5	-4.3
Spain	-3.4	-6.2	-25.9
Sweden	7.2	0.5	-7.1
Switzerland	25.6	30.3	15.0
United Kingdom	3.9	-4.0	-18.5
United States	-0.1	-3.1	0.4
All members	4.2	1.9	-12.0

DAH: development assistance for health; DAH: Development Assistance Committee; ODA: official development assistance

Table S3: Estimated DAH in constant prices at 2019 from all the 29 DAC member countries by development type (%), 2011–2019

Country	Type	2011	2012	2013	2014	2015	2016	2017	2018	2019
Australia	Bi	245.2 (51.0)	258.8 (46.3)	220.2 (47.7)	182.2 (41.3)	207.5 (56.4)	125.3 (36.3)	134.7 (54.1)	185.9 (57.1)	163.1 (53.2)
	Bi-multi	108.7 (22.6)	134.9 (24.2)	73.7 (16.0)	106.3 (24.1)	50.8 (13.8)	56.2 (16.3)	47.1 (18.9)	100.4 (30.8)	49.7 (16.2)
	Multi	126.7 (26.4)	165.1 (29.5)	167.9 (36.4)	152.2 (34.5)	109.4 (29.8)	163.5 (47.4)	67.2 (27.0)	39.1 (12.0)	93.5 (30.5)
Austria	Bi	12.5 (26.7)	8.4 (21.5)	5.9 (15.4)	11.2 (26.4)	5.1 (13.3)	10.8 (24.1)	4.8 (10.1)	4.7 (12.4)	5.5 (12.0)
	Bi-multi	1.4 (3.1)	1.7 (4.4)	2.4 (6.3)	3.4 (8.0)	1.8 (4.7)	1.8 (4.0)	2.2 (4.5)	1.9 (5.0)	2.7 (6.0)
	Multi	32.8 (70.2)	29.0 (74.2)	30.2 (78.3)	27.7 (65.5)	31.7 (82.0)	32.1 (71.9)	40.9 (85.4)	31.4 (82.6)	37.4 (82.0)
Belgium	Bi	144.3 (58.8)	142.3 (62.5)	145.4 (64.6)	150.1 (62.3)	132.4 (63.0)	123.6 (60.7)	119.1 (56.3)	106.9 (57.7)	109.1 (59.1)
	Bi-multi	9.9 (4.0)	5.3 (2.3)	7.0 (3.1)	8.1 (3.4)	4.7 (2.2)	6.1 (3.0)	12.1 (5.7)	8.7 (4.7)	5.3 (2.9)
	Multi	91.4 (37.2)	80.0 (35.2)	72.5 (32.2)	82.9 (34.4)	73.1 (34.8)	73.9 (36.3)	80.3 (38.0)	69.6 (37.6)	70.1 (38.0)
Canada	Bi	253.5 (31.2)	222.0 (27.5)	340.1 (39.2)	307.5 (50.5)	249.2 (28.8)	278.2 (35.4)	320.2 (36.4)	311.7 (34.7)	321.9 (38.1)
	Bi-multi	303.1 (37.3)	328.6 (40.8)	307.2 (35.4)	239.2 (39.3)	191.2 (22.1)	195.4 (24.9)	211.8 (24.1)	253.2 (28.2)	172.6 (20.4)
	Multi	256.3 (31.5)	255.4 (31.7)	220.5 (25.4)	61.8 (10.2)	424.8 (49.1)	312.1 (39.7)	346.8 (39.5)	332.1 (37.0)	349.8 (41.4)
Czech Republic	Bi	2.8 (28.1)	2.0 (23.5)	2.3 (23.6)	3.5 (40.9)	2.9 (30.0)	3.1 (24.7)	5.3 (31.4)	1.6 (19.9)	3.8 (29.5)
	Bi-multi	0.0 (0.3)	0.3 (3.4)	0.1 (1.0)	0.1 (0.9)	0.1 (0.6)	0.0 (0.3)	0.0 (0.2)	0.0 (0.0)	0.1 (0.7)
	Multi	7.2 (71.6)	6.1 (73.2)	7.4 (75.4)	4.9 (58.2)	6.8 (69.4)	9.5 (75.0)	11.5 (68.4)	6.7 (80.1)	8.9 (69.8)
Denmark	Bi	120.7 (51.1)	99.5 (47.3)	84.1 (44.9)	86.3 (39.2)	93.1 (57.5)	76.6 (52.5)	73.8 (41.8)	66.1 (33.1)	52.9 (31.2)
	Bi-multi	8.8 (3.7)	5.6 (2.7)	2.8 (1.5)	14.5 (6.6)	0.0 (0.0)	2.7 (1.8)	41.1 (23.3)	60.7 (30.4)	49.3 (29.0)
	Multi	106.8 (45.2)	105.2 (50.0)	100.4 (53.6)	119.6 (54.3)	68.6 (42.5)	66.5 (45.7)	61.6 (34.9)	73.1 (36.6)	67.6 (39.8)
Finland	Bi	19.9 (18.7)	20.9 (19.7)	17.5 (16.6)	15.2 (10.8)	14.1 (15.2)	11.3 (21.4)	12.9 (23.0)	13.7 (27.7)	13.4 (28.6)
	Bi-multi	11.1 (10.4)	12.8 (12.0)	3.7 (3.5)	33.9 (24.0)	11.7 (12.6)	7.2 (13.8)	6.7 (12.0)	6.8 (13.8)	7.3 (15.4)
	Multi	75.3 (70.9)	72.5 (68.3)	84.5 (79.9)	92.0 (65.2)	67.1 (72.2)	34.1 (64.8)	36.5 (65.1)	29.0 (58.5)	26.4 (56.0)
France	Bi	152.6 (20.1)	148.8 (19.7)	238.6 (28.3)	468.3 (43.6)	147.3 (19.8)	286.4 (33.7)	174.4 (21.2)	223.0 (25.5)	281.0 (30.8)
	Bi-multi	31.3 (4.1)	29.0 (3.8)	29.0 (3.4)	29.7 (2.8)	27.6 (3.7)	11.9 (1.4)	0.5 (0.1)	11.1 (1.3)	26.7 (2.9)
	Multi	576.6 (75.8)	577.5 (76.5)	575.5 (68.3)	576.2 (53.6)	570.2 (76.5)	551.3 (64.9)	649.2 (78.8)	641.6 (73.3)	605.2 (66.3)
Germany	Bi	359.9 (39.2)	405.7 (46.0)	434.9 (46.1)	460.4 (46.6)	538.3 (53.7)	618.6 (52.6)	568.9 (43.1)	636.0 (46.6)	690.7 (48.8)
	Bi-multi	10.9 (1.2)	8.3 (0.9)	28.3 (3.0)	10.1 (1.0)	13.6 (1.4)	27.1 (2.3)	162.0 (12.3)	163.7 (12.0)	141.1 (10.0)
	Multi	546.4 (59.6)	468.9 (53.1)	479.5 (50.9)	518.0 (52.4)	451.2 (45.0)	529.4 (45.1)	590.4 (44.7)	565.0 (41.4)	583.5 (41.2)
Greece	Bi	2.1 (18.7)	1.9 (23.9)	0.4 (4.0)	0.1 (1.8)	0.2 (2.6)	0.1 (1.0)	0.1 (0.5)	0.0 (0.2)	0.0 (0.0)
	Bi-multi	0.0 (0.0)	0.0 (0.0)	0.0 (0.0)	0.3 (3.5)	0.0 (0.0)	0.0 (0.0)	0.0 (0.2)	0.0 (0.0)	0.0 (0.0)
	Multi	9.1 (81.3)	6.0 (76.1)	8.7 (96.0)	7.1 (94.7)	8.0 (97.4)	9.9 (99.0)	12.8 (99.3)	8.3 (99.8)	8.2 (100.0)

Hungary	Bi	NA	NA	NA	0.7 (12.2)	1.4 (19.0)	0.3 (4.3)	0.3 (5.2)	6.9 (50.3)	5.8 (45.1)
	Bi-multi	NA	NA	NA	0.1 (1.2)	0.0 (0.0)	0.0 (0.0)	0.0 (0.0)	0.7 (5.1)	1.1 (8.4)
	Multi	NA	NA	NA	4.7 (86.6)	5.9 (81.0)	7.1 (95.7)	5.9 (94.8)	6.1 (44.6)	6.0 (46.6)
Iceland	Bi	1.2 (42.7)	1.9 (65.1)	2.9 (68.8)	2.3 (70.5)	1.4 (61.7)	1.7 (62.9)	0.3 (17.8)	0.6 (17.8)	1.7 (36.0)
	Bi-multi	0.8 (29.8)	0.5 (16.7)	0.8 (17.9)	0.5 (15.4)	0.1 (4.6)	0.2 (8.2)	0.3 (15.8)	1.7 (53.5)	2.4 (51.4)
	Multi	0.8 (27.5)	0.5 (18.2)	0.6 (13.2)	0.5 (14.1)	0.8 (33.6)	0.8 (28.9)	1.1 (66.4)	0.9 (28.7)	0.6 (12.5)
Ireland	Bi	79.7 (63.4)	75.6 (62.7)	78.7 (60.2)	77.2 (60.1)	64.0 (59.6)	56.5 (52.1)	61.3 (56.5)	53.4 (54.7)	57.5 (54.0)
	Bi-multi	8.9 (7.1)	6.9 (5.7)	9.6 (7.4)	16.0 (12.5)	9.1 (8.5)	14.9 (13.8)	11.6 (10.7)	12.7 (13.0)	15.0 (14.1)
	Multi	37.2 (29.6)	38.2 (31.6)	42.4 (32.4)	35.2 (27.4)	34.2 (31.9)	37.0 (34.1)	35.6 (32.8)	31.6 (32.3)	34.0 (31.9)
Italy	Bi	70.8 (41.4)	38.7 (29.4)	43.7 (27.5)	55.2 (28.2)	76.8 (35.3)	62.3 (26.1)	54.3 (19.7)	89.3 (30.2)	86.1 (26.3)
	Bi-multi	5.3 (3.1)	6.1 (4.7)	5.0 (3.1)	4.0 (2.0)	3.5 (1.6)	6.0 (2.5)	70.1 (25.4)	25.6 (8.6)	27.4 (8.4)
	Multi	94.9 (55.5)	86.7 (65.9)	110.2 (69.3)	136.3 (69.7)	137.6 (63.1)	170.9 (71.4)	151.2 (54.9)	181.2 (61.2)	213.7 (65.3)
Japan	Bi	265.3 (38.1)	292.6 (34.2)	221.7 (30.9)	239.3 (28.0)	363.6 (42.0)	359.0 (40.4)	444.6 (40.6)	284.6 (30.0)	280.3 (27.5)
	Bi-multi	64.2 (9.2)	94.8 (11.1)	174.0 (24.3)	142.2 (16.6)	69.7 (8.0)	107.6 (12.1)	96.2 (8.8)	101.3 (10.7)	120.8 (11.8)
	Multi	367.4 (52.7)	468.3 (54.7)	321.4 (44.8)	474.0 (55.4)	432.8 (50.0)	422.2 (47.5)	555.4 (50.7)	562.6 (59.3)	620.0 (60.7)
Luxembourg	Bi	27.4 (43.5)	29.3 (46.6)	34.4 (48.3)	33.1 (49.4)	28.7 (47.6)	31.6 (49.7)	26.7 (43.4)	24.3 (39.5)	32.9 (48.1)
	Bi-multi	10.8 (17.1)	9.7 (15.4)	12.8 (18.0)	11.0 (16.4)	10.2 (16.8)	8.5 (13.3)	9.8 (15.9)	12.4 (20.1)	25.4 (37.1)
	Multi	24.8 (39.4)	24.0 (38.1)	24.1 (33.8)	23.0 (34.2)	21.5 (35.6)	23.4 (36.9)	25.0 (40.7)	24.9 (40.4)	10.0 (14.7)
Netherlands	Bi	221.9 (39.9)	233.1 (47.8)	194.8 (36.4)	198.9 (39.5)	194.6 (39.8)	219.6 (38.6)	193.1 (36.8)	238.9 (39.1)	198.8 (38.0)
	Bi-multi	54.3 (9.8)	23.9 (4.9)	47.9 (9.0)	58.4 (11.6)	62.8 (12.8)	89.0 (15.6)	85.9 (16.4)	131.3 (21.5)	100.2 (19.1)
	Multi	280.0 (50.3)	231.0 (47.3)	292.2 (54.6)	247.0 (49.0)	231.5 (47.3)	260.6 (45.8)	245.1 (46.8)	241.3 (39.5)	224.6 (42.9)
New Zealand	Bi	17.2 (46.9)	18.3 (51.6)	19.7 (60.4)	15.4 (53.8)	11.7 (52.7)	10.6 (53.7)	12.4 (60.6)	13.2 (52.5)	17.4 (72.3)
	Bi-multi	8.6 (23.6)	7.8 (22.0)	2.9 (9.0)	5.4 (18.7)	3.8 (17.1)	2.4 (12.1)	1.7 (8.2)	5.4 (21.6)	1.9 (7.9)
	Multi	10.8 (29.5)	9.3 (26.4)	10.0 (30.6)	7.9 (27.5)	6.7 (30.3)	6.7 (34.2)	6.4 (31.2)	6.5 (25.9)	4.8 (19.8)
Norway	Bi	117.7 (31.1)	102.4 (26.2)	120.8 (25.4)	117.3 (23.2)	121.3 (23.2)	98.1 (19.3)	109.1 (20.3)	87.3 (16.9)	76.4 (15.2)
	Bi-multi	35.9 (9.5)	50.0 (12.8)	99.9 (21.0)	114.9 (22.7)	109.4 (20.9)	132.3 (26.0)	145.5 (27.0)	161.2 (31.3)	167.3 (33.3)
	Multi	225.4 (59.5)	238.9 (61.1)	254.2 (53.5)	274.1 (54.1)	291.6 (55.8)	278.1 (54.7)	284.0 (52.7)	266.4 (51.7)	258.4 (51.5)
Poland	Bi	NA	NA	1.1 (7.4)	0.7 (5.3)	1.1 (6.4)	1.1 (4.5)	0.9 (3.8)	1.4 (7.4)	12.7 (35.5)
	Bi-multi	NA	NA	0.3 (2.0)	0.0 (0.0)	0.0 (0.2)	0.0 (0.2)	0.1 (0.5)	0.1 (0.4)	0.1 (0.3)
	Multi	NA	NA	13.8 (90.6)	13.2 (94.7)	16.6 (93.4)	23.3 (95.3)	23.5 (95.7)	17.0 (92.2)	22.9 (64.2)
Portugal	Bi	15.3 (57.3)	14.0 (72.5)	15.4 (60.4)	17.8 (76.2)	22.2 (69.0)	12.9 (56.5)	4.8 (24.8)	7.8 (46.7)	12.0 (52.4)
	Bi-multi	0.2 (0.7)	0.2 (1.0)	0.1 (0.5)	0.1 (0.5)	2.3 (7.3)	0.1 (0.3)	0.2 (0.9)	0.1 (0.6)	0.0 (0.1)

	Multi	11.3 (42.1)	5.1 (26.5)	10.0 (39.1)	5.5 (23.3)	7.6 (23.8)	9.9 (43.3)	14.4 (74.3)	8.8 (52.7)	10.9 (47.4)
Slovak Republic	Bi	NA	NA	0.7 (20.9)	0.4 (13.4)	0.3 (7.6)	0.7 (16.4)	1.7 (26.0)	1.5 (29.7)	0.6 (11.8)
	Bi-multi	NA	NA	0.0 (0.4)	0.1 (1.8)	0.0 (0.0)	0.0 (0.0)	0.5 (7.4)	0.1 (2.3)	0.5 (10.8)
	Multi	NA	NA	2.5 (78.7)	2.5 (84.7)	3.6 (92.4)	3.7 (83.6)	4.4 (66.6)	3.4 (68.0)	3.8 (77.5)
Slovenia	Bi	0.4 (18.1)	1.4 (44.4)	0.9 (33.2)	0.1 (8.5)	0.2 (9.8)	0.3 (9.5)	0.2 (8.1)	0.2 (8.5)	0.2 (6.9)
	Bi-multi	0.0 (1.4)	0.0 (0.0)	0.0 (0.0)	0.0 (0.0)	0.0 (0.0)	0.0 (0.0)	0.0 (0.2)	0.0 (1.5)	0.0 (1.2)
	Multi	1.8 (80.5)	1.8 (55.6)	1.8 (66.8)	1.5 (91.5)	1.9 (90.2)	2.4 (90.5)	2.8 (91.7)	1.7 (90.0)	2.2 (92.0)
South Korea	Bi	101.2 (72.5)	140.9 (78.0)	145.5 (72.6)	140.7 (71.3)	134.2 (64.0)	176.3 (71.5)	163.7 (67.6)	151.0 (66.1)	154.1 (67.9)
	Bi-multi	7.4 (5.3)	2.1 (1.2)	12.9 (6.4)	23.9 (12.1)	37.7 (18.0)	33.4 (13.5)	38.4 (15.8)	40.9 (17.9)	33.6 (14.8)
	Multi	30.9 (22.2)	37.7 (20.9)	42.1 (21.0)	32.9 (16.7)	37.7 (18.0)	36.9 (15.0)	40.3 (16.6)	36.7 (16.1)	39.1 (17.2)
Spain	Bi	110.7 (46.9)	59.7 (55.9)	61.6 (49.1)	49.8 (47.7)	34.7 (39.2)	51.6 (36.4)	49.1 (31.0)	50.2 (40.1)	45.5 (35.5)
	Bi-multi	30.1 (12.8)	12.3 (11.5)	6.1 (4.9)	6.7 (6.4)	5.9 (6.7)	2.2 (1.5)	5.0 (3.2)	2.4 (2.0)	2.8 (2.2)
	Multi	95.0 (40.3)	34.7 (32.5)	57.8 (46.1)	47.9 (45.9)	47.9 (54.1)	87.9 (62.1)	104.3 (65.9)	72.5 (58.0)	79.8 (62.3)
Sweden	Bi	97.8 (22.3)	115.5 (24.8)	120.4 (25.1)	100.1 (23.2)	96.4 (21.0)	104.6 (22.5)	125.1 (24.6)	125.7 (24.6)	133.4 (28.8)
	Bi-multi	52.6 (12.0)	106.3 (22.8)	73.7 (15.4)	86.5 (20.0)	78.0 (17.0)	90.2 (19.4)	110.1 (21.6)	106.5 (20.9)	111.0 (23.9)
	Multi	288.2 (65.7)	244.5 (52.4)	284.7 (59.5)	245.8 (56.8)	284.7 (62.0)	269.7 (58.1)	274.0 (53.8)	278.1 (54.5)	219.0 (47.3)
Switzerland	Bi	63.0 (44.2)	64.5 (44.7)	72.6 (49.1)	99.8 (48.6)	91.6 (46.1)	81.4 (44.0)	102.3 (47.8)	98.2 (48.4)	105.2 (52.2)
	Bi-multi	6.4 (4.5)	8.1 (5.6)	13.4 (9.1)	27.3 (13.3)	22.4 (11.3)	23.6 (12.7)	28.8 (13.4)	27.6 (13.6)	29.6 (14.7)
	Multi	73.1 (51.3)	71.8 (49.7)	61.7 (41.8)	78.2 (38.1)	84.7 (42.7)	80.1 (43.3)	83.1 (38.8)	77.3 (38.1)	66.6 (33.1)
United Kingdom	Bi	882.6 (42.9)	1089.5 (49.9)	1115.6 (34.1)	1109.4 (39.3)	987.9 (44.4)	994.0 (45.3)	1207.1 (43.0)	1173.1 (40.8)	1213.2 (42.4)
	Bi-multi	507.2 (24.7)	467.0 (21.4)	706.1 (21.6)	631.7 (22.4)	421.4 (18.9)	433.4 (19.7)	561.7 (20.0)	593.3 (20.6)	660.5 (23.1)
	Multi	666.4 (32.4)	626.7 (28.7)	1454.1 (44.4)	1078.8 (38.3)	814.8 (36.6)	767.1 (35.0)	1038.5 (37.0)	1107.9 (38.5)	988.9 (34.5)
United States	Bi	8069.7 (81.5)	7743.0 (78.3)	8016.6 (78.4)	7806.4 (74.6)	7595.4 (82.9)	8264.1 (74.4)	8834.9 (80.1)	8393.1 (81.8)	5910.9 (73.4)
	Bi-multi	295.3 (3.0)	279.5 (2.8)	345.8 (3.4)	393.6 (3.8)	452.8 (4.9)	398.8 (3.6)	572.5 (5.2)	355.0 (3.5)	622.1 (7.7)
	Multi	1541.8 (15.6)	1867.5 (18.9)	1862.9 (18.2)	2263.2 (21.6)	1119.0 (12.2)	2443.8 (22.0)	1627.8 (14.8)	1513.5 (14.7)	1516.7 (18.8)
All members	Bi	11455.4 (61.6)	11330.5 (60.6)	11756.4 (57.9)	11749.6 (57.8)	11217.4 (61.6)	12060.6 (59.0)	12806.2 (59.7)	12350.2 (59.5)	9986.0 (53.9)
	Bi-multi	1573.4 (8.5)	1601.9 (8.6)	1965.6 (9.7)	1968.0 (9.7)	1590.7 (8.7)	1651.0 (8.1)	2221.8 (10.4)	2184.8 (10.5)	2376.6 (12.8)
	Multi	5578.5 (30.0)	5752.3 (30.8)	6593.6 (32.5)	6614.2 (32.5)	5391.9 (29.6)	6713.7 (32.9)	6419.8 (29.9)	6234.4 (30.0)	6172.5 (33.3)

DAH: development assistance for health; DAH: Development Assistance Committee

Table S4: Estimated DAH in constant prices at 2019 from all the 29 DAC member countries, channeled through multilateral agencies (%), 2011–2019

Country	Agency	2011	2012	2013	2014	2015	2016	2017	2018	2019
Australia	Global Fund	30.8 (13.1)	46.3 (15.4)	76.4 (31.6)	26.7 (10.3)	52.9 (33.0)	69.1 (31.4)	0.0 (0.0)	0.0 (0.0)	0.0 (0.0)
	World Bank	28.2 (12.0)	99.7 (33.2)	34.5 (14.3)	29.4 (11.4)	27.3 (17.0)	30.2 (13.8)	26.8 (23.5)	38.3 (27.4)	25.8 (18.0)
	WHO	52.5 (22.3)	47.0 (15.7)	38.4 (15.9)	38.4 (14.8)	40.7 (25.4)	42.8 (19.5)	27.1 (23.8)	31.1 (22.3)	15.4 (10.7)
	Gavi	31.6 (13.4)	39.8 (13.3)	33.7 (13.9)	73.1 (28.3)	0.0 (0.0)	52.1 (23.7)	29.0 (25.4)	15.2 (10.9)	73.0 (51.0)
	UNFPA	23.1 (9.8)	13.2 (4.4)	11.5 (4.7)	13.7 (5.3)	7.6 (4.7)	9.8 (4.5)	3.6 (3.1)	10.3 (7.4)	8.0 (5.6)
	EU Institutions	0.0 (0.0)	0.0 (0.0)	0.0 (0.0)	0.0 (0.0)	0.0 (0.0)	0.0 (0.0)	0.0 (0.0)	0.0 (0.0)	0.0 (0.0)
	UNICEF	19.0 (8.1)	11.0 (3.7)	0.0 (0.0)	11.1 (4.3)	6.5 (4.1)	5.0 (2.3)	4.8 (4.2)	4.2 (3.0)	2.5 (1.8)
	UNAIDS	6.0 (2.6)	5.8 (1.9)	3.7 (1.5)	2.5 (0.9)	3.7 (2.3)	2.6 (1.2)	2.5 (2.2)	3.9 (2.8)	0.3 (0.2)
	Regional DB	18.0 (7.6)	3.0 (1.0)	12.1 (5.0)	33.2 (12.8)	3.2 (2.0)	2.0 (0.9)	17.8 (15.5)	19.9 (14.3)	3.8 (2.6)
	Others	26.2 (11.2)	34.2 (11.4)	31.3 (13.0)	30.5 (11.8)	18.2 (11.3)	6.0 (2.8)	2.7 (2.3)	16.5 (12.0)	14.4 (10.1)
Austria	Global Fund	0.0 (0.0)	0.0 (0.0)	0.0 (0.0)	0.0 (0.0)	0.0 (0.0)	0.0 (0.0)	0.0 (0.0)	0.0 (0.0)	0.0 (0.0)
	World Bank	15.3 (44.6)	16.0 (52.3)	15.4 (47.1)	12.0 (38.7)	14.9 (44.5)	14.3 (42.1)	20.4 (47.4)	17.7 (53.2)	20.1 (50.1)
	WHO	2.7 (8.0)	2.8 (9.0)	3.0 (9.3)	2.7 (8.7)	2.9 (8.7)	2.7 (8.1)	2.9 (6.7)	3.0 (8.9)	2.6 (6.4)
	Gavi	0.0 (0.0)	0.0 (0.0)	0.0 (0.0)	0.0 (0.0)	0.0 (0.0)	0.0 (0.0)	0.0 (0.0)	0.0 (0.0)	0.0 (0.0)
	UNFPA	0.8 (2.4)	0.1 (0.2)	0.2 (0.6)	0.0 (0.0)	0.0 (0.0)	0.0 (0.1)	0.0 (0.0)	0.1 (0.3)	0.1 (0.2)
	EU Institutions	10.7 (31.3)	8.1 (26.4)	10.3 (31.5)	11.0 (35.5)	10.7 (31.9)	13.8 (40.6)	17.1 (39.8)	10.1 (30.3)	14.0 (35.0)
	UNICEF	0.8 (2.3)	0.1 (0.2)	0.7 (2.1)	0.2 (0.7)	0.2 (0.6)	0.2 (0.6)	0.2 (0.5)	0.2 (0.5)	0.2 (0.5)
	UNAIDS	0.1 (0.3)	0.0 (0.0)	0.0 (0.0)	0.0 (0.0)	0.0 (0.0)	0.0 (0.0)	0.0 (0.0)	0.0 (0.0)	0.0 (0.0)
	Regional DB	2.5 (7.4)	1.8 (6.0)	1.2 (3.7)	3.2 (10.2)	3.2 (9.5)	1.1 (3.1)	0.6 (1.4)	0.9 (2.8)	0.7 (1.7)
	Others	1.3 (3.8)	1.8 (5.9)	1.9 (5.7)	1.9 (6.3)	1.6 (4.8)	1.8 (5.4)	1.8 (4.4)	1.3 (4.0)	2.4 (6.1)
Belgium	Global Fund	26.5 (26.2)	26.0 (30.5)	14.1 (17.8)	23.0 (25.3)	22.7 (29.2)	22.3 (27.9)	17.3 (18.7)	17.1 (21.8)	16.8 (22.3)
	World Bank	17.0 (16.8)	16.4 (19.2)	15.9 (20.0)	12.6 (13.9)	2.5 (3.2)	3.1 (3.9)	6.8 (7.4)	17.3 (22.1)	16.9 (22.4)
	WHO	18.6 (18.4)	14.7 (17.2)	12.6 (15.8)	16.5 (18.1)	14.1 (18.2)	11.8 (14.8)	13.7 (14.8)	7.6 (9.7)	7.1 (9.4)
	Gavi	0.0 (0.0)	0.0 (0.0)	0.0 (0.0)	0.0 (0.0)	0.0 (0.0)	0.0 (0.0)	0.0 (0.0)	0.0 (0.0)	0.0 (0.0)
	UNFPA	6.3 (6.2)	7.5 (8.8)	8.0 (10.0)	8.8 (9.6)	5.6 (7.1)	9.3 (11.7)	13.2 (14.3)	10.8 (13.8)	6.8 (9.1)
	EU Institutions	17.5 (17.3)	13.0 (15.2)	16.8 (21.1)	16.9 (18.6)	18.6 (23.9)	23.3 (29.1)	32.2 (34.8)	16.9 (21.6)	23.1 (30.7)
	UNICEF	5.1 (5.0)	3.7 (4.3)	2.4 (3.0)	4.3 (4.7)	5.2 (6.7)	3.2 (4.0)	2.8 (3.1)	2.5 (3.2)	2.9 (3.9)
	UNAIDS	5.1 (5.0)	1.0 (1.1)	5.7 (7.2)	3.4 (3.7)	4.2 (5.4)	4.5 (5.6)	3.5 (3.8)	4.0 (5.1)	0.4 (0.5)
	Regional DB	3.3 (3.2)	0.2 (0.2)	1.0 (1.3)	2.9 (3.1)	3.0 (3.9)	0.8 (1.0)	0.1 (0.1)	0.2 (0.2)	0.1 (0.2)
	Others	2.0 (2.0)	2.9 (3.4)	3.0 (3.8)	2.7 (2.8)	2.0 (2.5)	1.8 (2.2)	2.8 (3.0)	1.9 (2.5)	1.2 (1.7)
Canada	Global Fund	151.5 (27.1)	166.3 (28.5)	147.1 (27.9)	0.0 (0.0)	350.1 (56.8)	173.9 (34.3)	211.9 (37.9)	206.0 (35.2)	200.2 (38.3)
	World Bank	78.0 (13.9)	78.7 (13.5)	77.5 (14.7)	44.2 (14.7)	62.1 (10.1)	100.4 (19.8)	100.0 (17.9)	115.4 (19.7)	102.5 (19.6)
	WHO	108.5 (19.4)	112.6 (19.3)	92.4 (17.5)	59.0 (19.6)	45.5 (7.4)	42.0 (8.3)	43.9 (7.9)	28.3 (4.8)	31.3 (6.0)
	Gavi	15.4 (2.7)	11.7 (2.0)	31.5 (6.0)	32.1 (10.7)	7.6 (1.2)	73.1 (14.4)	88.0 (15.7)	71.2 (12.2)	82.5 (15.8)
	UNFPA	39.6 (7.1)	27.4 (4.7)	34.4 (6.5)	37.8 (12.5)	34.9 (5.7)	9.8 (1.9)	31.9 (5.7)	59.5 (10.2)	39.9 (7.6)
	EU Institutions	0.0 (0.0)	0.0 (0.0)	0.0 (0.0)	0.0 (0.0)	0.0 (0.0)	0.0 (0.0)	0.0 (0.0)	0.0 (0.0)	0.0 (0.0)
	UNICEF	81.4 (14.6)	69.3 (11.9)	71.5 (13.5)	56.7 (18.8)	58.6 (9.5)	66.5 (13.1)	57.0 (10.2)	54.2 (9.3)	37.0 (7.1)
	UNAIDS	6.2 (1.1)	3.3 (0.6)	2.8 (0.5)	1.7 (0.6)	2.5 (0.4)	3.0 (0.6)	2.9 (0.5)	0.0 (0.0)	0.8 (0.2)
	Regional DB	8.7 (1.6)	4.7 (0.8)	3.5 (0.7)	9.9 (3.3)	9.3 (1.5)	4.5 (0.9)	4.0 (0.7)	2.7 (0.5)	3.0 (0.6)
	Others	70.1 (12.4)	110.1 (18.9)	67.1 (12.8)	59.7 (19.8)	45.5 (7.3)	34.3 (6.8)	19.0 (3.5)	48.1 (8.2)	25.1 (4.8)
Czech Republic	Global Fund	0.0 (0.0)	0.0 (0.0)	0.0 (0.0)	0.0 (0.0)	0.0 (0.0)	0.0 (0.0)	0.0 (0.0)	0.0 (0.0)	0.0 (0.0)
	World Bank	1.4 (19.4)	1.5 (23.2)	0.7 (9.1)	0.5 (10.9)	0.7 (10.6)	0.7 (7.6)	0.6 (5.4)	0.6 (8.3)	0.9 (10.1)
	WHO	1.1 (14.8)	1.1 (17.7)	2.5 (32.9)	0.1 (1.4)	1.4 (20.1)	1.4 (14.5)	1.6 (14.1)	1.1 (16.4)	1.1 (11.9)
	Gavi	0.0 (0.0)	0.0 (0.0)	0.0 (0.0)	0.0 (0.0)	0.0 (0.0)	0.0 (0.0)	0.0 (0.0)	0.0 (0.0)	0.0 (0.0)

	UNFPA	0.0 (0.0)	0.1 (1.2)	0.1 (1.3)	0.0 (0.5)	0.1 (1.1)	0.0 (0.3)	0.0 (0.3)	0.0 (0.0)	0.0 (0.0)
	EU Institutions	4.7 (65.4)	3.4 (53.9)	4.2 (55.8)	4.3 (86.2)	4.6 (67.5)	7.3 (77.0)	9.2 (80.0)	4.9 (74.1)	7.0 (77.5)
	UNICEF	0.0 (0.0)	0.0 (0.0)	0.0 (0.0)	0.0 (0.0)	0.0 (0.0)	0.0 (0.0)	0.0 (0.0)	0.0 (0.0)	0.0 (0.0)
	UNAIDS	0.0 (0.0)	0.0 (0.6)	0.0 (0.4)	0.0 (0.4)	0.0 (0.4)	0.0 (0.4)	0.0 (0.0)	0.0 (0.0)	0.0 (0.0)
	Regional DB	0.0 (0.0)	0.0 (0.0)	0.0 (0.0)	0.0 (0.0)	0.0 (0.0)	0.0 (0.0)	0.0 (0.0)	0.0 (0.0)	0.0 (0.0)
	Others	0.0 (0.4)	0.2 (3.5)	0.0 (0.4)	0.0 (0.6)	0.0 (0.3)	0.0 (0.2)	0.0 (0.2)	0.1 (1.1)	0.1 (0.6)
Denmark	Global Fund	28.5 (24.6)	23.1 (20.8)	22.9 (22.1)	25.7 (19.2)	15.5 (22.6)	7.7 (11.2)	0.0 (0.0)	22.8 (17.0)	22.5 (19.2)
	World Bank	9.0 (7.8)	9.6 (8.7)	7.0 (6.8)	5.7 (4.3)	9.9 (14.5)	10.5 (15.2)	15.9 (15.5)	10.0 (7.4)	20.0 (17.1)
	WHO	7.0 (6.0)	6.9 (6.2)	6.8 (6.6)	7.0 (5.3)	6.1 (8.9)	5.8 (8.4)	5.4 (5.2)	8.1 (6.1)	6.1 (5.2)
	Gavi	3.7 (3.2)	3.7 (3.4)	3.7 (3.5)	0.0 (0.0)	0.0 (0.0)	0.0 (0.0)	0.0 (0.0)	3.5 (2.6)	3.5 (3.0)
	UNFPA	37.6 (32.5)	39.8 (35.9)	39.1 (37.9)	58.5 (43.7)	14.0 (20.3)	21.5 (31.1)	47.7 (46.4)	47.8 (35.7)	41.3 (35.3)
	EU Institutions	8.7 (7.6)	6.7 (6.0)	8.5 (8.2)	8.2 (6.1)	9.3 (13.5)	10.8 (15.5)	18.8 (18.3)	19.6 (14.6)	11.5 (9.9)
	UNICEF	5.7 (4.9)	3.7 (3.4)	4.0 (3.9)	10.3 (7.7)	2.1 (3.1)	2.9 (4.2)	7.2 (7.0)	0.9 (0.7)	1.4 (1.2)
	UNAIDS	4.6 (4.0)	4.7 (4.2)	4.2 (4.1)	3.1 (2.3)	4.5 (6.6)	3.6 (5.2)	3.5 (3.4)	5.6 (4.2)	0.6 (0.6)
	Regional DB	1.4 (1.2)	1.2 (1.0)	1.0 (1.0)	0.7 (0.5)	2.0 (3.0)	1.1 (1.6)	0.3 (0.3)	0.2 (0.2)	0.5 (0.4)
	Others	9.3 (8.1)	11.6 (10.5)	6.1 (5.9)	14.8 (11.0)	5.1 (7.5)	5.3 (7.5)	3.9 (3.9)	15.2 (11.3)	9.5 (8.0)
Finland	Global Fund	5.1 (6.0)	5.0 (5.9)	2.4 (2.8)	0.0 (0.0)	0.0 (0.0)	0.0 (0.0)	0.0 (0.0)	0.0 (0.0)	0.0 (0.0)
	World Bank	7.7 (8.9)	7.0 (8.2)	8.4 (9.5)	10.4 (8.3)	12.2 (15.5)	4.4 (10.6)	7.3 (16.8)	8.2 (22.9)	6.0 (17.8)
	WHO	8.7 (10.1)	10.1 (11.9)	9.2 (10.4)	9.3 (7.4)	8.5 (10.7)	3.3 (7.9)	2.9 (6.8)	3.3 (9.1)	2.8 (8.3)
	Gavi	0.0 (0.0)	0.0 (0.0)	0.0 (0.0)	0.0 (0.0)	0.0 (0.0)	0.0 (0.0)	0.0 (0.0)	0.0 (0.0)	0.0 (0.0)
	UNFPA	39.2 (45.4)	38.3 (44.9)	43.3 (49.1)	57.5 (45.7)	26.0 (33.0)	13.8 (33.3)	12.8 (29.6)	10.1 (28.3)	8.9 (26.5)
	EU Institutions	7.3 (8.4)	5.5 (6.4)	6.7 (7.6)	9.1 (7.2)	10.3 (13.1)	10.2 (24.7)	12.8 (29.7)	9.1 (25.5)	10.7 (32.0)
	UNICEF	4.1 (4.8)	3.0 (3.5)	3.8 (4.3)	15.9 (12.6)	6.5 (8.3)	4.8 (11.6)	1.3 (3.1)	1.1 (3.2)	1.4 (4.0)
	UNAIDS	8.4 (9.7)	8.8 (10.3)	7.8 (8.8)	5.1 (4.1)	6.1 (7.8)	0.0 (0.0)	0.0 (0.0)	0.0 (0.0)	0.0 (0.0)
	Regional DB	1.6 (1.8)	1.6 (1.9)	1.0 (1.1)	4.9 (3.9)	2.8 (3.6)	1.1 (2.6)	0.3 (0.6)	0.5 (1.4)	0.3 (0.9)
	Others	4.2 (4.8)	6.0 (7.1)	5.6 (6.4)	13.6 (10.9)	6.3 (8.1)	3.8 (9.3)	5.7 (13.2)	3.4 (9.6)	3.6 (10.7)
France	Global Fund	359.7 (59.2)	426.7 (70.3)	399.9 (66.1)	435.0 (71.8)	369.5 (61.8)	355.0 (63.0)	442.3 (68.1)	408.7 (62.6)	436.6 (69.1)
	World Bank	65.0 (10.7)	47.7 (7.9)	47.9 (7.9)	38.0 (6.3)	83.5 (14.0)	39.6 (7.0)	47.7 (7.3)	133.0 (20.4)	44.3 (7.0)
	WHO	23.2 (3.8)	29.1 (4.8)	28.2 (4.7)	26.4 (4.4)	38.0 (6.4)	26.9 (4.8)	28.2 (4.3)	25.5 (3.9)	22.3 (3.5)
	Gavi	53.1 (8.7)	17.6 (2.9)	28.3 (4.7)	6.4 (1.1)	0.0 (0.0)	23.1 (4.1)	0.0 (0.0)	0.0 (0.0)	0.0 (0.0)
	UNFPA	7.1 (1.2)	6.4 (1.1)	6.0 (1.0)	6.1 (1.0)	5.4 (0.9)	4.4 (0.8)	1.2 (0.2)	3.7 (0.6)	4.5 (0.7)
	EU Institutions	76.6 (12.6)	58.0 (9.6)	74.1 (12.3)	70.7 (11.7)	75.4 (12.6)	100.5 (17.8)	121.2 (18.7)	70.6 (10.8)	96.6 (15.3)
	UNICEF	10.5 (1.7)	10.8 (1.8)	10.9 (1.8)	10.9 (1.8)	9.1 (1.5)	7.0 (1.2)	3.2 (0.5)	5.1 (0.8)	8.6 (1.4)
	UNAIDS	0.6 (0.1)	0.5 (0.1)	0.5 (0.1)	0.3 (0.0)	0.2 (0.0)	0.4 (0.1)	0.3 (0.1)	0.4 (0.1)	0.0 (0.0)
	Regional DB	9.4 (1.5)	6.7 (1.1)	4.4 (0.7)	9.5 (1.6)	14.2 (2.4)	3.4 (0.6)	3.4 (0.5)	2.9 (0.5)	1.6 (0.3)
	Others	2.7 (0.4)	3.0 (0.5)	4.4 (0.8)	2.6 (0.5)	2.6 (0.4)	2.9 (0.4)	2.1 (0.3)	2.9 (0.4)	17.4 (2.7)
Germany	Global Fund	254.0 (45.6)	250.3 (52.4)	245.4 (48.3)	295.1 (55.9)	248.3 (53.4)	245.8 (44.2)	266.4 (35.4)	268.1 (36.8)	291.1 (40.2)
	World Bank	130.1 (23.3)	69.2 (14.5)	71.6 (14.1)	47.4 (9.0)	2.8 (0.6)	55.4 (10.0)	71.6 (9.5)	161.7 (22.2)	66.1 (9.1)
	WHO	30.4 (5.5)	33.2 (7.0)	28.1 (5.5)	22.3 (4.2)	22.6 (4.9)	31.6 (5.7)	77.5 (10.3)	92.2 (12.7)	75.1 (10.4)
	Gavi	7.0 (1.2)	11.7 (2.5)	32.3 (6.4)	20.3 (3.8)	44.5 (9.6)	64.0 (11.5)	145.5 (19.3)	63.3 (8.7)	63.4 (8.8)
	UNFPA	21.7 (3.9)	21.3 (4.5)	23.3 (4.6)	24.7 (4.7)	15.8 (3.4)	16.5 (3.0)	14.0 (1.9)	12.1 (1.7)	11.3 (1.6)
	EU Institutions	90.7 (16.3)	71.0 (14.9)	89.2 (17.6)	89.2 (16.9)	98.1 (21.1)	113.8 (20.4)	151.7 (20.2)	88.4 (12.1)	123.1 (17.0)
	UNICEF	2.9 (0.5)	1.8 (0.4)	1.4 (0.3)	2.6 (0.5)	1.5 (0.3)	1.8 (0.3)	2.8 (0.4)	10.2 (1.4)	23.3 (3.2)
	UNAIDS	3.2 (0.6)	3.1 (0.7)	3.2 (0.6)	3.0 (0.6)	3.0 (0.6)	2.3 (0.4)	5.8 (0.8)	5.7 (0.8)	1.1 (0.2)
	Regional DB	11.4 (2.0)	9.3 (1.9)	6.0 (1.2)	15.3 (2.9)	16.1 (3.5)	4.5 (0.8)	1.7 (0.2)	3.5 (0.5)	1.9 (0.3)
	Others	6.0 (1.0)	6.5 (1.4)	7.2 (1.4)	8.2 (1.5)	12.1 (2.6)	21.0 (3.7)	15.5 (2.2)	23.4 (3.2)	68.2 (9.5)
Greece	Global Fund	0.0 (0.0)	0.0 (0.0)	0.0 (0.0)	0.0 (0.0)	0.0 (0.0)	0.0 (0.0)	0.0 (0.0)	0.0 (0.0)	0.0 (0.0)

	World Bank	0.0 (0.0)	0.0 (0.0)	0.0 (0.0)	0.0 (0.0)	0.0 (0.0)	0.0 (0.0)	1.7 (13.5)	1.2 (14.0)	0.5 (5.7)
	WHO	1.8 (20.0)	0.8 (13.3)	3.1 (35.7)	2.0 (27.6)	2.0 (24.7)	2.1 (21.3)	1.5 (12.0)	1.4 (17.4)	0.0 (0.0)
	Gavi	0.0 (0.0)	0.0 (0.0)	0.0 (0.0)	0.0 (0.0)	0.0 (0.0)	0.0 (0.0)	0.0 (0.0)	0.0 (0.0)	0.0 (0.0)
	UNFPA	0.0 (0.0)	0.0 (0.0)	0.0 (0.0)	0.0 (0.0)	0.0 (0.0)	0.0 (0.0)	0.0 (0.0)	0.0 (0.0)	0.0 (0.0)
	EU Institutions	7.3 (80.0)	5.2 (86.7)	5.5 (63.4)	5.3 (71.9)	6.0 (75.3)	7.8 (78.7)	9.5 (74.3)	5.6 (67.8)	7.6 (93.5)
	UNICEF	0.0 (0.0)	0.0 (0.0)	0.0 (0.0)	0.0 (0.0)	0.0 (0.0)	0.0 (0.0)	0.0 (0.2)	0.0 (0.0)	0.0 (0.0)
	UNAIDS	0.0 (0.0)	0.0 (0.0)	0.0 (0.0)	0.0 (0.0)	0.0 (0.0)	0.0 (0.0)	0.0 (0.0)	0.0 (0.0)	0.0 (0.0)
	Regional DB	0.0 (0.0)	0.0 (0.0)	0.0 (0.0)	0.0 (0.0)	0.0 (0.0)	0.0 (0.0)	0.0 (0.0)	0.0 (0.0)	0.0 (0.0)
	Others	0.0 (0.0)	0.0 (0.0)	0.1 (0.9)	0.0 (0.5)	0.0 (0.0)	0.0 (0.0)	0.0 (0.0)	0.1 (0.8)	0.1 (0.9)
Hungary	Global Fund	NA	NA	NA	0.0 (0.0)	0.0 (0.0)	0.0 (0.0)	0.0 (0.0)	0.0 (0.0)	0.0 (0.0)
	World Bank	NA	NA	NA	0.9 (18.3)	1.6 (26.9)	1.3 (17.5)	0.5 (8.9)	1.2 (17.8)	0.5 (6.9)
	WHO	NA	NA	NA	0.9 (18.9)	0.9 (14.9)	1.0 (13.3)	1.0 (17.5)	1.7 (25.3)	1.7 (23.7)
	Gavi	NA	NA	NA	0.0 (0.0)	0.0 (0.0)	0.0 (0.0)	0.0 (0.0)	0.0 (0.0)	0.0 (0.0)
	UNFPA	NA	NA	NA	0.0 (0.2)	0.0 (0.1)	0.0 (0.0)	0.0 (0.0)	0.0 (0.0)	0.0 (0.0)
	EU Institutions	NA	NA	NA	3.0 (62.6)	3.4 (57.8)	4.9 (69.1)	4.3 (73.7)	3.3 (48.4)	4.9 (68.6)
	UNICEF	NA	NA	NA	0.0 (0.0)	0.0 (0.0)	0.0 (0.0)	0.0 (0.0)	0.5 (7.1)	0.0 (0.2)
	UNAIDS	NA	NA	NA	0.0 (0.0)	0.0 (0.0)	0.0 (0.0)	0.0 (0.0)	0.0 (0.0)	0.0 (0.0)
	Regional DB	NA	NA	NA	0.0 (0.0)	0.0 (0.0)	0.0 (0.0)	0.0 (0.0)	0.0 (0.0)	0.0 (0.0)
	Others	NA	NA	NA	0.0 (0.1)	0.0 (0.4)	0.0 (0.0)	0.0 (0.0)	0.1 (1.3)	0.0 (0.5)
Iceland	Global Fund	0.0 (0.0)	0.0 (0.0)	0.1 (4.3)	0.0 (0.0)	0.0 (0.0)	0.0 (0.0)	0.0 (0.0)	0.0 (0.0)	0.0 (0.0)
	World Bank	0.2 (15.0)	0.2 (20.0)	0.2 (14.9)	0.2 (23.9)	0.5 (60.4)	0.5 (51.6)	0.6 (44.1)	0.6 (22.8)	0.3 (9.9)
	WHO	0.1 (7.4)	0.1 (10.7)	0.2 (12.5)	0.1 (10.3)	0.1 (10.7)	0.1 (10.5)	0.1 (7.3)	0.0 (0.0)	0.0 (0.0)
	Gavi	0.0 (0.0)	0.0 (0.0)	0.0 (0.0)	0.0 (0.0)	0.0 (0.0)	0.0 (0.0)	0.0 (0.0)	1.0 (38.3)	0.0 (0.0)
	UNFPA	0.5 (32.9)	0.2 (15.8)	0.1 (9.2)	0.1 (12.0)	0.1 (12.1)	0.1 (11.3)	0.2 (11.1)	0.1 (4.3)	1.2 (39.7)
	EU Institutions	0.0 (0.0)	0.0 (0.0)	0.0 (0.0)	0.0 (0.0)	0.0 (0.0)	0.0 (0.0)	0.0 (0.0)	0.0 (0.0)	0.0 (0.0)
	UNICEF	0.7 (41.7)	0.4 (39.9)	0.6 (41.9)	0.4 (42.3)	0.1 (15.2)	0.3 (26.1)	0.5 (32.7)	0.5 (19.5)	0.9 (29.0)
	UNAIDS	0.0 (0.0)	0.0 (0.0)	0.0 (0.0)	0.0 (0.0)	0.0 (0.0)	0.0 (0.0)	0.0 (0.0)	0.0 (0.0)	0.0 (0.0)
	Regional DB	0.0 (0.0)	0.0 (0.0)	0.0 (0.0)	0.0 (0.0)	0.0 (0.0)	0.0 (0.0)	0.0 (0.0)	0.0 (0.0)	0.0 (0.0)
	Others	0.0 (3.0)	0.1 (13.6)	0.2 (17.2)	0.1 (11.5)	0.0 (1.5)	0.0 (0.7)	0.0 (4.9)	0.4 (15.2)	0.6 (21.4)
Ireland	Global Fund	12.0 (25.9)	14.8 (32.8)	17.8 (34.3)	16.1 (31.4)	12.6 (29.1)	11.5 (22.2)	11.4 (24.1)	11.3 (25.5)	14.0 (28.5)
	World Bank	2.4 (5.2)	2.7 (6.1)	3.5 (6.7)	1.6 (3.2)	2.5 (5.7)	2.7 (5.1)	3.3 (7.0)	2.7 (6.1)	3.0 (6.2)
	WHO	4.2 (9.2)	4.6 (10.1)	8.1 (15.6)	3.1 (6.1)	3.8 (8.9)	3.1 (6.0)	1.5 (3.1)	1.9 (4.3)	2.2 (4.4)
	Gavi	4.1 (9.0)	3.2 (7.1)	2.6 (5.1)	0.7 (1.4)	3.2 (7.5)	3.1 (6.1)	3.2 (6.7)	3.1 (7.1)	3.2 (6.5)
	UNFPA	5.2 (11.2)	3.9 (8.6)	4.0 (7.6)	4.0 (7.7)	2.6 (6.1)	3.1 (6.0)	2.2 (4.6)	2.9 (6.7)	2.0 (4.1)
	EU Institutions	5.1 (11.2)	3.7 (8.3)	4.5 (8.7)	4.6 (8.9)	5.4 (12.6)	8.3 (15.9)	10.2 (21.5)	6.1 (13.8)	8.6 (17.5)
	UNICEF	5.9 (12.8)	3.0 (6.6)	3.5 (6.7)	10.9 (21.3)	5.2 (11.9)	8.8 (17.0)	5.7 (12.0)	5.4 (12.3)	6.2 (12.7)
	UNAIDS	3.6 (7.8)	3.3 (7.4)	2.9 (5.6)	1.6 (3.2)	2.0 (4.6)	2.3 (4.5)	4.4 (9.2)	2.5 (5.6)	0.7 (1.4)
	Regional DB	0.6 (1.2)	0.0 (0.1)	0.1 (0.2)	0.2 (0.3)	0.1 (0.3)	0.1 (0.2)	0.1 (0.1)	0.2 (0.4)	0.1 (0.3)
	Others	3.0 (6.5)	5.8 (12.8)	5.0 (9.6)	8.5 (16.4)	5.7 (13.4)	8.9 (17.0)	5.6 (11.6)	8.1 (18.2)	9.1 (18.4)
Italy	Global Fund	0.0 (0.0)	0.0 (0.0)	0.0 (0.0)	35.0 (25.0)	34.7 (24.6)	45.7 (25.9)	43.2 (19.5)	48.1 (23.3)	58.5 (24.3)
	World Bank	20.4 (20.4)	19.2 (20.7)	31.9 (27.7)	27.9 (19.9)	24.6 (17.4)	25.4 (14.4)	29.7 (13.4)	34.1 (16.5)	36.8 (15.3)
	WHO	2.7 (2.7)	23.3 (25.1)	17.4 (15.1)	14.8 (10.5)	15.0 (10.7)	16.1 (9.1)	17.1 (7.7)	15.5 (7.5)	13.8 (5.7)
	Gavi	0.0 (0.0)	0.0 (0.0)	0.0 (0.0)	0.0 (0.0)	0.0 (0.0)	4.2 (2.4)	12.6 (5.7)	29.2 (14.1)	29.6 (12.3)
	UNFPA	1.6 (1.6)	0.1 (0.1)	3.8 (3.3)	2.0 (1.5)	1.7 (1.2)	2.5 (1.4)	5.7 (2.6)	5.9 (2.9)	5.7 (2.4)
	EU Institutions	61.5 (61.4)	42.1 (45.3)	52.4 (45.5)	49.9 (35.6)	55.3 (39.2)	72.1 (40.8)	93.7 (42.3)	51.7 (25.0)	70.8 (29.4)
	UNICEF	4.8 (4.8)	3.3 (3.6)	2.2 (1.9)	2.6 (1.8)	0.8 (0.6)	2.5 (1.4)	5.4 (2.4)	4.8 (2.3)	1.2 (0.5)
	UNAIDS	0.0 (0.0)	0.0 (0.0)	0.0 (0.0)	0.0 (0.0)	0.0 (0.0)	0.0 (0.0)	0.6 (0.3)	0.0 (0.0)	0.0 (0.0)

	Regional DB	7.1 (7.1)	2.8 (3.1)	3.9 (3.4)	6.3 (4.5)	5.6 (3.9)	1.8 (1.0)	3.0 (1.4)	2.6 (1.2)	2.9 (1.2)
	Others	2.2 (2.2)	1.9 (2.1)	3.5 (3.0)	1.8 (1.3)	3.4 (2.5)	6.6 (3.8)	10.5 (4.8)	14.9 (7.2)	21.8 (8.9)
Japan	Global Fund	86.3 (20.0)	261.3 (46.4)	114.3 (23.1)	288.1 (46.7)	211.9 (42.2)	179.3 (33.8)	323.9 (49.7)	286.9 (43.2)	347.5 (46.9)
	World Bank	166.2 (38.5)	106.4 (18.9)	101.3 (20.5)	84.0 (13.6)	142.2 (28.3)	144.1 (27.2)	174.8 (26.8)	191.0 (28.8)	201.9 (27.3)
	WHO	48.6 (11.3)	42.6 (7.6)	51.1 (10.3)	61.8 (10.0)	61.4 (12.2)	92.3 (17.4)	60.1 (9.2)	57.3 (8.6)	54.9 (7.4)
	Gavi	6.5 (1.5)	6.5 (1.1)	7.9 (1.6)	8.1 (1.3)	19.6 (3.9)	18.7 (3.5)	19.6 (3.0)	19.2 (2.9)	19.0 (2.6)
	UNFPA	23.4 (5.4)	29.4 (5.2)	24.2 (4.9)	32.8 (5.3)	29.1 (5.8)	27.8 (5.3)	11.9 (1.8)	27.2 (4.1)	11.2 (1.5)
	EU Institutions	0.0 (0.0)	0.0 (0.0)	0.0 (0.0)	0.0 (0.0)	0.0 (0.0)	0.0 (0.0)	0.0 (0.0)	0.0 (0.0)	0.0 (0.0)
	UNICEF	44.7 (10.4)	78.6 (14.0)	166.7 (33.6)	87.2 (14.2)	19.0 (3.8)	50.2 (9.5)	31.6 (4.8)	38.4 (5.8)	43.6 (5.9)
	UNAIDS	1.7 (0.4)	1.9 (0.3)	2.0 (0.4)	1.9 (0.3)	0.9 (0.2)	0.8 (0.2)	0.7 (0.1)	0.8 (0.1)	0.1 (0.0)
	Regional DB	34.0 (7.9)	25.7 (4.6)	17.2 (3.5)	23.9 (3.9)	16.5 (3.3)	7.9 (1.5)	6.9 (1.1)	21.2 (3.2)	20.7 (2.8)
	Others	20.2 (4.7)	10.7 (1.8)	10.8 (2.2)	28.4 (4.6)	1.9 (0.4)	8.5 (1.7)	22.1 (3.3)	22.1 (3.4)	42.0 (5.6)
Luxembourg	Global Fund	0.0 (0.0)	1.3 (3.8)	0.0 (0.0)	3.0 (8.9)	3.0 (9.5)	3.1 (9.7)	3.2 (9.2)	3.1 (8.3)	3.0 (8.5)
	World Bank	2.6 (7.3)	2.5 (7.5)	2.3 (6.1)	1.2 (3.5)	1.6 (5.1)	2.0 (6.3)	2.1 (6.2)	1.9 (5.0)	1.8 (5.1)
	WHO	8.8 (24.8)	7.4 (22.0)	10.8 (29.3)	9.3 (27.4)	9.0 (28.4)	9.7 (30.3)	10.5 (30.1)	9.3 (25.0)	10.4 (29.4)
	Gavi	1.0 (2.7)	1.0 (2.9)	0.9 (2.6)	0.9 (2.7)	0.9 (2.9)	0.9 (2.8)	0.9 (2.6)	0.9 (2.3)	0.9 (2.4)
	UNFPA	9.1 (25.6)	9.6 (28.5)	7.9 (21.4)	9.2 (27.1)	5.7 (18.2)	5.2 (16.4)	5.7 (16.4)	8.9 (24.0)	6.2 (17.6)
	EU Institutions	1.2 (3.5)	0.9 (2.7)	2.6 (6.9)	1.1 (3.2)	1.1 (3.6)	1.5 (4.8)	2.0 (5.6)	1.1 (3.0)	1.5 (4.3)
	UNICEF	4.6 (12.9)	3.7 (11.1)	3.2 (8.6)	2.8 (8.2)	4.4 (14.0)	1.3 (4.0)	3.0 (8.8)	4.7 (12.7)	4.0 (11.4)
	UNAIDS	4.1 (11.5)	4.3 (12.8)	3.9 (10.5)	3.1 (9.2)	3.6 (11.5)	4.1 (12.8)	4.5 (12.8)	4.7 (12.7)	2.5 (7.2)
	Regional DB	0.1 (0.4)	0.1 (0.3)	0.0 (0.1)	0.2 (0.6)	0.2 (0.7)	0.1 (0.3)	0.1 (0.2)	0.2 (0.6)	0.1 (0.3)
	Others	4.0 (11.3)	2.9 (8.4)	5.3 (14.5)	3.2 (9.3)	1.9 (6.0)	4.0 (12.5)	2.8 (8.1)	2.4 (6.3)	4.9 (13.7)
Netherlands	Global Fund	85.8 (25.7)	48.4 (19.0)	81.0 (23.8)	89.4 (29.3)	65.7 (22.3)	64.8 (18.5)	66.7 (20.1)	64.2 (17.2)	72.0 (22.2)
	World Bank	47.4 (14.2)	22.1 (8.7)	21.0 (6.2)	9.4 (3.1)	37.6 (12.8)	54.6 (15.6)	3.6 (1.1)	73.1 (19.6)	63.7 (19.6)
	WHO	25.5 (7.6)	15.5 (6.1)	22.2 (6.5)	16.8 (5.5)	15.5 (5.3)	20.0 (5.7)	19.5 (5.9)	22.2 (6.0)	20.7 (6.4)
	Gavi	22.7 (6.8)	12.6 (4.9)	28.0 (8.2)	36.1 (11.8)	36.0 (12.2)	38.9 (11.1)	73.0 (22.1)	56.1 (15.0)	55.1 (17.0)
	UNFPA	88.8 (26.6)	88.5 (34.7)	95.5 (28.1)	80.4 (26.3)	62.9 (21.4)	72.2 (20.6)	66.1 (20.0)	59.9 (16.1)	51.4 (15.8)
	EU Institutions	22.5 (6.7)	17.3 (6.8)	21.6 (6.4)	20.1 (6.6)	21.8 (7.4)	23.6 (6.8)	30.5 (9.2)	17.3 (4.6)	23.3 (7.2)
	UNICEF	11.2 (3.4)	18.9 (7.4)	32.8 (9.6)	22.6 (7.4)	21.8 (7.4)	33.3 (9.5)	21.1 (6.4)	28.0 (7.5)	19.3 (5.9)
	UNAIDS	21.1 (6.3)	17.9 (7.0)	16.1 (4.7)	10.8 (3.5)	15.6 (5.3)	18.2 (5.2)	16.2 (4.9)	21.2 (5.7)	1.2 (0.4)
	Regional DB	0.1 (0.0)	0.0 (0.0)	0.7 (0.2)	0.7 (0.2)	0.4 (0.1)	0.1 (0.0)	0.2 (0.1)	0.2 (0.1)	0.1 (0.0)
	Others	9.1 (2.7)	13.7 (5.4)	21.1 (6.3)	19.0 (6.3)	17.1 (5.9)	23.7 (6.8)	34.1 (10.3)	30.3 (8.2)	17.8 (5.5)
New Zealand	Global Fund	0.0 (0.0)	0.0 (0.0)	0.0 (0.0)	0.0 (0.0)	0.0 (0.0)	0.0 (0.0)	0.3 (4.2)	0.3 (2.8)	0.3 (4.9)
	World Bank	1.5 (7.9)	0.2 (1.2)	2.5 (19.2)	0.9 (7.1)	1.5 (14.0)	1.1 (12.5)	1.8 (22.9)	1.1 (9.5)	1.1 (16.6)
	WHO	3.3 (16.9)	3.8 (22.1)	0.9 (6.8)	3.6 (27.4)	2.2 (20.5)	2.3 (24.9)	1.5 (19.0)	3.5 (29.6)	1.2 (17.3)
	Gavi	0.0 (0.0)	0.0 (0.0)	0.0 (0.0)	0.0 (0.0)	0.0 (0.0)	0.0 (0.0)	0.0 (0.0)	0.0 (0.0)	0.0 (0.0)
	UNFPA	4.9 (25.2)	5.3 (31.0)	4.8 (36.9)	5.4 (40.5)	3.9 (36.9)	3.3 (35.8)	2.8 (34.2)	2.7 (22.6)	1.1 (16.0)
	EU Institutions	0.0 (0.0)	0.0 (0.0)	0.0 (0.0)	0.0 (0.0)	0.0 (0.0)	0.0 (0.0)	0.0 (0.0)	0.0 (0.0)	0.0 (0.0)
	UNICEF	2.4 (12.1)	2.0 (12.0)	2.5 (19.2)	1.8 (13.6)	1.7 (16.5)	1.0 (11.1)	0.7 (8.2)	2.1 (17.5)	2.2 (33.7)
	UNAIDS	1.8 (9.3)	2.0 (11.6)	0.7 (5.7)	0.5 (3.6)	0.7 (6.6)	0.8 (8.8)	0.5 (6.4)	0.6 (5.2)	0.1 (1.1)
	Regional DB	2.2 (11.2)	0.6 (3.3)	0.2 (1.6)	0.2 (1.2)	0.1 (1.3)	0.1 (1.5)	0.0 (0.0)	0.3 (2.2)	0.1 (1.4)
	Others	3.4 (17.4)	3.2 (18.9)	1.4 (10.7)	0.9 (6.5)	0.4 (4.1)	0.5 (5.4)	0.4 (5.0)	1.3 (10.7)	0.6 (9.1)
Norway	Global Fund	56.9 (21.8)	55.0 (19.0)	53.6 (15.2)	59.4 (15.3)	73.3 (18.3)	74.5 (18.2)	71.7 (16.7)	79.1 (18.5)	79.6 (18.7)
	World Bank	12.8 (4.9)	11.1 (3.8)	31.6 (8.9)	39.3 (10.1)	55.0 (13.7)	85.3 (20.8)	97.1 (22.6)	95.9 (22.4)	95.7 (22.5)
	WHO	43.6 (16.7)	45.6 (15.8)	35.2 (9.9)	34.7 (8.9)	33.6 (8.4)	29.7 (7.2)	27.6 (6.4)	27.9 (6.5)	37.3 (8.8)
	Gavi	49.5 (18.9)	69.3 (24.0)	82.9 (23.4)	122.6 (31.5)	138.2 (34.5)	154.7 (37.7)	168.1 (39.1)	146.4 (34.2)	157.0 (36.9)
	UNFPA	45.9 (17.6)	46.3 (16.0)	81.9 (23.1)	93.0 (23.9)	57.0 (14.2)	33.7 (8.2)	34.1 (7.9)	42.7 (10.0)	30.3 (7.1)

	EU Institutions	0.0 (0.0)	0.0 (0.0)	0.0 (0.0)	0.0 (0.0)	0.0 (0.0)	0.0 (0.0)	0.0 (0.0)	0.0 (0.0)	0.0 (0.0)
	UNICEF	25.8 (9.9)	27.4 (9.5)	35.8 (10.1)	14.4 (3.7)	11.1 (2.8)	10.5 (2.6)	9.6 (2.2)	12.1 (2.8)	10.3 (2.4)
	UNAIDS	14.0 (5.4)	15.1 (5.2)	14.8 (4.2)	9.4 (2.4)	14.0 (3.5)	13.3 (3.2)	11.8 (2.8)	13.6 (3.2)	1.7 (0.4)
	Regional DB	1.3 (0.5)	2.1 (0.7)	1.6 (0.5)	5.4 (1.4)	5.4 (1.3)	1.5 (0.4)	1.3 (0.3)	1.2 (0.3)	0.9 (0.2)
	Others	11.7 (4.5)	16.9 (5.9)	16.8 (4.8)	10.6 (2.7)	13.5 (3.3)	7.2 (1.8)	8.2 (1.9)	8.9 (2.1)	12.8 (3.1)
Poland	Global Fund	NA	NA	0.0 (0.0)	0.0 (0.0)	0.0 (0.0)	0.0 (0.0)	0.0 (0.0)	0.0 (0.0)	0.0 (0.0)
	World Bank	NA	NA	0.3 (2.0)	0.2 (1.8)	2.1 (12.7)	2.8 (12.2)	0.7 (3.1)	0.0 (0.2)	2.3 (10.0)
	WHO	NA	NA	2.5 (17.6)	2.7 (20.3)	2.9 (17.6)	3.2 (13.7)	2.7 (11.3)	4.4 (25.5)	3.1 (13.5)
	Gavi	NA	NA	0.0 (0.0)	0.0 (0.0)	0.0 (0.0)	0.0 (0.0)	0.0 (0.0)	0.0 (0.0)	0.0 (0.0)
	UNFPA	NA	NA	0.0 (0.3)	0.0 (0.3)	0.0 (0.2)	0.0 (0.2)	0.1 (0.4)	0.1 (0.4)	0.1 (0.2)
	EU Institutions	NA	NA	10.8 (76.6)	10.2 (77.3)	11.5 (69.3)	17.2 (73.7)	20.0 (84.4)	12.4 (72.5)	17.2 (75.0)
	UNICEF	NA	NA	0.3 (2.0)	0.0 (0.0)	0.0 (0.0)	0.0 (0.0)	0.0 (0.0)	0.0 (0.0)	0.0 (0.0)
	UNAIDS	NA	NA	0.1 (0.6)	0.0 (0.3)	0.0 (0.3)	0.0 (0.1)	0.0 (0.2)	0.0 (0.2)	0.0 (0.2)
	Regional DB	NA	NA	0.0 (0.0)	0.0 (0.0)	0.0 (0.0)	0.0 (0.0)	0.0 (0.0)	0.0 (0.0)	0.0 (0.0)
	Others	NA	NA	0.1 (0.8)	0.0 (0.0)	0.0 (0.0)	0.0 (0.1)	0.2 (0.6)	0.2 (1.3)	0.3 (1.0)
Portugal	Global Fund	1.1 (9.4)	0.0 (0.0)	0.1 (0.6)	0.0 (0.0)	0.1 (0.6)	0.1 (0.6)	0.1 (0.5)	0.1 (1.1)	0.3 (2.8)
	World Bank	1.9 (16.3)	0.2 (2.9)	0.2 (1.5)	0.1 (2.3)	0.2 (1.7)	0.2 (1.8)	3.4 (23.3)	1.2 (13.7)	1.4 (13.0)
	WHO	1.6 (14.3)	0.0 (0.0)	4.4 (43.1)	0.1 (1.1)	1.4 (14.4)	1.6 (16.5)	1.2 (8.1)	1.2 (13.9)	1.1 (10.5)
	Gavi	0.0 (0.0)	0.0 (0.0)	0.0 (0.0)	0.0 (0.0)	0.0 (0.0)	0.0 (0.0)	0.0 (0.0)	0.0 (0.0)	0.0 (0.0)
	UNFPA	0.5 (4.3)	0.2 (3.5)	0.1 (0.6)	0.1 (1.1)	0.1 (1.2)	0.1 (0.6)	0.2 (1.5)	0.1 (1.5)	0.0 (0.4)
	EU Institutions	5.9 (51.7)	4.5 (84.7)	5.3 (52.4)	5.2 (93.2)	5.8 (57.9)	7.6 (77.0)	9.3 (63.9)	5.4 (60.7)	7.5 (69.0)
	UNICEF	0.0 (0.0)	0.0 (0.5)	0.0 (0.0)	0.0 (0.1)	0.0 (0.0)	0.0 (0.1)	0.0 (0.1)	0.0 (0.0)	0.0 (0.1)
	UNAIDS	0.1 (0.7)	0.1 (1.7)	0.1 (0.8)	0.1 (1.0)	0.1 (0.8)	0.1 (0.9)	0.1 (0.6)	0.1 (1.2)	0.0 (0.1)
	Regional DB	0.3 (3.0)	0.3 (5.3)	0.0 (0.3)	0.1 (1.2)	0.1 (1.0)	0.2 (2.4)	0.3 (1.7)	0.7 (7.5)	0.2 (2.0)
	Others	0.0 (0.2)	0.0 (1.3)	0.1 (0.6)	0.0 (0.1)	2.2 (22.4)	0.0 (0.1)	0.0 (0.2)	0.0 (0.3)	0.2 (2.1)
Slovak Republic	Global Fund	NA	NA	0.0 (0.0)	0.0 (0.0)	0.0 (0.0)	0.0 (0.0)	0.0 (0.0)	0.0 (0.0)	0.0 (0.0)
	World Bank	NA	NA	0.1 (3.7)	0.1 (3.5)	0.9 (25.4)	0.0 (0.2)	0.0 (0.2)	0.5 (12.9)	0.2 (4.9)
	WHO	NA	NA	0.4 (16.9)	0.5 (20.8)	0.6 (15.6)	0.6 (16.3)	0.7 (14.1)	0.7 (19.3)	0.5 (10.8)
	Gavi	NA	NA	0.0 (0.0)	0.0 (0.0)	0.0 (0.0)	0.0 (0.0)	0.0 (0.0)	0.0 (0.0)	0.0 (0.0)
	UNFPA	NA	NA	0.0 (0.2)	0.0 (0.2)	0.0 (0.0)	0.0 (0.1)	0.0 (0.1)	0.0 (0.1)	0.0 (0.0)
	EU Institutions	NA	NA	1.9 (77.8)	1.9 (73.3)	2.1 (58.5)	3.0 (80.8)	3.8 (78.2)	2.2 (63.1)	3.1 (71.3)
	UNICEF	NA	NA	0.0 (0.5)	0.0 (0.1)	0.0 (0.0)	0.1 (2.0)	0.3 (6.5)	0.0 (0.0)	0.0 (0.0)
	UNAIDS	NA	NA	0.0 (0.0)	0.0 (0.0)	0.0 (0.0)	0.0 (0.0)	0.0 (0.0)	0.0 (0.0)	0.0 (0.0)
	Regional DB	NA	NA	0.0 (0.0)	0.0 (0.0)	0.0 (0.0)	0.0 (0.0)	0.0 (0.3)	0.0 (0.0)	0.5 (12.2)
	Others	NA	NA	0.0 (0.9)	0.1 (2.1)	0.0 (0.5)	0.0 (0.6)	0.0 (0.7)	0.1 (4.5)	0.0 (0.6)
Slovenia	Global Fund	0.0 (0.0)	0.0 (0.0)	0.0 (0.0)	0.0 (0.0)	0.0 (0.0)	0.0 (0.0)	0.0 (0.0)	0.0 (0.0)	0.0 (0.0)
	World Bank	0.3 (18.2)	0.4 (22.7)	0.3 (17.6)	0.2 (10.3)	0.3 (15.4)	0.4 (14.4)	0.3 (10.8)	0.2 (11.8)	0.2 (9.4)
	WHO	0.3 (16.1)	0.4 (25.7)	0.3 (17.8)	0.3 (18.9)	0.3 (17.8)	0.3 (12.8)	0.3 (10.6)	0.3 (14.5)	0.2 (10.0)
	Gavi	0.0 (0.0)	0.0 (0.0)	0.0 (0.0)	0.0 (0.0)	0.0 (0.0)	0.0 (0.0)	0.0 (0.0)	0.0 (0.0)	0.0 (0.0)
	UNFPA	0.0 (0.0)	0.0 (0.0)	0.0 (0.0)	0.0 (0.0)	0.0 (0.0)	0.0 (0.0)	0.0 (0.3)	0.0 (1.8)	0.0 (1.3)
	EU Institutions	1.2 (64.8)	0.9 (51.6)	1.1 (63.6)	1.1 (70.1)	1.3 (66.5)	1.8 (72.3)	2.2 (78.0)	1.2 (70.8)	1.7 (78.2)
	UNICEF	0.0 (0.3)	0.0 (0.0)	0.0 (0.2)	0.0 (0.3)	0.0 (0.0)	0.0 (0.2)	0.0 (0.2)	0.0 (0.2)	0.0 (0.2)
	UNAIDS	0.0 (0.0)	0.0 (0.0)	0.0 (0.0)	0.0 (0.0)	0.0 (0.0)	0.0 (0.0)	0.0 (0.0)	0.0 (0.0)	0.0 (0.0)
	Regional DB	0.0 (0.0)	0.0 (0.0)	0.0 (0.2)	0.0 (0.3)	0.0 (0.3)	0.0 (0.2)	0.0 (0.0)	0.0 (0.0)	0.0 (0.0)
	Others	0.0 (0.6)	0.0 (0.0)	0.0 (0.5)	0.0 (0.0)	0.0 (0.0)	0.0 (0.0)	0.0 (0.1)	0.0 (0.9)	0.0 (0.9)
South Korea	Global Fund	2.2 (5.7)	1.1 (2.8)	4.1 (7.4)	4.0 (7.1)	3.9 (5.1)	3.8 (5.5)	1.9 (2.5)	3.5 (4.5)	5.0 (6.9)
	World Bank	12.9 (33.7)	14.7 (36.8)	15.3 (27.8)	13.0 (22.9)	17.3 (22.9)	16.8 (24.0)	18.9 (24.1)	18.6 (24.0)	17.1 (23.5)

	WHO	14.6 (38.0)	17.1 (43.0)	17.6 (32.1)	16.0 (28.2)	15.7 (20.8)	19.7 (28.0)	22.7 (28.9)	25.5 (32.8)	23.9 (32.9)
	Gavi	0.3 (0.8)	0.3 (0.7)	0.9 (1.7)	0.9 (1.6)	3.8 (5.0)	3.7 (5.3)	3.6 (4.6)	3.5 (4.5)	4.5 (6.1)
	UNFPA	0.1 (0.3)	0.8 (2.1)	3.0 (5.4)	1.5 (2.6)	4.2 (5.6)	7.9 (11.3)	7.0 (8.9)	5.3 (6.8)	2.8 (3.9)
	EU Institutions	0.0 (0.0)	0.0 (0.0)	0.0 (0.0)	0.0 (0.0)	0.0 (0.0)	0.0 (0.0)	0.0 (0.0)	0.0 (0.0)	0.0 (0.0)
	UNICEF	3.0 (7.8)	0.5 (1.2)	9.9 (18.1)	17.4 (30.6)	26.8 (35.5)	16.3 (23.2)	16.8 (21.3)	11.3 (14.6)	8.7 (11.9)
	UNAIDS	0.1 (0.3)	0.1 (0.3)	0.0 (0.0)	0.0 (0.0)	0.0 (0.0)	0.0 (0.0)	1.3 (1.6)	0.0 (0.0)	0.0 (0.0)
	Regional DB	4.7 (12.2)	4.4 (11.1)	2.8 (5.1)	3.7 (6.6)	3.5 (4.6)	1.8 (2.5)	4.7 (6.0)	3.3 (4.2)	5.8 (8.0)
	Others	0.5 (1.2)	0.7 (1.8)	1.3 (2.4)	0.2 (0.4)	0.2 (0.3)	0.2 (0.2)	1.6 (2.1)	6.6 (8.6)	4.9 (6.8)
Spain	Global Fund	0.0 (0.0)	0.0 (0.0)	0.0 (0.0)	0.0 (0.0)	0.0 (0.0)	0.0 (0.0)	0.0 (0.0)	0.0 (0.0)	0.0 (0.0)
	World Bank	24.9 (19.9)	0.0 (0.0)	4.1 (6.4)	1.6 (2.8)	2.3 (4.2)	27.3 (30.3)	33.9 (31.0)	26.5 (35.4)	21.1 (25.5)
	WHO	13.0 (10.4)	9.7 (20.7)	21.3 (33.3)	9.8 (17.9)	11.1 (20.6)	11.5 (12.7)	12.1 (11.1)	8.9 (11.9)	9.5 (11.5)
	Gavi	0.0 (0.0)	0.0 (0.0)	0.0 (0.0)	0.0 (0.0)	0.0 (0.0)	0.0 (0.0)	0.0 (0.0)	0.0 (0.0)	0.0 (0.0)
	UNFPA	14.2 (11.4)	1.8 (3.8)	1.8 (2.7)	1.8 (3.2)	0.2 (0.5)	0.6 (0.6)	0.4 (0.3)	0.4 (0.5)	0.4 (0.5)
	EU Institutions	43.7 (34.9)	27.9 (59.4)	33.6 (52.5)	31.0 (56.8)	35.6 (66.2)	47.3 (52.6)	59.0 (54.0)	34.1 (45.4)	47.6 (57.6)
	UNICEF	5.5 (4.4)	0.5 (1.0)	0.0 (0.0)	3.3 (6.1)	2.5 (4.6)	0.0 (0.0)	0.2 (0.1)	0.1 (0.1)	1.1 (1.3)
	UNAIDS	3.2 (2.6)	0.0 (0.0)	0.6 (0.9)	0.0 (0.0)	0.0 (0.0)	0.0 (0.0)	0.0 (0.0)	0.0 (0.0)	0.0 (0.0)
	Regional DB	9.3 (7.4)	6.4 (13.6)	0.2 (0.3)	6.7 (12.3)	0.4 (0.7)	2.8 (3.1)	1.7 (1.6)	1.3 (1.7)	1.5 (1.8)
	Others	11.3 (9.1)	0.7 (1.6)	2.4 (3.8)	0.4 (0.9)	1.7 (3.2)	0.6 (0.7)	2.0 (1.8)	3.8 (5.0)	1.5 (1.8)
Sweden	Global Fund	73.3 (21.5)	115.0 (32.8)	89.9 (25.1)	58.9 (17.7)	97.8 (27.0)	96.5 (26.8)	88.9 (23.1)	92.4 (24.0)	89.9 (27.2)
	World Bank	38.5 (11.3)	32.0 (9.1)	41.0 (11.4)	34.0 (10.2)	40.5 (11.2)	28.6 (7.9)	51.1 (13.3)	36.1 (9.4)	38.3 (11.6)
	WHO	16.7 (4.9)	21.0 (6.0)	19.9 (5.5)	29.0 (8.7)	30.0 (8.3)	33.0 (9.2)	34.7 (9.0)	40.9 (10.6)	20.9 (6.3)
	Gavi	69.8 (20.5)	0.0 (0.0)	52.0 (14.5)	38.6 (11.6)	37.9 (10.4)	31.0 (8.6)	30.8 (8.0)	35.2 (9.2)	30.0 (9.1)
	UNFPA	60.5 (17.7)	57.8 (16.5)	66.0 (18.4)	75.0 (22.6)	61.9 (17.1)	57.1 (15.9)	73.7 (19.2)	60.5 (15.7)	60.3 (18.3)
	EU Institutions	11.0 (3.2)	8.5 (2.4)	10.7 (3.0)	11.2 (3.4)	14.8 (4.1)	15.6 (4.3)	23.2 (6.0)	11.5 (3.0)	18.5 (5.6)
	UNICEF	21.2 (6.2)	13.1 (3.7)	12.9 (3.6)	34.0 (10.2)	25.4 (7.0)	49.2 (13.7)	28.5 (7.4)	29.1 (7.6)	28.9 (8.8)
	UNAIDS	23.6 (6.9)	24.9 (7.1)	19.6 (5.5)	12.1 (3.6)	14.9 (4.1)	21.7 (6.0)	22.0 (5.7)	31.7 (8.2)	3.7 (1.1)
	Regional DB	4.4 (1.3)	4.6 (1.3)	5.6 (1.6)	9.1 (2.7)	5.8 (1.6)	6.2 (1.7)	1.9 (0.5)	1.5 (0.4)	1.5 (0.4)
	Others	21.9 (6.5)	73.9 (21.1)	40.9 (11.5)	30.4 (9.1)	33.7 (9.2)	21.2 (5.9)	29.2 (7.6)	45.7 (11.9)	38.2 (11.5)
Switzerland	Global Fund	7.9 (10.0)	7.9 (9.9)	9.9 (13.2)	20.0 (18.9)	20.1 (18.7)	20.2 (19.5)	20.3 (18.2)	18.8 (17.9)	18.6 (19.3)
	World Bank	27.7 (34.8)	18.8 (23.5)	23.6 (31.4)	18.1 (17.1)	27.6 (25.8)	28.1 (27.1)	32.3 (28.9)	28.2 (26.9)	25.4 (26.4)
	WHO	11.3 (14.2)	9.5 (11.8)	12.7 (16.9)	16.9 (16.0)	17.2 (16.1)	13.4 (13.0)	14.5 (13.0)	13.7 (13.1)	14.1 (14.6)
	Gavi	0.0 (0.0)	0.0 (0.0)	0.0 (0.0)	0.0 (0.0)	0.0 (0.0)	1.6 (1.6)	0.0 (0.0)	0.0 (0.0)	0.0 (0.0)
	UNFPA	13.9 (17.5)	25.5 (31.9)	6.5 (8.7)	18.0 (17.0)	13.5 (12.6)	10.6 (10.2)	12.3 (11.0)	10.3 (9.8)	9.5 (9.9)
	EU Institutions	0.0 (0.0)	0.0 (0.0)	0.0 (0.0)	0.0 (0.0)	0.0 (0.0)	0.0 (0.0)	0.0 (0.0)	0.0 (0.0)	0.0 (0.0)
	UNICEF	5.1 (6.4)	3.9 (4.8)	7.4 (9.9)	8.3 (7.8)	8.3 (7.8)	9.3 (9.0)	8.4 (7.5)	8.6 (8.2)	7.0 (7.3)
	UNAIDS	3.4 (4.2)	3.6 (4.5)	6.8 (9.0)	4.5 (4.2)	7.5 (7.0)	9.5 (9.1)	8.3 (7.4)	9.4 (8.9)	1.2 (1.2)
	Regional DB	3.1 (3.9)	1.9 (2.3)	1.4 (1.8)	4.2 (4.0)	4.6 (4.3)	1.5 (1.5)	1.6 (1.4)	1.5 (1.4)	5.5 (5.7)
	Others	7.2 (9.1)	8.9 (11.3)	6.8 (9.0)	15.7 (14.9)	8.3 (7.8)	9.4 (9.1)	14.1 (12.6)	14.4 (13.8)	15.1 (15.5)
United Kingdom	Global Fund	260.7 (22.2)	230.2 (21.1)	768.2 (35.6)	396.0 (23.2)	138.0 (11.2)	206.9 (17.2)	420.9 (26.3)	469.0 (27.6)	472.2 (28.6)
	World Bank	260.8 (22.2)	172.3 (15.8)	354.4 (16.4)	278.1 (16.3)	214.5 (17.4)	163.8 (13.6)	229.3 (14.3)	321.7 (18.9)	147.4 (8.9)
	WHO	257.8 (22.0)	210.3 (19.2)	241.4 (11.2)	146.5 (8.6)	139.1 (11.3)	109.1 (9.1)	160.1 (10.0)	212.1 (12.5)	212.8 (12.9)
	Gavi	67.0 (5.7)	173.8 (15.9)	428.8 (19.8)	350.5 (20.5)	348.3 (28.2)	265.4 (22.1)	257.2 (16.1)	245.8 (14.4)	241.0 (14.6)
	UNFPA	142.5 (12.1)	167.0 (15.3)	58.7 (2.7)	298.1 (17.4)	126.9 (10.3)	110.5 (9.2)	133.5 (8.3)	172.1 (10.1)	230.1 (14.0)
	EU Institutions	63.7 (5.4)	50.5 (4.6)	63.5 (2.9)	54.3 (3.2)	68.9 (5.6)	90.7 (7.6)	149.4 (9.3)	70.1 (4.1)	97.8 (5.9)
	UNICEF	42.9 (3.7)	9.0 (0.8)	110.7 (5.1)	92.0 (5.4)	101.6 (8.2)	136.7 (11.4)	155.5 (9.7)	136.1 (8.0)	141.5 (8.6)
	UNAIDS	11.9 (1.0)	11.0 (1.0)	14.2 (0.7)	9.3 (0.5)	13.4 (1.1)	15.5 (1.3)	15.2 (0.9)	18.0 (1.1)	2.1 (0.1)
	Regional DB	17.4 (1.5)	11.2 (1.0)	8.0 (0.4)	25.5 (1.5)	26.3 (2.1)	6.3 (0.5)	2.0 (0.1)	4.2 (0.2)	2.6 (0.2)

	Others	48.8 (4.2)	58.1 (5.3)	112.2 (5.3)	60.3 (3.6)	59.2 (4.7)	95.6 (8.0)	77.2 (4.8)	52.2 (3.0)	102.1 (6.1)
United States	Global Fund	1087.8 (59.2)	1355.0 (63.1)	1375.7 (62.3)	1755.9 (66.1)	576.8 (36.7)	1931.5 (67.9)	1076.9 (48.9)	976.4 (52.3)	1040.0 (48.6)
	World Bank	140.7 (7.7)	174.4 (8.1)	166.3 (7.5)	182.2 (6.9)	151.4 (9.6)	146.5 (5.2)	255.3 (11.6)	111.5 (6.0)	163.7 (7.7)
	WHO	233.8 (12.7)	187.5 (8.7)	219.8 (10.0)	256.7 (9.7)	226.3 (14.4)	240.8 (8.5)	269.1 (12.2)	204.3 (10.9)	190.6 (8.9)
	Gavi	94.0 (5.1)	136.6 (6.4)	141.3 (6.4)	177.6 (6.7)	201.6 (12.8)	227.5 (8.0)	264.8 (12.0)	273.0 (14.6)	273.8 (12.8)
	UNFPA	42.4 (2.3)	33.9 (1.6)	31.6 (1.4)	33.9 (1.3)	22.7 (1.4)	23.7 (0.8)	0.3 (0.0)	0.0 (0.0)	0.0 (0.0)
	EU Institutions	0.0 (0.0)	0.0 (0.0)	0.0 (0.0)	0.0 (0.0)	0.0 (0.0)	0.0 (0.0)	0.0 (0.0)	0.0 (0.0)	0.0 (0.0)
	UNICEF	66.7 (3.6)	73.0 (3.4)	97.2 (4.4)	82.7 (3.1)	115.2 (7.3)	111.0 (3.9)	147.5 (6.7)	108.5 (5.8)	56.1 (2.6)
	UNAIDS	39.1 (2.1)	42.2 (2.0)	40.3 (1.8)	25.1 (0.9)	42.6 (2.7)	58.4 (2.1)	45.4 (2.1)	59.3 (3.2)	15.4 (0.7)
	Regional DB	14.9 (0.8)	23.3 (1.1)	11.2 (0.5)	22.9 (0.9)	21.3 (1.4)	10.1 (0.4)	5.4 (0.2)	4.3 (0.2)	2.4 (0.1)
	Others	117.6 (6.3)	121.1 (5.5)	125.1 (5.7)	119.8 (4.5)	213.9 (13.7)	93.0 (3.2)	135.6 (6.1)	131.1 (6.9)	396.9 (18.6)
All members	Global Fund	2530.0 (35.4)	3033.6 (41.3)	3422.9 (40.0)	3531.4 (41.1)	2296.8 (32.9)	3511.7 (42.0)	3067.3 (35.5)	2975.9 (35.3)	3168.1 (37.1)
	World Bank	1113.0 (15.6)	923.2 (12.6)	1078.4 (12.6)	893.4 (10.4)	940.0 (13.5)	990.0 (11.8)	1237.8 (14.3)	1449.4 (17.2)	1125.0 (13.2)
	WHO	940.5 (13.2)	856.5 (11.6)	910.6 (10.6)	807.3 (9.4)	768.1 (11.0)	777.7 (9.3)	861.8 (10.0)	853.1 (10.1)	782.5 (9.2)
	Gavi	425.6 (6.0)	487.7 (6.6)	874.8 (10.2)	867.9 (10.1)	841.5 (12.1)	962.1 (11.5)	1096.2 (12.7)	966.5 (11.5)	1036.4 (12.1)
	UNFPA	628.9 (8.8)	624.3 (8.5)	555.7 (6.5)	862.3 (10.0)	502.0 (7.2)	443.7 (5.3)	480.6 (5.6)	553.4 (6.6)	533.1 (6.2)
	EU Institutions	439.3 (6.1)	327.2 (4.4)	423.4 (4.9)	408.2 (4.8)	460.1 (6.6)	581.1 (6.9)	779.9 (9.0)	441.7 (5.2)	596.2 (7.0)
	UNICEF	374.1 (5.2)	340.9 (4.6)	580.3 (6.8)	492.4 (5.7)	433.7 (6.2)	521.9 (6.2)	514.1 (5.9)	468.7 (5.6)	408.4 (4.8)
	UNAIDS	161.9 (2.3)	153.7 (2.1)	149.9 (1.8)	97.6 (1.1)	139.8 (2.0)	161.1 (1.9)	149.5 (1.7)	181.6 (2.2)	31.9 (0.4)
	Regional DB	155.8 (2.2)	112.0 (1.5)	83.3 (1.0)	188.5 (2.2)	144.2 (2.1)	58.9 (0.7)	57.1 (0.7)	73.5 (0.9)	56.7 (0.7)
	Others	382.8 (5.3)	495.2 (6.8)	479.9 (5.5)	433.3 (5.1)	456.4 (6.5)	356.4 (4.2)	397.2 (4.6)	455.4 (5.5)	810.8 (9.5)

DAH: development assistance for health; DAH: Development Assistance Committee; Global Fund: The Global Fund to Fight AIDS, Tuberculosis and Malaria; WHO: World Health Organization; Gavi: Gavi, The Vaccine Alliance; UNFPA: United Nations Population Fund; EU Institutions: Institutions of the European Union; UNICEF: United Nations Children's Fund; UNAIDS: Joint United Nations Programme on HIV/AIDS; Regional DB: regional development banks

Table S5: Estimated DAH in constant prices at 2019 from all the 29 DAC member countries by target region (%), 2011–2019

Country	Target region	2011	2012	2013	2014	2015	2016	2017	2018	2019
Australia	Africa	75.0 (15.6)	109.3 (19.6)	108.3 (23.4)	99.2 (22.5)	76.1 (20.7)	98.3 (28.5)	25.5 (10.3)	17.9 (5.5)	51.2 (16.7)
	Asia	190.8 (39.7)	255.6 (45.7)	164.4 (35.6)	168.4 (38.2)	100.7 (27.4)	87.5 (25.4)	68.3 (27.5)	62.2 (19.1)	61.0 (19.9)
	Europe	1.7 (0.4)	3.8 (0.7)	3.8 (0.8)	2.1 (0.5)	3.2 (0.9)	2.7 (0.8)	2.2 (0.9)	0.6 (0.2)	0.5 (0.2)
	America	7.1 (1.5)	8.1 (1.5)	7.7 (1.7)	4.4 (1.0)	5.6 (1.5)	5.2 (1.5)	3.2 (1.3)	1.5 (0.5)	1.8 (0.6)
	Oceania	148.5 (30.9)	127.3 (22.8)	140.5 (30.4)	121.1 (27.5)	130.0 (35.4)	86.8 (25.2)	97.2 (39.0)	112.4 (34.6)	128.8 (42.0)
	Regional and Unspecified	57.5 (12.0)	54.7 (9.8)	37.1 (8.0)	45.4 (10.3)	52.1 (14.2)	64.5 (18.7)	52.5 (21.1)	130.8 (40.2)	63.1 (20.6)
Austria	Africa	19.3 (41.4)	15.1 (38.5)	17.8 (46.1)	22.9 (54.2)	22.7 (58.8)	24.6 (55.1)	18.9 (39.5)	20.2 (53.1)	21.9 (48.1)
	Asia	17.2 (36.8)	14.1 (36.1)	14.1 (36.6)	10.5 (24.9)	12.0 (31.2)	13.2 (29.5)	20.1 (42.0)	11.2 (29.5)	15.9 (34.8)
	Europe	3.7 (7.9)	4.3 (11.0)	1.6 (4.2)	3.3 (7.8)	0.8 (2.2)	2.8 (6.4)	4.8 (10.0)	2.4 (6.2)	3.0 (6.6)
	America	4.0 (8.5)	3.2 (8.1)	2.6 (6.8)	3.6 (8.6)	1.5 (3.8)	2.0 (4.4)	1.3 (2.8)	1.7 (4.5)	1.2 (2.6)
	Oceania	0.2 (0.3)	0.2 (0.5)	0.2 (0.4)	0.1 (0.2)	0.2 (0.5)	0.1 (0.2)	0.1 (0.2)	0.1 (0.1)	0.2 (0.4)
	Regional and Unspecified	2.4 (5.0)	2.2 (5.8)	2.3 (5.9)	1.8 (4.3)	1.4 (3.6)	1.9 (4.3)	2.6 (5.5)	2.5 (6.5)	3.4 (7.6)
Belgium	Africa	143.5 (58.4)	135.0 (59.3)	143.5 (63.8)	156.4 (64.9)	132.2 (62.9)	121.1 (59.5)	128.5 (60.7)	115.4 (62.3)	119.6 (64.8)
	Asia	42.7 (17.4)	39.2 (17.2)	32.6 (14.5)	30.2 (12.5)	25.7 (12.2)	25.3 (12.4)	24.8 (11.7)	23.7 (12.8)	22.5 (12.2)
	Europe	3.4 (1.4)	2.7 (1.2)	1.6 (0.7)	2.2 (0.9)	1.9 (0.9)	4.1 (2.0)	9.1 (4.3)	3.1 (1.7)	4.3 (2.4)
	America	20.6 (8.4)	17.0 (7.5)	14.0 (6.2)	23.9 (9.9)	19.7 (9.4)	19.7 (9.7)	10.4 (4.9)	9.0 (4.9)	5.2 (2.8)
	Oceania	0.6 (0.2)	0.8 (0.3)	0.4 (0.2)	0.5 (0.2)	0.6 (0.3)	0.5 (0.3)	0.5 (0.2)	0.3 (0.2)	0.4 (0.2)
	Regional and Unspecified	34.8 (14.2)	33.0 (14.5)	32.9 (14.6)	27.9 (11.6)	30.0 (14.3)	33.0 (16.2)	38.4 (18.1)	33.6 (18.1)	32.4 (17.6)
Canada	Africa	486.2 (59.8)	526.8 (65.4)	546.4 (63.0)	393.1 (64.6)	565.1 (65.3)	453.2 (57.7)	567.8 (64.6)	550.4 (61.4)	530.5 (62.8)
	Asia	183.9 (22.6)	153.0 (19.0)	208.2 (24.0)	125.4 (20.6)	208.3 (24.1)	225.5 (28.7)	212.9 (24.2)	229.8 (25.6)	215.8 (25.6)
	Europe	15.7 (1.9)	11.5 (1.4)	6.6 (0.8)	3.8 (0.6)	9.3 (1.1)	5.7 (0.7)	5.9 (0.7)	7.1 (0.8)	6.0 (0.7)
	America	95.9 (11.8)	71.8 (8.9)	52.8 (6.1)	36.5 (6.0)	44.0 (5.1)	59.7 (7.6)	68.1 (7.8)	83.5 (9.3)	64.9 (7.7)
	Oceania	1.9 (0.2)	2.9 (0.4)	3.0 (0.3)	0.4 (0.1)	3.5 (0.4)	1.9 (0.2)	2.8 (0.3)	2.9 (0.3)	3.5 (0.4)
	Regional and Unspecified	29.3 (3.6)	40.1 (5.0)	50.9 (5.9)	49.3 (8.1)	34.9 (4.0)	39.7 (5.0)	21.3 (2.4)	23.4 (2.6)	23.5 (2.8)
Czech Republic	Africa	4.4 (43.9)	3.1 (37.7)	3.8 (39.3)	4.0 (47.3)	4.8 (48.9)	5.3 (41.5)	5.3 (31.7)	3.6 (43.6)	4.9 (38.0)
	Asia	3.5 (35.0)	3.0 (35.5)	3.3 (33.9)	2.8 (32.8)	3.2 (33.0)	4.5 (35.4)	6.7 (40.3)	2.5 (30.3)	4.7 (37.1)
	Europe	1.0 (10.0)	1.0 (11.9)	0.9 (9.1)	1.1 (13.4)	0.8 (8.3)	1.6 (12.5)	2.7 (16.2)	0.9 (10.8)	1.3 (10.5)
	America	0.3 (2.9)	0.4 (5.2)	0.5 (5.0)	0.1 (1.6)	0.2 (2.5)	0.3 (2.3)	0.3 (2.0)	0.2 (2.8)	0.3 (2.4)
	Oceania	0.0 (0.3)	0.0 (0.4)	0.0 (0.5)	0.0 (0.0)	0.0 (0.3)	0.0 (0.3)	0.1 (0.3)	0.0 (0.2)	0.0 (0.3)
	Regional and Unspecified	0.8 (7.9)	0.8 (9.4)	1.2 (12.3)	0.4 (4.9)	0.7 (7.0)	1.0 (8.0)	1.6 (9.5)	1.0 (12.3)	1.5 (11.7)
Denmark	Africa	152.3 (64.4)	133.1 (63.3)	106.9 (57.0)	121.2 (55.0)	92.6 (57.3)	100.8 (69.2)	79.1 (44.8)	84.3 (42.1)	91.5 (53.9)
	Asia	43.7 (18.5)	44.6 (21.2)	36.5 (19.5)	41.4 (18.8)	20.8 (12.8)	19.6 (13.4)	19.9 (11.3)	25.1 (12.5)	22.3 (13.1)
	Europe	3.0 (1.3)	2.4 (1.1)	1.9 (1.0)	2.4 (1.1)	1.3 (0.8)	2.3 (1.5)	4.3 (2.4)	2.2 (1.1)	2.9 (1.7)
	America	7.8 (3.3)	7.5 (3.6)	6.8 (3.6)	8.2 (3.7)	3.5 (2.2)	3.6 (2.5)	3.7 (2.1)	3.6 (1.8)	3.4 (2.0)
	Oceania	0.7 (0.3)	0.8 (0.4)	0.7 (0.4)	0.7 (0.3)	0.4 (0.3)	0.4 (0.3)	0.4 (0.2)	0.5 (0.2)	0.5 (0.3)
	Regional and Unspecified	28.8 (12.2)	22.0 (10.5)	34.6 (18.5)	46.4 (21.1)	43.0 (26.6)	19.1 (13.1)	69.0 (39.1)	84.3 (42.2)	49.3 (29.0)
Finland	Africa	43.3 (40.7)	41.8 (39.3)	45.4 (43.0)	70.8 (50.2)	46.4 (49.9)	25.9 (49.1)	27.5 (49.1)	25.1 (50.7)	24.5 (52.1)
	Asia	30.5 (28.7)	27.4 (25.8)	31.3 (29.6)	42.3 (29.9)	28.0 (30.1)	16.8 (31.9)	17.1 (30.5)	12.7 (25.6)	11.6 (24.7)
	Europe	1.9 (1.8)	1.5 (1.5)	1.6 (1.5)	1.7 (1.2)	1.4 (1.5)	1.5 (2.9)	3.6 (6.4)	3.7 (7.5)	3.5 (7.5)
	America	12.6 (11.9)	12.1 (11.4)	7.5 (7.1)	7.0 (4.9)	4.6 (5.0)	2.4 (4.5)	1.6 (2.9)	1.5 (3.1)	1.3 (2.9)
	Oceania	0.4 (0.4)	0.5 (0.5)	0.6 (0.5)	0.6 (0.4)	0.5 (0.5)	0.2 (0.3)	0.2 (0.4)	0.2 (0.4)	0.2 (0.4)
	Regional and Unspecified	17.5 (16.5)	22.9 (21.5)	19.3 (18.3)	18.8 (13.3)	12.0 (13.0)	5.9 (11.3)	6.0 (10.7)	6.3 (12.7)	5.8 (12.4)
France	Africa	440.9 (58.0)	458.6 (60.7)	448.9 (53.2)	532.0 (49.5)	505.4 (67.8)	576.5 (67.9)	499.7 (60.6)	595.0 (67.9)	572.0 (62.6)
	Asia	201.0 (26.4)	175.6 (23.2)	199.7 (23.7)	167.5 (15.6)	155.8 (20.9)	164.4 (19.3)	163.9 (19.9)	156.2 (17.8)	204.4 (22.4)

	Europe	20.2 (2.7)	19.5 (2.6)	14.8 (1.8)	17.4 (1.6)	11.7 (1.6)	21.6 (2.5)	37.6 (4.6)	19.2 (2.2)	25.5 (2.8)
	America	47.6 (6.3)	41.0 (5.4)	125.5 (14.9)	315.3 (29.3)	27.3 (3.7)	28.1 (3.3)	33.8 (4.1)	41.6 (4.7)	32.0 (3.5)
	Oceania	3.5 (0.5)	7.7 (1.0)	5.5 (0.6)	4.3 (0.4)	4.0 (0.5)	10.2 (1.2)	5.5 (0.7)	5.2 (0.6)	8.9 (1.0)
	Regional and Unspecified	47.4 (6.2)	53.1 (7.0)	48.8 (5.8)	37.8 (3.5)	41.0 (5.5)	48.7 (5.7)	83.6 (10.2)	58.5 (6.7)	70.1 (7.7)
Germany	Africa	429.7 (46.8)	446.6 (50.6)	450.8 (47.8)	519.5 (52.6)	470.7 (46.9)	588.5 (50.1)	609.6 (46.1)	621.2 (45.5)	678.7 (48.0)
	Asia	369.2 (40.3)	300.4 (34.0)	372.1 (39.5)	347.5 (35.2)	426.2 (42.5)	445.6 (37.9)	453.4 (34.3)	392.8 (28.8)	389.6 (27.5)
	Europe	22.5 (2.5)	21.9 (2.5)	15.9 (1.7)	18.0 (1.8)	13.2 (1.3)	27.4 (2.3)	62.4 (4.7)	33.5 (2.5)	30.8 (2.2)
	America	40.9 (4.5)	35.9 (4.1)	39.8 (4.2)	36.4 (3.7)	25.5 (2.5)	24.5 (2.1)	26.7 (2.0)	23.6 (1.7)	24.1 (1.7)
	Oceania	3.0 (0.3)	5.1 (0.6)	3.8 (0.4)	3.1 (0.3)	3.3 (0.3)	3.1 (0.3)	3.1 (0.2)	3.0 (0.2)	4.1 (0.3)
	Regional and Unspecified	51.9 (5.7)	73.0 (8.3)	60.4 (6.4)	64.0 (6.5)	64.3 (6.4)	86.1 (7.3)	166.0 (12.6)	290.6 (21.3)	288.0 (20.3)
Greece	Africa	4.1 (36.6)	3.0 (38.1)	3.6 (39.1)	3.9 (51.5)	4.7 (57.1)	4.7 (46.8)	5.4 (41.6)	4.1 (49.2)	3.9 (47.3)
	Asia	4.4 (39.4)	3.1 (39.4)	2.8 (30.8)	1.6 (21.9)	1.8 (22.4)	2.5 (25.3)	3.1 (24.3)	1.8 (21.8)	1.7 (20.6)
	Europe	0.9 (7.9)	0.4 (5.3)	0.4 (4.6)	0.4 (4.7)	0.3 (3.5)	1.0 (10.6)	2.4 (18.8)	0.9 (10.5)	1.2 (14.3)
	America	0.4 (3.9)	0.5 (6.7)	0.6 (6.7)	0.3 (3.6)	0.3 (3.7)	0.3 (3.4)	0.4 (2.8)	0.3 (3.5)	0.2 (2.5)
	Oceania	0.0 (0.4)	0.0 (0.3)	0.1 (0.6)	0.0 (0.5)	0.0 (0.5)	0.0 (0.4)	0.0 (0.4)	0.0 (0.3)	0.0 (0.2)
	Regional and Unspecified	1.3 (11.8)	0.8 (10.2)	1.7 (18.2)	1.3 (17.8)	1.0 (12.7)	1.3 (13.5)	1.6 (12.2)	1.2 (14.8)	1.2 (15.1)
Hungary	Africa	NA	NA	NA	2.7 (49.9)	3.7 (50.5)	3.6 (48.8)	2.4 (38.6)	4.7 (34.0)	3.3 (25.6)
	Asia	NA	NA	NA	1.7 (32.5)	2.8 (38.4)	2.3 (30.2)	1.7 (26.6)	6.0 (43.8)	6.4 (49.5)
	Europe	NA	NA	NA	0.2 (2.9)	0.2 (2.1)	0.7 (8.8)	1.2 (18.6)	1.6 (11.4)	0.9 (6.8)
	America	NA	NA	NA	0.1 (2.8)	0.2 (2.6)	0.2 (2.8)	0.2 (3.1)	0.3 (1.8)	0.2 (1.8)
	Oceania	NA	NA	NA	0.0 (0.3)	0.0 (0.3)	0.0 (0.3)	0.0 (0.4)	0.0 (0.3)	0.0 (0.2)
	Regional and Unspecified	NA	NA	NA	0.6 (11.6)	0.5 (6.2)	0.7 (9.1)	0.8 (12.7)	1.2 (8.7)	2.1 (16.1)
Iceland	Africa	1.8 (64.7)	2.1 (72.7)	3.3 (77.8)	2.5 (78.4)	1.8 (78.1)	2.1 (79.4)	0.8 (48.6)	2.4 (74.9)	3.5 (75.1)
	Asia	0.8 (28.3)	0.6 (21.6)	0.7 (17.2)	0.5 (16.2)	0.3 (14.2)	0.4 (13.5)	0.8 (45.3)	0.6 (18.1)	0.7 (14.2)
	Europe	0.0 (0.2)	0.0 (0.3)	0.0 (0.3)	0.0 (0.2)	0.0 (0.4)	0.0 (0.4)	0.0 (0.7)	0.0 (0.2)	0.0 (0.1)
	America	0.0 (1.4)	0.0 (0.8)	0.0 (0.6)	0.0 (0.4)	0.0 (1.1)	0.0 (0.8)	0.0 (2.5)	0.0 (0.9)	0.0 (0.4)
	Oceania	0.0 (0.2)	0.0 (0.1)	0.0 (0.1)	0.0 (0.1)	0.0 (0.2)	0.0 (0.1)	0.0 (0.3)	0.0 (0.1)	0.0 (0.1)
	Regional and Unspecified	0.1 (5.3)	0.1 (4.5)	0.2 (4.1)	0.1 (4.7)	0.1 (6.0)	0.2 (5.8)	0.0 (2.6)	0.2 (5.8)	0.5 (10.1)
Ireland	Africa	93.3 (74.2)	88.0 (73.0)	99.9 (76.5)	104.3 (81.2)	82.9 (77.3)	81.6 (75.3)	79.5 (73.2)	75.1 (76.9)	80.3 (75.4)
	Asia	12.1 (9.6)	13.1 (10.9)	14.5 (11.1)	11.4 (8.9)	9.8 (9.1)	10.6 (9.8)	11.1 (10.2)	9.2 (9.5)	10.2 (9.6)
	Europe	1.5 (1.2)	1.2 (1.0)	1.1 (0.8)	0.9 (0.7)	0.7 (0.6)	1.5 (1.3)	2.8 (2.6)	1.2 (1.2)	1.7 (1.6)
	America	2.7 (2.2)	2.4 (2.0)	2.5 (1.9)	1.9 (1.5)	1.6 (1.5)	1.7 (1.5)	1.4 (1.3)	1.2 (1.2)	1.3 (1.2)
	Oceania	0.3 (0.2)	0.4 (0.3)	0.3 (0.3)	0.2 (0.2)	0.3 (0.2)	0.2 (0.2)	0.2 (0.2)	0.2 (0.2)	0.2 (0.2)
	Regional and Unspecified	15.9 (12.6)	15.5 (12.8)	12.4 (9.5)	9.6 (7.5)	12.0 (11.2)	12.9 (11.9)	13.7 (12.6)	10.9 (11.1)	12.8 (12.0)
Italy	Africa	101.6 (59.4)	68.5 (52.1)	84.5 (53.2)	127.0 (65.0)	143.4 (65.8)	143.3 (59.9)	124.1 (45.0)	180.1 (60.8)	196.5 (60.1)
	Asia	44.2 (25.9)	32.5 (24.7)	47.7 (30.0)	41.2 (21.1)	51.4 (23.6)	63.3 (26.5)	62.1 (22.5)	74.8 (25.3)	75.3 (23.0)
	Europe	9.5 (5.5)	4.8 (3.7)	2.6 (1.6)	3.8 (2.0)	4.5 (2.1)	11.7 (4.9)	22.7 (8.3)	14.5 (4.9)	14.8 (4.5)
	America	10.6 (6.2)	12.0 (9.1)	12.2 (7.7)	12.7 (6.5)	10.6 (4.9)	8.4 (3.5)	7.6 (2.7)	10.3 (3.5)	18.7 (5.7)
	Oceania	0.4 (0.2)	0.6 (0.5)	0.4 (0.3)	0.8 (0.4)	0.7 (0.3)	0.9 (0.4)	0.6 (0.2)	0.7 (0.2)	1.3 (0.4)
	Regional and Unspecified	4.7 (2.8)	13.0 (9.9)	11.5 (7.3)	10.0 (5.1)	7.4 (3.4)	11.7 (4.9)	58.5 (21.2)	15.6 (5.3)	20.6 (6.3)
Japan	Africa	275.1 (39.5)	418.0 (48.9)	382.0 (53.3)	422.0 (49.3)	460.6 (53.2)	395.3 (44.5)	536.4 (48.9)	478.4 (50.4)	513.8 (50.3)
	Asia	301.0 (43.2)	333.3 (39.0)	235.6 (32.9)	285.1 (33.3)	278.6 (32.2)	367.4 (41.3)	433.5 (39.5)	324.4 (34.2)	337.7 (33.1)
	Europe	14.0 (2.0)	13.9 (1.6)	9.6 (1.3)	32.8 (3.8)	48.8 (5.6)	15.0 (1.7)	12.7 (1.2)	12.2 (1.3)	13.5 (1.3)
	America	37.1 (5.3)	39.2 (4.6)	34.1 (4.8)	43.7 (5.1)	42.1 (4.9)	53.2 (6.0)	50.8 (4.6)	48.0 (5.1)	53.9 (5.3)
	Oceania	31.2 (4.5)	16.4 (1.9)	17.4 (2.4)	13.1 (1.5)	8.0 (0.9)	10.9 (1.2)	9.8 (0.9)	9.7 (1.0)	15.0 (1.5)
	Regional and Unspecified	38.4 (5.5)	34.9 (4.1)	38.3 (5.3)	58.8 (6.9)	28.2 (3.3)	46.9 (5.3)	53.1 (4.8)	75.8 (8.0)	87.2 (8.5)
Luxembourg	Africa	26.0 (41.2)	26.0 (41.3)	25.9 (36.3)	28.5 (42.5)	28.0 (46.3)	26.6 (42.0)	18.6 (30.3)	22.8 (37.1)	27.5 (40.2)

	Asia	19.5 (30.9)	21.2 (33.6)	24.4 (34.2)	21.8 (32.5)	21.7 (35.9)	17.5 (27.7)	20.8 (33.8)	21.5 (34.9)	15.0 (22.0)
	Europe	1.9 (2.9)	4.5 (7.1)	2.0 (2.7)	3.5 (5.2)	2.0 (3.3)	2.5 (3.9)	3.2 (5.1)	1.9 (3.0)	2.5 (3.7)
	America	4.5 (7.1)	3.7 (5.9)	11.0 (15.4)	7.0 (10.5)	3.4 (5.6)	6.6 (10.4)	6.8 (11.1)	5.1 (8.2)	4.8 (7.1)
	Oceania	0.3 (0.4)	0.3 (0.4)	0.3 (0.5)	0.2 (0.3)	0.3 (0.4)	0.2 (0.4)	0.3 (0.4)	0.2 (0.4)	0.1 (0.1)
	Regional and Unspecified	11.0 (17.4)	7.3 (11.6)	7.7 (10.8)	6.0 (9.0)	5.1 (8.4)	9.9 (15.7)	11.8 (19.2)	10.1 (16.3)	18.4 (27.0)
Netherlands	Africa	297.4 (53.5)	243.5 (49.9)	294.7 (55.1)	271.9 (53.9)	281.2 (57.5)	272.6 (47.9)	245.8 (46.9)	274.9 (45.0)	252.5 (48.2)
	Asia	103.9 (18.7)	90.1 (18.5)	103.2 (19.3)	83.6 (16.6)	75.7 (15.5)	89.6 (15.7)	82.5 (15.7)	81.9 (13.4)	70.9 (13.5)
	Europe	7.0 (1.3)	5.2 (1.1)	5.4 (1.0)	5.5 (1.1)	4.0 (0.8)	6.7 (1.2)	10.6 (2.0)	4.8 (0.8)	6.0 (1.1)
	America	26.8 (4.8)	19.4 (4.0)	18.7 (3.5)	11.4 (2.3)	9.5 (2.0)	9.1 (1.6)	8.3 (1.6)	7.1 (1.2)	6.5 (1.2)
	Oceania	1.7 (0.3)	1.7 (0.4)	2.1 (0.4)	1.6 (0.3)	1.5 (0.3)	1.5 (0.3)	1.5 (0.3)	1.2 (0.2)	1.6 (0.3)
	Regional and Unspecified	119.4 (21.5)	128.2 (26.3)	110.8 (20.7)	130.3 (25.8)	116.9 (23.9)	189.9 (33.4)	175.4 (33.5)	241.5 (39.5)	186.1 (35.5)
New Zealand	Africa	6.2 (16.9)	3.6 (10.1)	4.6 (14.1)	3.6 (12.4)	3.3 (15.0)	3.2 (16.4)	3.0 (14.8)	2.7 (10.9)	2.3 (9.4)
	Asia	5.6 (15.3)	4.8 (13.5)	4.7 (14.5)	3.9 (13.7)	3.4 (15.3)	2.6 (13.2)	2.6 (12.6)	2.5 (9.9)	2.1 (8.5)
	Europe	0.1 (0.4)	0.2 (0.5)	0.1 (0.4)	0.1 (0.4)	0.1 (0.6)	0.1 (0.6)	0.1 (0.6)	0.1 (0.5)	0.1 (0.4)
	America	0.8 (2.1)	0.7 (2.0)	0.6 (1.9)	0.6 (1.9)	0.4 (2.0)	0.4 (2.0)	0.4 (1.7)	0.3 (1.4)	0.3 (1.1)
	Oceania	20.4 (55.7)	22.3 (63.0)	19.4 (59.6)	17.6 (61.4)	12.4 (56.1)	10.7 (54.1)	8.6 (42.0)	16.6 (66.2)	17.3 (71.8)
	Regional and Unspecified	3.6 (9.7)	3.9 (10.9)	3.1 (9.5)	2.9 (10.2)	2.5 (11.2)	2.7 (13.7)	5.8 (28.2)	2.8 (11.2)	2.1 (8.7)
Norway	Africa	180.7 (47.7)	189.2 (48.4)	206.9 (43.6)	222.8 (44.0)	239.8 (45.9)	204.5 (40.2)	203.3 (37.7)	192.2 (37.3)	194.7 (38.8)
	Asia	85.4 (22.5)	88.2 (22.5)	101.6 (21.4)	102.9 (20.3)	105.2 (20.1)	105.5 (20.7)	114.9 (21.3)	93.6 (18.2)	79.3 (15.8)
	Europe	3.5 (0.9)	5.0 (1.3)	4.8 (1.0)	6.0 (1.2)	5.3 (1.0)	3.4 (0.7)	2.5 (0.5)	2.6 (0.5)	3.6 (0.7)
	America	16.2 (4.3)	15.2 (3.9)	15.2 (3.2)	14.3 (2.8)	13.3 (2.5)	9.2 (1.8)	11.5 (2.1)	9.6 (1.9)	8.6 (1.7)
	Oceania	1.6 (0.4)	2.1 (0.5)	2.0 (0.4)	1.8 (0.4)	2.3 (0.4)	1.3 (0.3)	1.4 (0.3)	1.4 (0.3)	1.9 (0.4)
	Regional and Unspecified	91.7 (24.2)	91.5 (23.4)	144.4 (30.4)	158.4 (31.3)	156.3 (29.9)	184.7 (36.3)	205.1 (38.1)	215.6 (41.9)	213.8 (42.6)
Poland	Africa	NA	NA	6.8 (44.4)	7.6 (54.8)	10.6 (59.5)	12.4 (50.7)	10.3 (41.9)	8.3 (44.9)	11.1 (31.3)
	Asia	NA	NA	4.9 (31.8)	3.1 (22.0)	4.2 (23.5)	6.4 (26.1)	5.3 (21.7)	3.8 (20.6)	16.3 (45.8)
	Europe	NA	NA	0.7 (4.3)	0.8 (5.6)	0.8 (4.5)	2.5 (10.4)	5.3 (21.6)	2.5 (13.3)	3.5 (9.8)
	America	NA	NA	0.9 (6.0)	0.5 (3.6)	0.6 (3.3)	0.7 (3.0)	0.6 (2.6)	0.7 (3.7)	0.8 (2.1)
	Oceania	NA	NA	0.0 (0.3)	0.1 (0.4)	0.1 (0.6)	0.1 (0.3)	0.1 (0.4)	0.1 (0.4)	0.1 (0.4)
	Regional and Unspecified	NA	NA	2.0 (13.1)	1.9 (13.6)	1.5 (8.6)	2.3 (9.5)	2.9 (11.9)	3.2 (17.2)	3.8 (10.6)
Portugal	Africa	20.3 (75.8)	16.5 (85.9)	19.2 (75.0)	21.3 (91.1)	29.0 (90.3)	17.8 (78.0)	11.0 (56.8)	11.9 (71.5)	17.1 (74.6)
	Asia	4.1 (15.2)	1.5 (8.0)	3.3 (12.9)	1.2 (5.0)	1.7 (5.4)	2.4 (10.7)	4.1 (21.2)	2.3 (14.0)	2.4 (10.6)
	Europe	0.6 (2.3)	0.3 (1.6)	0.4 (1.4)	0.2 (0.7)	0.2 (0.6)	1.0 (4.4)	2.3 (12.1)	0.8 (5.0)	1.2 (5.3)
	America	0.5 (2.0)	0.4 (2.2)	0.7 (2.8)	0.2 (0.7)	0.3 (0.9)	0.4 (1.6)	0.5 (2.4)	0.3 (1.8)	0.4 (1.9)
	Oceania	0.1 (0.2)	0.0 (0.1)	0.1 (0.3)	0.0 (0.0)	0.0 (0.1)	0.0 (0.2)	0.0 (0.2)	0.0 (0.2)	0.1 (0.2)
	Regional and Unspecified	1.2 (4.5)	0.4 (2.2)	1.9 (7.6)	0.6 (2.6)	0.9 (2.7)	1.2 (5.1)	1.4 (7.3)	1.2 (7.5)	1.7 (7.3)
Slovak Republic	Africa	NA	NA	1.5 (47.5)	1.7 (58.5)	2.3 (60.4)	2.1 (48.4)	3.2 (48.8)	2.1 (41.7)	2.0 (40.1)
	Asia	NA	NA	1.1 (34.4)	0.6 (19.9)	0.9 (23.1)	1.3 (29.7)	1.6 (24.9)	1.8 (36.0)	1.0 (20.2)
	Europe	NA	NA	0.1 (2.1)	0.2 (6.1)	0.2 (5.8)	0.4 (9.4)	1.0 (15.7)	0.4 (9.0)	1.2 (23.6)
	America	NA	NA	0.2 (4.9)	0.1 (3.0)	0.1 (3.0)	0.1 (2.7)	0.1 (1.8)	0.1 (2.5)	0.1 (2.5)
	Oceania	NA	NA	0.0 (0.3)	0.0 (0.4)	0.0 (0.4)	0.0 (0.3)	0.0 (0.2)	0.0 (0.2)	0.0 (0.4)
	Regional and Unspecified	NA	NA	0.3 (10.9)	0.4 (12.0)	0.3 (7.3)	0.4 (9.5)	0.6 (8.5)	0.5 (10.6)	0.6 (13.2)
Slovenia	Africa	1.0 (44.2)	1.6 (52.1)	1.6 (61.4)	0.9 (51.5)	1.2 (56.4)	1.3 (49.6)	1.3 (41.6)	0.9 (47.7)	1.0 (41.9)
	Asia	0.8 (33.7)	0.6 (20.2)	0.6 (24.2)	0.4 (26.1)	0.5 (25.0)	0.8 (28.4)	0.8 (25.3)	0.4 (21.2)	0.6 (25.4)
	Europe	0.2 (8.2)	0.4 (13.5)	0.1 (2.1)	0.1 (6.9)	0.1 (6.8)	0.3 (10.4)	0.6 (19.5)	0.3 (13.5)	0.3 (13.3)
	America	0.1 (3.4)	0.2 (5.2)	0.1 (3.9)	0.1 (3.3)	0.1 (3.3)	0.1 (2.8)	0.1 (2.5)	0.1 (3.0)	0.1 (2.9)
	Oceania	0.0 (0.3)	0.0 (0.4)	0.0 (0.2)	0.0 (0.4)	0.0 (0.4)	0.0 (0.3)	0.0 (0.3)	0.0 (0.3)	0.0 (0.4)
	Regional and Unspecified	0.2 (10.2)	0.3 (8.6)	0.2 (8.2)	0.2 (11.8)	0.2 (8.1)	0.2 (8.6)	0.3 (10.8)	0.3 (14.4)	0.4 (16.0)

South Korea	Africa	26.6 (19.1)	34.3 (19.0)	74.3 (37.0)	66.9 (33.9)	100.6 (48.0)	110.6 (44.8)	90.6 (37.4)	69.6 (30.5)	73.1 (32.2)
	Asia	77.4 (55.5)	110.1 (60.9)	103.3 (51.5)	89.5 (45.3)	82.1 (39.2)	96.1 (39.0)	113.4 (46.8)	114.8 (50.2)	115.8 (51.1)
	Europe	15.2 (10.9)	13.6 (7.5)	3.5 (1.7)	14.0 (7.1)	0.8 (0.4)	3.9 (1.6)	0.9 (0.4)	0.5 (0.2)	0.7 (0.3)
	America	13.8 (9.9)	12.8 (7.1)	11.3 (5.6)	15.2 (7.7)	11.7 (5.6)	18.7 (7.6)	22.1 (9.1)	22.9 (10.0)	19.5 (8.6)
	Oceania	1.6 (1.2)	1.5 (0.8)	1.6 (0.8)	2.0 (1.0)	3.7 (1.7)	1.9 (0.8)	2.9 (1.2)	3.7 (1.6)	3.5 (1.5)
	Regional and Unspecified	4.9 (3.5)	8.5 (4.7)	6.6 (3.3)	9.8 (4.9)	10.7 (5.1)	15.5 (6.3)	12.5 (5.1)	17.1 (7.5)	14.4 (6.3)
Spain	Africa	103.9 (44.0)	46.7 (43.7)	60.5 (48.1)	59.3 (56.7)	49.6 (56.1)	68.8 (48.6)	66.1 (41.7)	59.2 (47.3)	63.6 (49.6)
	Asia	53.4 (22.6)	14.4 (13.5)	24.0 (19.1)	13.5 (13.0)	14.4 (16.2)	27.5 (19.4)	36.4 (23.0)	23.2 (18.6)	21.6 (16.8)
	Europe	3.9 (1.7)	2.3 (2.2)	2.2 (1.7)	1.9 (1.8)	1.2 (1.4)	6.9 (4.9)	16.5 (10.4)	5.3 (4.2)	7.8 (6.1)
	America	55.8 (23.6)	32.2 (30.2)	23.0 (18.3)	19.8 (18.9)	15.8 (17.9)	21.6 (15.3)	19.8 (12.5)	20.1 (16.1)	17.7 (13.8)
	Oceania	0.4 (0.2)	0.2 (0.2)	0.3 (0.3)	0.2 (0.2)	0.2 (0.2)	0.4 (0.3)	0.3 (0.2)	0.2 (0.1)	0.4 (0.3)
	Regional and Unspecified	18.5 (7.8)	10.9 (10.2)	15.6 (12.4)	9.8 (9.4)	7.2 (8.2)	16.4 (11.6)	19.4 (12.3)	17.2 (13.7)	17.0 (13.3)
Sweden	Africa	241.2 (55.0)	271.3 (58.2)	261.7 (54.7)	221.7 (51.3)	247.9 (54.0)	264.6 (57.0)	273.1 (53.6)	275.9 (54.1)	278.9 (60.2)
	Asia	103.7 (23.6)	94.7 (20.3)	115.4 (24.1)	98.8 (22.9)	103.1 (22.4)	91.6 (19.7)	112.9 (22.2)	104.7 (20.5)	88.0 (19.0)
	Europe	6.1 (1.4)	6.2 (1.3)	5.0 (1.0)	4.7 (1.1)	5.2 (1.1)	5.9 (1.3)	8.5 (1.7)	5.4 (1.0)	5.7 (1.2)
	America	21.1 (4.8)	17.7 (3.8)	16.4 (3.4)	14.9 (3.4)	14.3 (3.1)	12.8 (2.8)	14.3 (2.8)	16.8 (3.3)	14.8 (3.2)
	Oceania	1.4 (0.3)	2.4 (0.5)	2.1 (0.4)	1.6 (0.4)	2.1 (0.5)	1.9 (0.4)	1.7 (0.3)	1.8 (0.4)	1.8 (0.4)
	Regional and Unspecified	65.1 (14.8)	74.2 (15.9)	78.2 (16.3)	90.7 (21.0)	86.5 (18.8)	87.7 (18.9)	98.8 (19.4)	105.7 (20.7)	74.1 (16.0)
Switzerland	Africa	74.0 (52.0)	72.9 (50.5)	77.9 (52.7)	109.2 (53.2)	106.4 (53.6)	90.6 (48.9)	93.1 (43.5)	100.0 (49.2)	98.0 (48.7)
	Asia	40.2 (28.2)	39.8 (27.6)	38.3 (26.0)	50.9 (24.8)	44.5 (22.4)	48.5 (26.2)	64.4 (30.1)	47.4 (23.3)	47.5 (23.6)
	Europe	9.2 (6.5)	12.1 (8.4)	12.4 (8.4)	17.4 (8.5)	17.8 (9.0)	16.4 (8.9)	19.4 (9.1)	14.3 (7.0)	14.6 (7.2)
	America	5.4 (3.8)	4.7 (3.2)	3.3 (2.2)	3.7 (1.8)	3.9 (2.0)	3.7 (2.0)	6.0 (2.8)	5.4 (2.7)	10.2 (5.1)
	Oceania	0.5 (0.3)	0.6 (0.4)	0.4 (0.3)	0.5 (0.2)	0.6 (0.3)	0.5 (0.3)	0.4 (0.2)	0.4 (0.2)	0.5 (0.2)
	Regional and Unspecified	13.2 (9.2)	14.3 (9.9)	15.3 (10.4)	23.6 (11.5)	25.4 (12.8)	25.4 (13.7)	30.9 (14.4)	35.6 (17.5)	30.6 (15.2)
United Kingdom	Africa	895.0 (43.5)	1105.9 (50.7)	1665.0 (50.8)	1427.1 (50.6)	1163.9 (52.3)	1240.5 (56.5)	1411.9 (50.3)	1499.8 (52.2)	1559.1 (54.5)
	Asia	525.2 (25.5)	485.7 (22.2)	772.2 (23.6)	568.5 (20.2)	460.9 (20.7)	398.8 (18.2)	572.4 (20.4)	497.7 (17.3)	427.5 (14.9)
	Europe	17.2 (0.8)	13.4 (0.6)	27.3 (0.8)	17.8 (0.6)	8.0 (0.4)	17.8 (0.8)	68.5 (2.4)	38.3 (1.3)	33.3 (1.2)
	America	41.1 (2.0)	29.9 (1.4)	57.3 (1.7)	30.6 (1.1)	24.6 (1.1)	35.8 (1.6)	45.7 (1.6)	52.8 (1.8)	54.6 (1.9)
	Oceania	3.8 (0.2)	5.3 (0.2)	12.3 (0.4)	6.3 (0.2)	5.2 (0.2)	4.0 (0.2)	5.2 (0.2)	6.4 (0.2)	8.0 (0.3)
	Regional and Unspecified	574.0 (27.9)	543.0 (24.9)	741.7 (22.6)	769.8 (27.3)	561.4 (25.2)	497.5 (22.7)	703.6 (25.1)	779.4 (27.1)	780.1 (27.3)
United States	Africa	5191.9 (52.4)	5630.1 (56.9)	5927.6 (58.0)	6221.7 (59.5)	5027.2 (54.8)	6499.5 (58.5)	6293.7 (57.0)	6118.0 (59.6)	4687.4 (58.2)
	Asia	1266.0 (12.8)	1265.2 (12.8)	1320.5 (12.9)	1374.7 (13.1)	1114.3 (12.2)	1247.5 (11.2)	1246.3 (11.3)	949.4 (9.3)	866.4 (10.8)
	Europe	65.6 (0.7)	75.6 (0.8)	74.0 (0.7)	91.9 (0.9)	52.2 (0.6)	80.1 (0.7)	54.7 (0.5)	54.5 (0.5)	42.1 (0.5)
	America	468.8 (4.7)	443.4 (4.5)	471.4 (4.6)	384.8 (3.7)	365.4 (4.0)	395.3 (3.6)	344.2 (3.1)	308.3 (3.0)	197.0 (2.4)
	Oceania	16.4 (0.2)	62.6 (0.6)	27.9 (0.3)	60.5 (0.6)	30.5 (0.3)	29.1 (0.3)	20.9 (0.2)	21.5 (0.2)	19.2 (0.2)
	Regional and Unspecified	2898.0 (29.3)	2413.2 (24.4)	2404.1 (23.5)	2329.6 (22.3)	2577.7 (28.1)	2855.1 (25.7)	3075.5 (27.9)	2809.9 (27.4)	2237.4 (27.8)
All members	Africa	9334.9 (50.2)	10090.6 (54.0)	11073.0 (54.5)	11245.6 (55.3)	9903.9 (54.4)	11439.9 (56.0)	11435.3 (53.3)	11416.2 (55.0)	10164.4 (54.8)
	Asia	3730.1 (20.0)	3611.8 (19.3)	3980.9 (19.6)	3691.2 (18.2)	3358.0 (18.5)	3584.8 (17.6)	3877.7 (18.1)	3278.0 (15.8)	3134.2 (16.9)
	Europe	229.3 (1.2)	227.5 (1.2)	200.0 (1.0)	254.1 (1.2)	196.2 (1.1)	247.4 (1.2)	368.6 (1.7)	234.6 (1.1)	232.6 (1.3)
	America	942.6 (5.1)	831.4 (4.4)	936.6 (4.6)	997.2 (4.9)	650.3 (3.6)	723.7 (3.5)	689.8 (3.2)	676.1 (3.3)	544.0 (2.9)
	Oceania	238.8 (1.3)	261.6 (1.4)	241.5 (1.2)	237.4 (1.2)	210.4 (1.2)	166.8 (0.8)	163.7 (0.8)	188.7 (0.9)	217.7 (1.2)
	Regional and Unspecified	4131.6 (22.2)	3661.8 (19.6)	3883.5 (19.1)	3906.3 (19.2)	3881.1 (21.3)	4262.7 (20.9)	4912.7 (22.9)	4975.9 (24.0)	4242.1 (22.9)

DAH: development assistance for health; DAH: Development Assistance Committee

Table S6: Estimated DAH in constant prices at 2019 from all the 29 DAC member countries by health focus area (%), 2011–2019

Country	Health focus area	2011	2012	2013	2014	2015	2016	2017	2018	2019
Australia	1	90.5 (18.8)	152.1 (27.2)	103.4 (22.4)	136.6 (31.0)	43.9 (11.9)	63.5 (18.4)	59.8 (24.0)	47.6 (14.6)	81.0 (26.4)
	2	5.9 (1.2)	1.3 (0.2)	1.0 (0.2)	1.1 (0.2)	41.8 (11.4)	1.4 (0.4)	13.8 (5.6)	16.3 (5.0)	49.3 (16.1)
	3	12.6 (2.6)	12.9 (2.3)	11.2 (2.4)	12.8 (2.9)	10.6 (2.9)	10.3 (3.0)	4.2 (1.7)	13.5 (4.2)	6.8 (2.2)
	4	1.7 (0.4)	5.4 (1.0)	1.3 (0.3)	2.0 (0.5)	4.7 (1.3)	7.7 (2.2)	5.8 (2.3)	3.3 (1.0)	4.0 (1.3)
	5	1.2 (0.2)	6.7 (1.2)	3.0 (0.7)	1.7 (0.4)	3.2 (0.9)	0.8 (0.2)	4.0 (1.6)	2.2 (0.7)	2.5 (0.8)
	6	67.3 (14.0)	51.3 (9.2)	56.1 (12.1)	30.9 (7.0)	39.1 (10.6)	20.8 (6.0)	16.8 (6.7)	17.8 (5.5)	11.3 (3.7)
	7	7.4 (1.5)	3.5 (0.6)	5.4 (1.2)	8.0 (1.8)	0.9 (0.2)	1.7 (0.5)	9.0 (3.6)	7.0 (2.2)	8.1 (2.6)
	8	21.3 (4.4)	20.4 (3.6)	16.5 (3.6)	39.4 (8.9)	43.4 (11.8)	68.5 (19.9)	22.3 (8.9)	55.9 (17.2)	39.5 (12.9)
	9	11.2 (2.3)	23.5 (4.2)	25.2 (5.5)	24.9 (5.6)	17.9 (4.9)	23.0 (6.7)	10.3 (4.1)	25.5 (7.8)	15.7 (5.1)
	10	5.9 (1.2)	8.2 (1.5)	15.9 (3.4)	4.4 (1.0)	9.1 (2.5)	12.8 (3.7)	6.9 (2.8)	23.0 (7.1)	17.9 (5.9)
	11	92.3 (19.2)	115.8 (20.7)	114.3 (24.8)	101.5 (23.0)	82.7 (22.5)	75.8 (22.0)	17.8 (7.1)	33.8 (10.4)	7.9 (2.6)
	12	133.6 (27.8)	118.1 (21.1)	78.9 (17.1)	60.5 (13.7)	48.5 (13.2)	38.8 (11.2)	50.6 (20.3)	69.3 (21.3)	45.8 (15.0)
	13	14.3 (3.0)	9.6 (1.7)	7.3 (1.6)	3.2 (0.7)	1.5 (0.4)	1.9 (0.5)	1.3 (0.5)	0.7 (0.2)	0.7 (0.2)
	14	4.6 (1.0)	3.5 (0.6)	7.8 (1.7)	0.9 (0.2)	9.2 (2.5)	8.7 (2.5)	1.9 (0.8)	0.8 (0.2)	2.1 (0.7)
	15	6.3 (1.3)	7.2 (1.3)	4.8 (1.0)	4.0 (0.9)	4.4 (1.2)	3.8 (1.1)	6.6 (2.7)	6.0 (1.9)	8.1 (2.7)
	16	3.8 (0.8)	11.3 (2.0)	8.7 (1.9)	8.7 (2.0)	5.5 (1.5)	3.8 (1.1)	1.6 (0.6)	2.1 (0.7)	3.2 (1.0)
	17	0.5 (0.1)	7.9 (1.4)	0.9 (0.2)	0.0 (0.0)	1.3 (0.4)	1.8 (0.5)	16.4 (6.6)	0.3 (0.1)	1.5 (0.5)
	18	NA	NA	NA	NA	NA	NA	NA	0.0 (0.0)	0.7 (0.2)
	19	NA	NA	NA	NA	NA	NA	NA	0.0 (0.0)	0.0 (0.0)
	20	NA	NA	NA	NA	NA	NA	NA	0.0 (0.0)	0.0 (0.0)
	21	NA	NA	NA	NA	NA	NA	NA	0.0 (0.0)	0.0 (0.0)
	22	NA	NA	NA	NA	NA	NA	NA	0.0 (0.0)	0.1 (0.0)
	23	NA	NA	NA	NA	NA	NA	NA	0.0 (0.0)	0.0 (0.0)
Austria	1	10.8 (23.2)	8.7 (22.2)	10.6 (27.4)	7.9 (18.7)	6.3 (16.4)	9.3 (20.9)	9.6 (20.0)	7.7 (20.3)	8.9 (19.5)
	2	0.9 (1.9)	1.7 (4.3)	1.3 (3.3)	3.9 (9.4)	1.0 (2.7)	2.1 (4.6)	6.5 (13.6)	2.1 (5.6)	3.2 (7.0)
	3	1.4 (2.9)	1.3 (3.4)	1.7 (4.4)	2.9 (7.0)	2.6 (6.7)	6.9 (15.5)	5.7 (12.0)	5.0 (13.1)	4.6 (10.1)
	4	0.3 (0.6)	0.3 (0.9)	0.4 (1.1)	0.5 (1.1)	0.4 (1.0)	0.4 (0.9)	0.4 (0.9)	0.5 (1.2)	0.5 (1.1)
	5	0.5 (1.1)	0.6 (1.5)	0.3 (0.8)	0.3 (0.7)	0.4 (1.0)	0.2 (0.5)	0.2 (0.4)	0.2 (0.6)	0.5 (1.2)
	6	2.7 (5.8)	2.0 (5.1)	2.6 (6.6)	1.6 (3.9)	1.9 (4.9)	3.3 (7.4)	2.3 (4.7)	1.8 (4.8)	2.1 (4.6)
	7	0.0 (0.0)	0.3 (0.7)	0.1 (0.2)	0.1 (0.3)	0.1 (0.2)	0.3 (0.7)	0.5 (1.0)	0.7 (1.9)	0.3 (0.8)
	8	4.0 (8.5)	2.9 (7.3)	4.0 (10.4)	7.3 (17.3)	8.6 (22.3)	2.1 (4.6)	3.4 (7.2)	1.8 (4.8)	3.7 (8.2)
	9	1.3 (2.7)	1.3 (3.2)	0.9 (2.3)	1.1 (2.5)	0.9 (2.2)	0.9 (1.9)	1.3 (2.7)	1.1 (2.9)	0.8 (1.7)
	10	0.4 (0.9)	0.2 (0.6)	0.3 (0.8)	0.2 (0.4)	0.2 (0.5)	0.5 (1.1)	0.5 (1.0)	0.3 (0.9)	0.4 (0.9)
	11	3.7 (7.9)	2.6 (6.6)	2.0 (5.3)	1.3 (3.0)	1.5 (3.8)	0.9 (2.1)	0.7 (1.5)	0.5 (1.3)	0.8 (1.8)
	12	10.7 (23.0)	10.0 (25.5)	8.9 (23.1)	7.6 (18.1)	6.3 (16.4)	7.0 (15.6)	9.1 (19.0)	7.9 (20.7)	9.0 (19.7)
	13	1.1 (2.4)	1.4 (3.7)	1.0 (2.5)	0.6 (1.5)	0.4 (1.1)	0.5 (1.1)	0.3 (0.7)	0.4 (1.1)	0.2 (0.5)
	14	0.2 (0.3)	0.1 (0.2)	0.0 (0.1)	0.2 (0.4)	0.2 (0.5)	0.2 (0.4)	0.2 (0.3)	0.1 (0.3)	0.2 (0.5)
	15	6.8 (14.6)	4.8 (12.3)	3.5 (9.2)	5.4 (12.7)	6.0 (15.6)	8.5 (19.1)	4.3 (8.9)	6.9 (18.1)	8.9 (19.5)
	16	1.9 (4.1)	1.0 (2.5)	0.9 (2.3)	1.2 (2.9)	1.8 (4.6)	1.5 (3.5)	2.8 (5.8)	0.5 (1.3)	0.5 (1.0)
	17	0.0 (0.0)	0.0 (0.0)	0.0 (0.1)	0.0 (0.1)	0.1 (0.1)	0.1 (0.2)	0.1 (0.3)	0.3 (0.8)	0.1 (0.2)
	18	NA	NA	NA	NA	NA	NA	NA	0.0 (0.1)	0.8 (1.7)
	19	NA	NA	NA	NA	NA	NA	NA	0.0 (0.0)	0.0 (0.0)
	20	NA	NA	NA	NA	NA	NA	NA	0.0 (0.0)	0.0 (0.0)
	21	NA	NA	NA	NA	NA	NA	NA	0.0 (0.1)	0.0 (0.0)

	22	NA	NA	NA	NA	NA	NA	NA	0.0 (0.0)	0.0 (0.1)
	23	NA	NA	NA	NA	NA	NA	NA	0.0 (0.0)	0.0 (0.0)
Belgium	1	41.2 (16.8)	37.0 (16.2)	40.2 (17.9)	47.7 (19.8)	46.3 (22.0)	44.5 (21.8)	37.8 (17.9)	37.1 (20.0)	38.5 (20.9)
	2	3.7 (1.5)	5.8 (2.6)	5.1 (2.3)	1.7 (0.7)	2.3 (1.1)	5.3 (2.6)	12.2 (5.7)	9.0 (4.8)	8.3 (4.5)
	3	10.1 (4.1)	9.0 (3.9)	4.8 (2.1)	9.4 (3.9)	6.1 (2.9)	6.7 (3.3)	7.3 (3.5)	7.5 (4.0)	11.9 (6.5)
	4	2.6 (1.1)	2.1 (0.9)	1.7 (0.7)	2.4 (1.0)	0.9 (0.4)	1.1 (0.5)	2.3 (1.1)	1.3 (0.7)	1.4 (0.8)
	5	8.0 (3.3)	7.3 (3.2)	7.2 (3.2)	3.5 (1.4)	3.9 (1.9)	1.2 (0.6)	3.2 (1.5)	2.3 (1.2)	2.9 (1.5)
	6	11.8 (4.8)	7.4 (3.3)	8.5 (3.8)	9.5 (3.9)	5.8 (2.8)	11.1 (5.4)	10.7 (5.0)	11.5 (6.2)	13.0 (7.0)
	7	0.2 (0.1)	1.1 (0.5)	1.1 (0.5)	1.2 (0.5)	1.1 (0.5)	1.0 (0.5)	7.8 (3.7)	2.3 (1.2)	3.0 (1.6)
	8	14.1 (5.7)	13.6 (6.0)	13.3 (5.9)	10.9 (4.5)	13.1 (6.2)	9.6 (4.7)	13.2 (6.2)	19.2 (10.4)	23.0 (12.4)
	9	7.9 (3.2)	9.1 (4.0)	4.9 (2.2)	8.2 (3.4)	7.2 (3.4)	7.3 (3.6)	6.4 (3.0)	6.4 (3.4)	6.6 (3.6)
	10	17.3 (7.1)	16.1 (7.1)	14.7 (6.5)	14.4 (6.0)	14.2 (6.8)	16.4 (8.0)	9.2 (4.3)	8.2 (4.4)	8.5 (4.6)
	11	31.5 (12.8)	23.8 (10.5)	20.7 (9.2)	19.1 (7.9)	18.0 (8.5)	16.4 (8.0)	11.8 (5.6)	11.7 (6.3)	7.4 (4.0)
	12	53.6 (21.8)	51.3 (22.5)	64.9 (28.9)	65.8 (27.3)	44.5 (21.2)	36.6 (18.0)	46.0 (21.8)	34.7 (18.8)	25.8 (14.0)
	13	11.2 (4.5)	11.5 (5.0)	11.4 (5.1)	18.1 (7.5)	23.3 (11.1)	32.5 (16.0)	22.7 (10.7)	16.3 (8.8)	13.4 (7.3)
	14	14.5 (5.9)	14.6 (6.4)	14.4 (6.4)	12.1 (5.0)	9.1 (4.3)	2.0 (1.0)	7.6 (3.6)	6.2 (3.4)	6.4 (3.5)
	15	12.3 (5.0)	9.9 (4.4)	5.1 (2.3)	8.3 (3.5)	7.8 (3.7)	6.1 (3.0)	4.6 (2.2)	5.3 (2.9)	9.9 (5.4)
	16	5.6 (2.3)	6.7 (2.9)	6.1 (2.7)	7.8 (3.2)	4.6 (2.2)	5.1 (2.5)	7.0 (3.3)	4.9 (2.7)	1.8 (1.0)
	17	0.1 (0.0)	1.4 (0.6)	1.0 (0.4)	1.0 (0.4)	1.8 (0.9)	0.9 (0.4)	1.8 (0.9)	1.2 (0.6)	0.9 (0.5)
	18	NA	NA	NA	NA	NA	NA	NA	0.0 (0.0)	1.8 (1.0)
	19	NA	NA	NA	NA	NA	NA	NA	0.0 (0.0)	0.0 (0.0)
	20	NA	NA	NA	NA	NA	NA	NA	0.0 (0.0)	0.0 (0.0)
	21	NA	NA	NA	NA	NA	NA	NA	0.0 (0.0)	0.0 (0.0)
	22	NA	NA	NA	NA	NA	NA	NA	0.0 (0.0)	0.0 (0.0)
	23	NA	NA	NA	NA	NA	NA	NA	0.0 (0.0)	0.0 (0.0)
Canada	1	137.7 (16.9)	83.6 (10.4)	137.2 (15.8)	61.3 (10.1)	80.7 (9.3)	145.1 (18.5)	160.2 (18.2)	126.9 (14.1)	138.8 (16.4)
	2	19.9 (2.4)	17.4 (2.2)	45.1 (5.2)	27.4 (4.5)	23.2 (2.7)	23.2 (3.0)	24.2 (2.8)	17.9 (2.0)	17.1 (2.0)
	3	105.8 (13.0)	172.8 (21.4)	147.1 (16.9)	145.3 (23.9)	117.4 (13.6)	110.7 (14.1)	103.7 (11.8)	111.7 (12.4)	73.2 (8.7)
	4	8.4 (1.0)	13.5 (1.7)	14.2 (1.6)	14.9 (2.5)	12.5 (1.4)	20.7 (2.6)	23.6 (2.7)	24.8 (2.8)	21.5 (2.5)
	5	21.4 (2.6)	23.9 (3.0)	28.2 (3.3)	25.5 (4.2)	25.8 (3.0)	41.1 (5.2)	30.0 (3.4)	21.0 (2.3)	16.6 (2.0)
	6	25.6 (3.2)	29.0 (3.6)	30.5 (3.5)	29.8 (4.9)	30.2 (3.5)	30.0 (3.8)	51.9 (5.9)	70.8 (7.9)	77.5 (9.2)
	7	4.1 (0.5)	5.5 (0.7)	8.9 (1.0)	6.8 (1.1)	5.5 (0.6)	7.1 (0.9)	26.9 (3.1)	43.2 (4.8)	39.9 (4.7)
	8	95.1 (11.7)	62.1 (7.7)	97.0 (11.2)	87.5 (14.4)	109.6 (12.7)	86.3 (11.0)	104.6 (11.9)	93.0 (10.4)	91.6 (10.9)
	9	49.0 (6.0)	76.4 (9.5)	59.1 (6.8)	7.8 (1.3)	105.9 (12.2)	56.3 (7.2)	70.0 (8.0)	65.6 (7.3)	69.1 (8.2)
	10	75.7 (9.3)	48.4 (6.0)	42.6 (4.9)	1.0 (0.2)	52.9 (6.1)	41.7 (5.3)	40.2 (4.6)	59.1 (6.6)	43.6 (5.2)
	11	139.5 (17.2)	137.6 (17.1)	105.7 (12.2)	23.7 (3.9)	171.1 (19.8)	86.3 (11.0)	84.7 (9.6)	79.6 (8.9)	68.4 (8.1)
	12	79.4 (9.8)	69.3 (8.6)	61.1 (7.0)	56.4 (9.3)	42.8 (4.9)	50.6 (6.4)	61.9 (7.0)	60.3 (6.7)	47.6 (5.6)
	13	4.5 (0.5)	3.4 (0.4)	6.0 (0.7)	3.7 (0.6)	7.5 (0.9)	6.3 (0.8)	6.3 (0.7)	7.1 (0.8)	7.1 (0.8)
	14	16.2 (2.0)	38.2 (4.7)	44.3 (5.1)	45.7 (7.5)	33.9 (3.9)	26.8 (3.4)	6.5 (0.7)	8.5 (0.9)	7.4 (0.9)
	15	6.0 (0.7)	6.0 (0.7)	7.1 (0.8)	4.6 (0.8)	7.6 (0.9)	19.1 (2.4)	15.9 (1.8)	14.7 (1.6)	14.7 (1.7)
	16	7.5 (0.9)	10.2 (1.3)	12.3 (1.4)	44.7 (7.3)	16.6 (1.9)	14.3 (1.8)	25.3 (2.9)	46.2 (5.1)	61.7 (7.3)
	17	17.2 (2.1)	8.6 (1.1)	21.5 (2.5)	22.4 (3.7)	21.9 (2.5)	20.2 (2.6)	43.1 (4.9)	46.5 (5.2)	46.4 (5.5)
	18	NA	NA	NA	NA	NA	NA	NA	0.4 (0.0)	0.9 (0.1)
	19	NA	NA	NA	NA	NA	NA	NA	0.0 (0.0)	0.0 (0.0)
	20	NA	NA	NA	NA	NA	NA	NA	0.0 (0.0)	0.0 (0.0)
	21	NA	NA	NA	NA	NA	NA	NA	0.0 (0.0)	0.0 (0.0)
	22	NA	NA	NA	NA	NA	NA	NA	0.0 (0.0)	1.1 (0.1)

	23	NA	NA	NA	NA	NA	NA	NA	0.0 (0.0)	0.1 (0.0)
Czech Republic	1	2.7 (27.1)	2.2 (26.2)	3.1 (32.0)	1.8 (21.1)	2.2 (22.5)	3.0 (23.5)	5.6 (33.3)	2.1 (24.9)	2.9 (22.4)
	2	0.5 (5.4)	0.5 (6.6)	0.1 (1.5)	1.3 (15.4)	1.0 (10.2)	1.4 (10.8)	2.5 (14.7)	0.9 (10.5)	1.5 (11.9)
	3	0.4 (3.6)	0.3 (3.9)	0.5 (4.8)	1.1 (13.5)	0.9 (9.3)	1.6 (12.8)	1.5 (8.8)	1.0 (12.0)	1.3 (10.1)
	4	0.1 (0.6)	0.1 (1.1)	0.1 (1.2)	0.0 (0.5)	0.1 (0.7)	0.1 (1.0)	0.2 (1.3)	0.1 (1.3)	0.1 (0.4)
	5	0.1 (0.9)	0.1 (1.3)	0.1 (1.1)	0.0 (0.2)	0.1 (1.4)	0.1 (0.8)	0.1 (0.6)	0.1 (1.8)	1.7 (13.4)
	6	0.6 (5.8)	0.3 (4.0)	0.6 (6.1)	0.5 (6.3)	0.7 (6.9)	0.7 (5.7)	0.6 (3.5)	0.4 (5.4)	0.3 (2.4)
	7	0.0 (0.0)	0.1 (1.3)	0.0 (0.4)	0.1 (0.6)	0.0 (0.2)	0.0 (0.2)	0.2 (1.2)	0.1 (0.7)	0.0 (0.3)
	8	0.7 (7.3)	0.6 (6.9)	1.0 (9.8)	0.4 (4.6)	0.7 (7.2)	0.5 (3.9)	0.5 (2.7)	0.4 (4.7)	0.4 (3.1)
	9	0.1 (1.1)	0.1 (1.4)	0.1 (0.6)	0.0 (0.5)	0.0 (0.4)	0.1 (0.4)	0.1 (0.4)	0.1 (0.6)	0.1 (0.6)
	10	0.0 (0.4)	0.0 (0.3)	0.0 (0.3)	0.0 (0.1)	0.0 (0.4)	0.1 (0.4)	0.0 (0.3)	0.0 (0.4)	0.0 (0.4)
	11	0.5 (4.7)	0.6 (6.7)	0.2 (2.0)	0.1 (1.0)	0.2 (1.9)	0.1 (1.1)	0.1 (0.6)	0.0 (0.5)	0.1 (0.6)
	12	2.0 (19.7)	1.6 (19.7)	1.9 (19.6)	0.9 (10.1)	2.0 (20.6)	2.2 (17.2)	2.7 (15.9)	1.8 (22.0)	2.5 (19.7)
	13	0.1 (0.8)	0.3 (3.3)	0.2 (2.5)	0.3 (3.1)	0.2 (2.2)	0.4 (2.8)	0.2 (1.3)	0.1 (1.7)	0.1 (0.6)
	14	0.0 (0.3)	0.0 (0.3)	0.0 (0.2)	0.0 (0.1)	0.1 (1.1)	0.1 (0.8)	0.1 (0.9)	0.1 (0.8)	0.1 (0.8)
	15	1.6 (15.6)	1.0 (11.7)	1.4 (14.3)	1.5 (17.5)	1.0 (10.0)	1.6 (12.8)	1.2 (6.9)	0.9 (11.0)	1.1 (8.7)
	16	0.7 (6.6)	0.4 (5.3)	0.4 (3.8)	0.4 (5.3)	0.5 (5.0)	0.7 (5.7)	1.2 (7.4)	0.1 (1.4)	0.1 (0.7)
	17	0.0 (0.0)	0.0 (0.0)	0.0 (0.0)	0.0 (0.0)	0.0 (0.1)	0.0 (0.1)	0.0 (0.2)	0.0 (0.2)	0.0 (0.1)
	18	NA	NA	NA	NA	NA	NA	NA	0.0 (0.1)	0.4 (3.0)
	19	NA	NA	NA	NA	NA	NA	NA	0.0 (0.0)	0.0 (0.0)
	20	NA	NA	NA	NA	NA	NA	NA	0.0 (0.0)	0.0 (0.1)
	21	NA	NA	NA	NA	NA	NA	NA	0.0 (0.0)	0.0 (0.0)
	22	NA	NA	NA	NA	NA	NA	NA	0.0 (0.0)	0.1 (0.8)
	23	NA	NA	NA	NA	NA	NA	NA	0.0 (0.0)	0.0 (0.0)
Denmark	1	51.8 (21.9)	51.8 (24.6)	43.6 (23.3)	38.0 (17.2)	21.2 (13.1)	22.6 (15.5)	19.1 (10.8)	14.8 (7.4)	11.0 (6.5)
	2	2.7 (1.2)	2.7 (1.3)	0.6 (0.3)	3.3 (1.5)	0.7 (0.4)	1.5 (1.0)	4.2 (2.4)	1.5 (0.7)	2.2 (1.3)
	3	3.1 (1.3)	3.1 (1.5)	5.0 (2.7)	8.7 (3.9)	6.6 (4.1)	7.9 (5.4)	8.5 (4.8)	4.7 (2.3)	3.5 (2.0)
	4	2.5 (1.0)	2.2 (1.0)	1.2 (0.6)	0.5 (0.2)	0.4 (0.3)	0.2 (0.2)	0.4 (0.2)	0.2 (0.1)	0.5 (0.3)
	5	0.7 (0.3)	0.4 (0.2)	0.3 (0.1)	2.2 (1.0)	0.6 (0.3)	0.4 (0.2)	0.4 (0.2)	0.3 (0.1)	0.6 (0.4)
	6	41.4 (17.5)	23.2 (11.1)	34.4 (18.4)	40.9 (18.6)	35.3 (21.8)	21.7 (14.9)	53.3 (30.2)	42.3 (21.1)	48.8 (28.7)
	7	0.0 (0.0)	3.1 (1.5)	3.7 (2.0)	6.4 (2.9)	3.7 (2.3)	2.8 (1.9)	7.7 (4.4)	8.4 (4.2)	11.8 (6.9)
	8	5.7 (2.4)	3.2 (1.5)	4.1 (2.2)	9.4 (4.3)	8.4 (5.2)	3.2 (2.2)	2.5 (1.4)	9.2 (4.6)	7.0 (4.1)
	9	7.8 (3.3)	7.7 (3.7)	6.5 (3.5)	8.1 (3.7)	4.8 (2.9)	2.8 (1.9)	0.7 (0.4)	7.7 (3.8)	8.2 (4.8)
	10	5.0 (2.1)	3.6 (1.7)	4.7 (2.5)	4.1 (1.9)	2.6 (1.6)	1.7 (1.2)	0.4 (0.2)	3.8 (1.9)	3.6 (2.1)
	11	56.3 (23.8)	37.7 (17.9)	27.6 (14.7)	24.1 (11.0)	18.8 (11.6)	8.9 (6.1)	6.7 (3.8)	15.6 (7.8)	10.0 (5.9)
	12	41.3 (17.5)	40.6 (19.3)	27.6 (14.8)	33.7 (15.3)	44.9 (27.8)	57.0 (39.1)	41.9 (23.8)	35.8 (17.9)	40.5 (23.9)
	13	0.6 (0.3)	0.8 (0.4)	0.6 (0.3)	0.3 (0.2)	0.2 (0.1)	0.4 (0.3)	0.2 (0.1)	0.4 (0.2)	0.2 (0.1)
	14	0.3 (0.1)	0.0 (0.0)	2.1 (1.1)	0.8 (0.4)	0.4 (0.3)	0.4 (0.3)	0.4 (0.2)	0.3 (0.1)	0.5 (0.3)
	15	2.0 (0.8)	1.7 (0.8)	1.2 (0.7)	1.3 (0.6)	2.3 (1.4)	1.3 (0.9)	1.5 (0.9)	1.7 (0.8)	5.7 (3.4)
	16	15.2 (6.4)	28.4 (13.5)	24.2 (12.9)	38.4 (17.4)	8.5 (5.3)	9.5 (6.5)	24.0 (13.6)	52.7 (26.4)	12.7 (7.5)
	17	0.0 (0.0)	0.0 (0.0)	0.0 (0.0)	0.1 (0.0)	2.4 (1.5)	3.3 (2.3)	4.5 (2.6)	0.7 (0.4)	0.5 (0.3)
	18	NA	NA	NA	NA	NA	NA	NA	0.0 (0.0)	1.0 (0.6)
	19	NA	NA	NA	NA	NA	NA	NA	0.0 (0.0)	0.0 (0.0)
	20	NA	NA	NA	NA	NA	NA	NA	0.0 (0.0)	0.0 (0.0)
	21	NA	NA	NA	NA	NA	NA	NA	0.0 (0.0)	0.0 (0.0)
	22	NA	NA	NA	NA	NA	NA	NA	0.0 (0.0)	1.6 (0.9)
	23	NA	NA	NA	NA	NA	NA	NA	0.0 (0.0)	0.0 (0.0)

Finland	1	8.9 (8.4)	7.0 (6.6)	9.1 (8.6)	23.1 (16.4)	9.7 (10.4)	9.5 (18.0)	6.0 (10.6)	8.2 (16.5)	10.0 (21.3)
	2	1.7 (1.6)	1.3 (1.2)	1.3 (1.2)	1.0 (0.7)	1.2 (1.3)	1.4 (2.6)	3.8 (6.7)	1.4 (2.8)	1.8 (3.7)
	3	2.3 (2.1)	2.0 (1.9)	2.8 (2.6)	3.2 (2.2)	3.1 (3.4)	3.8 (7.2)	4.6 (8.2)	3.1 (6.2)	2.8 (5.9)
	4	6.1 (5.7)	6.4 (6.0)	5.6 (5.3)	4.8 (3.4)	5.9 (6.4)	3.4 (6.5)	4.6 (8.2)	2.7 (5.4)	3.0 (6.4)
	5	0.8 (0.8)	1.7 (1.6)	1.9 (1.8)	0.8 (0.6)	0.9 (1.0)	0.3 (0.6)	0.5 (0.9)	0.9 (1.9)	1.2 (2.5)
	6	27.7 (26.1)	17.0 (16.0)	17.2 (16.3)	15.3 (10.9)	8.1 (8.7)	7.1 (13.4)	11.8 (21.1)	7.9 (16.0)	8.3 (17.7)
	7	0.0 (0.0)	2.8 (2.7)	5.7 (5.4)	7.8 (5.5)	3.2 (3.5)	2.0 (3.8)	2.4 (4.3)	3.7 (7.5)	2.6 (5.5)
	8	2.6 (2.4)	2.1 (2.0)	4.3 (4.0)	14.6 (10.4)	8.5 (9.1)	1.4 (2.7)	1.7 (3.0)	1.6 (3.3)	1.6 (3.4)
	9	2.1 (1.9)	2.3 (2.2)	1.2 (1.2)	0.7 (0.5)	0.3 (0.4)	0.2 (0.4)	0.5 (0.8)	0.7 (1.4)	0.4 (0.8)
	10	1.3 (1.2)	1.1 (1.1)	1.0 (0.9)	0.6 (0.4)	0.6 (0.7)	0.5 (1.0)	0.5 (1.0)	0.4 (0.9)	0.4 (0.8)
	11	16.9 (15.9)	17.1 (16.1)	13.6 (12.9)	6.5 (4.6)	11.0 (11.8)	2.8 (5.4)	0.7 (1.3)	1.2 (2.4)	0.6 (1.3)
	12	14.9 (14.1)	16.5 (15.5)	8.6 (8.1)	10.7 (7.6)	9.4 (10.2)	6.1 (11.6)	8.5 (15.2)	6.4 (12.8)	6.2 (13.1)
	13	0.9 (0.8)	0.7 (0.6)	1.1 (1.0)	3.7 (2.6)	2.1 (2.3)	1.0 (1.9)	0.2 (0.3)	0.3 (0.6)	0.1 (0.2)
	14	0.1 (0.1)	0.0 (0.0)	0.1 (0.0)	0.6 (0.4)	0.6 (0.7)	0.3 (0.6)	0.5 (0.8)	0.2 (0.4)	0.3 (0.7)
	15	3.2 (3.0)	2.9 (2.7)	2.9 (2.7)	5.7 (4.0)	6.5 (7.0)	3.6 (6.9)	2.7 (4.9)	5.3 (10.8)	2.4 (5.0)
	16	17.0 (16.0)	25.2 (23.7)	29.5 (28.0)	42.1 (29.8)	15.6 (16.8)	5.9 (11.2)	4.7 (8.4)	4.9 (9.9)	3.8 (8.2)
	17	0.0 (0.0)	0.0 (0.0)	0.0 (0.0)	0.0 (0.0)	6.0 (6.5)	3.2 (6.1)	2.5 (4.4)	0.6 (1.2)	0.5 (1.1)
	18	NA	NA	NA	NA	NA	NA	NA	0.0 (0.0)	0.7 (1.4)
	19	NA	NA	NA	NA	NA	NA	NA	0.0 (0.0)	0.0 (0.0)
	20	NA	NA	NA	NA	NA	NA	NA	0.0 (0.0)	0.0 (0.0)
	21	NA	NA	NA	NA	NA	NA	NA	0.0 (0.0)	0.0 (0.0)
	22	NA	NA	NA	NA	NA	NA	NA	0.1 (0.1)	0.5 (1.1)
	23	NA	NA	NA	NA	NA	NA	NA	0.0 (0.0)	0.0 (0.0)
France	1	126.3 (16.6)	79.3 (10.5)	90.1 (10.7)	54.4 (5.1)	65.5 (8.8)	92.9 (10.9)	58.0 (7.0)	123.2 (14.1)	74.9 (8.2)
	2	3.6 (0.5)	4.5 (0.6)	5.7 (0.7)	4.6 (0.4)	6.7 (0.9)	12.1 (1.4)	45.3 (5.5)	30.2 (3.5)	38.1 (4.2)
	3	8.7 (1.1)	10.5 (1.4)	11.8 (1.4)	21.8 (2.0)	21.7 (2.9)	32.4 (3.8)	28.4 (3.4)	40.3 (4.6)	62.8 (6.9)
	4	1.2 (0.2)	1.7 (0.2)	24.1 (2.9)	1.3 (0.1)	1.9 (0.3)	1.1 (0.1)	3.0 (0.4)	3.9 (0.4)	3.1 (0.3)
	5	5.0 (0.7)	4.5 (0.6)	1.6 (0.2)	3.5 (0.3)	4.8 (0.6)	2.9 (0.3)	4.6 (0.6)	5.5 (0.6)	8.1 (0.9)
	6	29.6 (3.9)	36.1 (4.8)	37.2 (4.4)	45.2 (4.2)	34.9 (4.7)	19.2 (2.3)	25.2 (3.1)	30.6 (3.5)	23.3 (2.5)
	7	0.0 (0.0)	2.1 (0.3)	6.5 (0.8)	6.5 (0.6)	9.4 (1.3)	9.9 (1.2)	12.7 (1.5)	12.2 (1.4)	9.5 (1.0)
	8	40.9 (5.4)	32.4 (4.3)	40.5 (4.8)	44.0 (4.1)	123.3 (16.6)	185.0 (21.8)	87.8 (10.7)	101.0 (11.5)	116.5 (12.8)
	9	94.3 (12.4)	132.9 (17.6)	109.8 (13.0)	131.3 (12.2)	109.7 (14.7)	107.7 (12.7)	146.8 (17.8)	134.1 (15.3)	149.3 (16.3)
	10	61.8 (8.1)	64.9 (8.6)	80.7 (9.6)	68.6 (6.4)	57.4 (7.7)	62.3 (7.3)	77.9 (9.5)	65.8 (7.5)	65.8 (7.2)
	11	244.7 (32.2)	260.6 (34.5)	223.6 (26.5)	256.6 (23.9)	183.5 (24.6)	161.0 (18.9)	173.2 (21.0)	148.7 (17.0)	149.5 (16.4)
	12	71.8 (9.4)	60.5 (8.0)	129.2 (15.3)	325.7 (30.3)	49.5 (6.6)	66.0 (7.8)	81.6 (9.9)	69.7 (8.0)	144.2 (15.8)
	13	2.4 (0.3)	2.5 (0.3)	1.9 (0.2)	2.7 (0.2)	2.4 (0.3)	2.3 (0.3)	1.2 (0.1)	3.0 (0.3)	5.0 (0.6)
	14	19.2 (2.5)	9.2 (1.2)	16.5 (2.0)	9.7 (0.9)	10.9 (1.5)	12.9 (1.5)	8.4 (1.0)	33.5 (3.8)	20.6 (2.3)
	15	26.1 (3.4)	17.9 (2.4)	38.6 (4.6)	58.7 (5.5)	21.5 (2.9)	44.1 (5.2)	38.6 (4.7)	60.5 (6.9)	18.2 (2.0)
	16	23.1 (3.0)	27.8 (3.7)	8.2 (1.0)	21.6 (2.0)	22.9 (3.1)	11.0 (1.3)	17.4 (2.1)	4.6 (0.5)	6.5 (0.7)
	17	1.6 (0.2)	8.0 (1.1)	17.1 (2.0)	18.1 (1.7)	18.9 (2.5)	26.7 (3.1)	14.1 (1.7)	8.2 (0.9)	6.0 (0.7)
	18	NA	NA	NA	NA	NA	NA	NA	0.2 (0.0)	6.1 (0.7)
	19	NA	NA	NA	NA	NA	NA	NA	0.0 (0.0)	0.0 (0.0)
	20	NA	NA	NA	NA	NA	NA	NA	0.0 (0.0)	0.1 (0.0)
	21	NA	NA	NA	NA	NA	NA	NA	0.0 (0.0)	0.0 (0.0)
	22	NA	NA	NA	NA	NA	NA	NA	0.0 (0.0)	0.5 (0.1)
	23	NA	NA	NA	NA	NA	NA	NA	0.3 (0.0)	5.0 (0.6)
Germany	1	104.5 (11.4)	85.0 (9.6)	127.2 (13.5)	94.2 (9.5)	119.6 (11.9)	141.0 (12.0)	121.2 (9.2)	127.5 (9.3)	145.6 (10.3)

	2	48.8 (5.3)	56.7 (6.4)	56.2 (6.0)	76.7 (7.8)	89.1 (8.9)	180.9 (15.4)	206.8 (15.7)	122.7 (9.0)	144.8 (10.2)
	3	15.5 (1.7)	12.2 (1.4)	46.6 (4.9)	69.3 (7.0)	66.2 (6.6)	48.0 (4.1)	51.0 (3.9)	61.8 (4.5)	84.3 (6.0)
	4	3.8 (0.4)	5.3 (0.6)	4.3 (0.5)	4.3 (0.4)	4.9 (0.5)	5.5 (0.5)	6.4 (0.5)	8.4 (0.6)	8.8 (0.6)
	5	2.9 (0.3)	3.8 (0.4)	3.3 (0.3)	4.1 (0.4)	4.5 (0.4)	4.0 (0.3)	3.4 (0.3)	5.6 (0.4)	20.1 (1.4)
	6	57.6 (6.3)	78.0 (8.8)	87.5 (9.3)	102.4 (10.4)	112.1 (11.2)	138.8 (11.8)	106.0 (8.0)	96.2 (7.0)	93.6 (6.6)
	7	7.1 (0.8)	15.1 (1.7)	15.6 (1.7)	15.7 (1.6)	13.7 (1.4)	16.7 (1.4)	22.6 (1.7)	23.5 (1.7)	23.8 (1.7)
	8	59.3 (6.5)	69.8 (7.9)	107.0 (11.4)	104.6 (10.6)	156.9 (15.6)	170.2 (14.5)	265.5 (20.1)	332.3 (24.3)	280.9 (19.8)
	9	73.4 (8.0)	81.1 (9.2)	69.8 (7.4)	91.5 (9.3)	74.4 (7.4)	75.9 (6.5)	88.5 (6.7)	88.6 (6.5)	100.6 (7.1)
	10	53.7 (5.9)	41.5 (4.7)	51.4 (5.5)	48.9 (4.9)	42.3 (4.2)	48.4 (4.1)	51.3 (3.9)	46.1 (3.4)	46.2 (3.3)
	11	286.6 (31.3)	238.1 (27.0)	196.0 (20.8)	208.8 (21.1)	149.6 (14.9)	148.9 (12.7)	136.2 (10.3)	125.4 (9.2)	123.8 (8.7)
	12	146.3 (15.9)	130.5 (14.8)	120.5 (12.8)	105.4 (10.7)	115.5 (11.5)	122.6 (10.4)	155.6 (11.8)	194.6 (14.3)	173.3 (12.2)
	13	5.6 (0.6)	8.6 (1.0)	8.4 (0.9)	6.9 (0.7)	5.9 (0.6)	10.0 (0.8)	24.0 (1.8)	17.2 (1.3)	8.5 (0.6)
	14	3.3 (0.4)	4.8 (0.5)	5.0 (0.5)	4.6 (0.5)	10.2 (1.0)	12.2 (1.0)	22.6 (1.7)	39.6 (2.9)	58.8 (4.2)
	15	27.3 (3.0)	30.5 (3.5)	22.0 (2.3)	23.2 (2.3)	16.9 (1.7)	22.2 (1.9)	26.1 (2.0)	49.6 (3.6)	78.5 (5.5)
	16	21.2 (2.3)	21.7 (2.5)	21.5 (2.3)	27.2 (2.7)	17.4 (1.7)	18.4 (1.6)	25.4 (1.9)	10.3 (0.8)	7.4 (0.5)
	17	0.4 (0.0)	0.2 (0.0)	0.5 (0.1)	0.4 (0.0)	3.8 (0.4)	11.6 (1.0)	8.7 (0.7)	12.8 (0.9)	3.5 (0.2)
	18	NA	NA	NA	NA	NA	NA	NA	0.2 (0.0)	8.1 (0.6)
	19	NA	NA	NA	NA	NA	NA	NA	0.0 (0.0)	0.0 (0.0)
	20	NA	NA	NA	NA	NA	NA	NA	0.0 (0.0)	0.1 (0.0)
	21	NA	NA	NA	NA	NA	NA	NA	0.2 (0.0)	2.2 (0.2)
	22	NA	NA	NA	NA	NA	NA	NA	0.3 (0.0)	0.5 (0.0)
	23	NA	NA	NA	NA	NA	NA	NA	1.9 (0.1)	1.9 (0.1)
Greece	1	3.3 (29.7)	2.1 (26.3)	3.0 (32.4)	2.0 (26.9)	2.5 (29.8)	2.8 (27.7)	2.8 (21.7)	2.2 (26.5)	2.1 (25.4)
	2	0.1 (1.2)	0.1 (1.0)	0.1 (0.6)	0.0 (0.5)	0.1 (1.1)	0.8 (7.8)	2.5 (19.0)	0.8 (9.9)	1.3 (15.4)
	3	0.5 (4.4)	0.4 (5.3)	0.5 (5.7)	1.0 (13.2)	0.6 (7.4)	1.3 (13.2)	1.4 (10.9)	1.2 (14.6)	1.3 (16.0)
	4	0.1 (0.7)	0.1 (0.9)	0.1 (1.2)	0.0 (0.6)	0.0 (0.2)	0.0 (0.3)	0.0 (0.4)	0.0 (0.5)	0.0 (0.3)
	5	0.1 (1.3)	0.1 (1.3)	0.1 (1.5)	0.1 (1.7)	0.2 (2.1)	0.1 (1.4)	0.1 (0.8)	0.1 (0.9)	0.1 (1.6)
	6	0.3 (2.7)	0.3 (4.0)	0.5 (5.4)	0.5 (6.6)	0.6 (7.2)	0.5 (5.0)	0.4 (2.8)	0.3 (3.0)	0.1 (1.6)
	7	0.0 (0.0)	0.2 (2.1)	0.0 (0.3)	0.1 (0.8)	0.0 (0.2)	0.0 (0.1)	0.2 (1.7)	0.1 (1.0)	0.0 (0.4)
	8	0.8 (7.1)	0.4 (5.5)	1.1 (12.2)	0.5 (6.5)	0.6 (7.4)	0.5 (5.4)	0.6 (4.6)	0.5 (5.7)	0.3 (3.1)
	9	0.0 (0.1)	0.0 (0.1)	0.0 (0.2)	0.0 (0.6)	0.0 (0.6)	0.0 (0.4)	0.1 (0.9)	0.1 (1.1)	0.0 (0.2)
	10	0.0 (0.1)	0.0 (0.0)	0.0 (0.2)	0.0 (0.4)	0.0 (0.6)	0.0 (0.5)	0.1 (0.5)	0.0 (0.5)	0.0 (0.1)
	11	0.3 (2.7)	0.2 (2.0)	0.1 (1.3)	0.1 (1.2)	0.1 (1.4)	0.1 (0.9)	0.1 (0.8)	0.1 (0.8)	0.0 (0.2)
	12	3.4 (30.5)	3.0 (38.5)	2.3 (25.2)	1.7 (22.5)	2.3 (27.8)	2.3 (22.7)	2.8 (21.7)	2.1 (25.6)	2.2 (27.1)
	13	0.5 (4.0)	0.1 (1.0)	0.5 (5.1)	0.2 (3.2)	0.2 (2.1)	0.2 (1.9)	0.1 (0.5)	0.1 (1.6)	0.1 (0.7)
	14	0.1 (0.5)	0.0 (0.3)	0.0 (0.2)	0.1 (1.6)	0.2 (1.9)	0.1 (1.5)	0.1 (0.8)	0.1 (1.0)	0.0 (0.0)
	15	0.7 (6.0)	0.4 (4.7)	0.4 (4.5)	0.5 (6.8)	0.4 (4.4)	0.4 (4.1)	0.4 (3.1)	0.4 (5.0)	0.2 (2.8)
	16	1.0 (8.9)	0.6 (7.0)	0.4 (3.9)	0.5 (6.9)	0.5 (6.0)	0.7 (7.3)	1.2 (9.5)	0.1 (1.7)	0.1 (1.1)
	17	0.0 (0.1)	0.0 (0.0)	0.0 (0.0)	0.0 (0.0)	0.0 (0.0)	0.0 (0.0)	0.0 (0.3)	0.0 (0.4)	0.0 (0.1)
	18	NA	NA	NA	NA	NA	NA	NA	0.0 (0.1)	0.3 (3.7)
	19	NA	NA	NA	NA	NA	NA	NA	0.0 (0.0)	0.0 (0.0)
	20	NA	NA	NA	NA	NA	NA	NA	0.0 (0.0)	0.0 (0.0)
	21	NA	NA	NA	NA	NA	NA	NA	0.0 (0.0)	0.0 (0.0)
	22	NA	NA	NA	NA	NA	NA	NA	0.0 (0.0)	0.0 (0.2)
	23	NA	NA	NA	NA	NA	NA	NA	0.0 (0.0)	0.0 (0.0)
Hungary	1	NA	NA	NA	1.3 (24.4)	1.6 (21.6)	2.1 (27.5)	1.3 (20.1)	1.8 (13.4)	1.4 (11.0)
	2	NA	NA	NA	0.1 (1.2)	0.1 (1.7)	0.5 (7.3)	1.1 (17.7)	1.7 (12.1)	0.9 (6.6)

	3	NA	NA	NA	0.6 (11.5)	0.5 (7.1)	1.0 (13.4)	0.6 (9.7)	0.8 (6.0)	1.0 (7.4)
	4	NA	NA	NA	0.0 (0.7)	0.0 (0.5)	0.0 (0.5)	0.0 (0.3)	0.0 (0.2)	0.0 (0.2)
	5	NA	NA	NA	0.1 (1.2)	0.1 (1.6)	0.1 (0.9)	0.1 (1.1)	0.1 (0.6)	0.1 (1.1)
	6	NA	NA	NA	0.3 (5.5)	0.4 (5.3)	0.4 (5.8)	0.2 (2.6)	0.2 (1.8)	0.2 (1.5)
	7	NA	NA	NA	0.0 (0.7)	0.0 (0.2)	0.0 (0.4)	0.1 (1.6)	0.0 (0.3)	0.0 (0.2)
	8	NA	NA	NA	0.4 (8.1)	0.9 (12.0)	0.4 (5.1)	0.3 (4.2)	1.3 (9.2)	0.3 (1.9)
	9	NA	NA	NA	0.1 (1.5)	0.0 (0.5)	0.1 (0.8)	0.0 (0.8)	0.1 (0.5)	0.0 (0.3)
	10	NA	NA	NA	0.0 (0.4)	0.0 (0.5)	0.1 (0.8)	0.0 (0.5)	0.0 (0.3)	0.0 (0.2)
	11	NA	NA	NA	0.1 (2.2)	0.2 (2.4)	0.1 (1.4)	0.1 (0.9)	0.1 (0.7)	0.0 (0.4)
	12	NA	NA	NA	1.1 (20.1)	1.3 (18.3)	1.5 (19.5)	1.3 (21.7)	1.9 (13.6)	2.7 (20.8)
	13	NA	NA	NA	0.1 (1.1)	0.1 (0.7)	0.1 (1.2)	0.0 (0.5)	0.2 (1.2)	0.1 (0.4)
	14	NA	NA	NA	0.1 (1.0)	0.1 (1.0)	0.1 (0.9)	0.1 (1.1)	0.1 (0.7)	0.1 (0.5)
	15	NA	NA	NA	0.2 (3.2)	0.2 (3.2)	0.3 (3.7)	0.2 (3.6)	1.2 (8.4)	3.6 (28.1)
	16	NA	NA	NA	0.9 (17.1)	1.7 (23.5)	0.8 (10.7)	0.8 (13.4)	4.2 (30.6)	2.2 (17.3)
	17	NA	NA	NA	0.0 (0.1)	0.0 (0.1)	0.0 (0.2)	0.0 (0.2)	0.0 (0.1)	0.0 (0.0)
	18	NA	NA	NA	NA	NA	NA	NA	0.0 (0.0)	0.3 (2.0)
	19	NA	NA	NA	NA	NA	NA	NA	0.0 (0.0)	0.0 (0.0)
	20	NA	NA	NA	NA	NA	NA	NA	0.0 (0.0)	0.0 (0.0)
	21	NA	NA	NA	NA	NA	NA	NA	0.0 (0.0)	0.0 (0.0)
	22	NA	NA	NA	NA	NA	NA	NA	0.0 (0.0)	0.0 (0.1)
	23	NA	NA	NA	NA	NA	NA	NA	0.0 (0.0)	0.0 (0.0)
Iceland	1	1.6 (56.9)	2.1 (70.3)	3.3 (78.6)	2.6 (82.1)	1.5 (66.5)	2.0 (73.4)	0.7 (43.8)	0.4 (13.9)	1.8 (38.4)
	2	0.0 (0.2)	0.0 (0.2)	0.0 (0.3)	0.0 (0.4)	0.0 (1.1)	0.0 (0.8)	0.0 (2.3)	0.0 (0.6)	0.0 (0.2)
	3	0.0 (1.1)	0.1 (4.7)	0.2 (5.5)	0.1 (4.4)	0.1 (3.5)	0.1 (3.9)	0.2 (11.3)	0.5 (15.3)	0.7 (14.3)
	4	0.0 (0.6)	0.0 (0.4)	0.0 (0.4)	0.0 (0.2)	0.0 (0.5)	0.0 (0.4)	0.0 (2.7)	0.3 (9.7)	0.3 (7.1)
	5	0.0 (0.2)	0.0 (0.2)	0.0 (0.1)	0.0 (0.2)	0.0 (0.5)	0.0 (0.2)	0.0 (0.4)	0.0 (0.1)	0.0 (0.1)
	6	0.7 (25.8)	0.3 (10.2)	0.3 (7.0)	0.1 (4.2)	0.1 (6.3)	0.2 (6.8)	0.1 (7.3)	0.3 (8.3)	1.0 (22.0)
	7	0.0 (0.0)	0.0 (0.2)	0.0 (0.0)	0.0 (0.0)	0.0 (0.1)	0.0 (0.4)	0.0 (1.7)	0.0 (1.5)	0.2 (5.3)
	8	0.1 (2.6)	0.0 (1.6)	0.1 (1.5)	0.1 (1.7)	0.2 (9.2)	0.1 (2.3)	0.1 (5.9)	0.1 (1.7)	0.1 (1.2)
	9	0.0 (0.7)	0.0 (0.6)	0.0 (0.7)	0.0 (0.6)	0.0 (0.3)	0.0 (0.7)	0.0 (1.9)	0.0 (1.1)	0.0 (0.3)
	10	0.0 (0.2)	0.0 (0.1)	0.0 (0.4)	0.0 (0.1)	0.0 (0.3)	0.0 (0.7)	0.0 (0.9)	0.0 (0.3)	0.0 (0.1)
	11	0.1 (2.9)	0.1 (1.8)	0.1 (1.6)	0.0 (1.1)	0.1 (2.4)	0.0 (1.7)	0.0 (2.2)	0.0 (0.9)	0.2 (3.4)
	12	0.1 (4.2)	0.1 (4.4)	0.1 (3.0)	0.1 (3.8)	0.1 (5.6)	0.2 (6.3)	0.2 (9.9)	0.2 (4.7)	0.1 (2.6)
	13	0.0 (0.2)	0.1 (2.4)	0.0 (0.2)	0.0 (0.2)	0.0 (0.2)	0.0 (0.3)	0.0 (0.3)	0.0 (0.6)	0.0 (0.0)
	14	0.0 (0.0)	0.0 (0.0)	0.0 (0.0)	0.0 (0.2)	0.0 (0.3)	0.0 (0.3)	0.0 (0.4)	0.0 (0.0)	0.0 (0.0)
	15	0.0 (1.2)	0.0 (0.8)	0.0 (0.6)	0.0 (0.6)	0.0 (1.6)	0.0 (1.3)	0.1 (3.3)	1.2 (38.8)	0.2 (4.0)
	16	0.1 (3.2)	0.1 (2.2)	0.0 (0.1)	0.0 (0.2)	0.0 (1.5)	0.0 (0.5)	0.1 (3.8)	0.1 (2.1)	0.0 (0.9)
	17	0.0 (0.0)	0.0 (0.0)	0.0 (0.0)	0.0 (0.0)	0.0 (0.1)	0.0 (0.1)	0.0 (2.0)	0.0 (0.4)	0.0 (0.1)
	18	NA	NA	NA	NA	NA	NA	NA	0.0 (0.0)	0.0 (0.0)
	19	NA	NA	NA	NA	NA	NA	NA	0.0 (0.0)	0.0 (0.0)
	20	NA	NA	NA	NA	NA	NA	NA	0.0 (0.0)	0.0 (0.0)
	21	NA	NA	NA	NA	NA	NA	NA	0.0 (0.0)	0.0 (0.0)
	22	NA	NA	NA	NA	NA	NA	NA	0.0 (0.0)	0.0 (0.0)
	23	NA	NA	NA	NA	NA	NA	NA	0.0 (0.0)	0.0 (0.0)
Ireland	1	24.0 (19.1)	19.0 (15.8)	17.4 (13.3)	15.9 (12.4)	9.5 (8.9)	9.1 (8.4)	12.7 (11.7)	15.5 (15.8)	24.5 (23.0)
	2	1.2 (1.0)	0.7 (0.6)	1.5 (1.2)	6.0 (4.7)	5.9 (5.5)	3.7 (3.4)	3.3 (3.1)	1.1 (1.2)	1.8 (1.7)
	3	6.8 (5.4)	8.5 (7.1)	13.2 (10.1)	20.6 (16.1)	15.6 (14.6)	17.8 (16.4)	21.2 (19.5)	21.6 (22.1)	18.4 (17.3)

	4	1.4 (1.1)	4.8 (4.0)	2.9 (2.2)	3.4 (2.6)	3.9 (3.6)	3.6 (3.4)	2.8 (2.6)	1.5 (1.6)	1.0 (0.9)
	5	1.7 (1.4)	1.1 (0.9)	1.2 (0.9)	1.0 (0.8)	0.3 (0.2)	0.8 (0.8)	0.7 (0.7)	0.9 (0.9)	1.2 (1.1)
	6	4.4 (3.5)	2.9 (2.4)	3.2 (2.5)	2.4 (1.9)	10.0 (9.3)	2.9 (2.7)	3.1 (2.9)	1.4 (1.4)	3.9 (3.7)
	7	0.1 (0.1)	0.4 (0.3)	0.4 (0.3)	0.4 (0.3)	0.3 (0.2)	0.7 (0.6)	0.6 (0.5)	1.7 (1.8)	0.3 (0.3)
	8	6.0 (4.7)	6.0 (4.9)	12.0 (9.2)	10.0 (7.8)	5.2 (4.9)	4.8 (4.4)	3.3 (3.0)	3.3 (3.3)	4.0 (3.8)
	9	3.2 (2.6)	4.6 (3.9)	5.0 (3.8)	4.8 (3.8)	4.9 (4.6)	4.7 (4.3)	4.9 (4.5)	4.8 (4.9)	6.0 (5.7)
	10	2.1 (1.6)	2.3 (1.9)	3.6 (2.8)	2.5 (2.0)	3.1 (2.9)	3.3 (3.0)	3.2 (2.9)	3.0 (3.0)	3.3 (3.1)
	11	31.8 (25.3)	28.6 (23.7)	27.3 (20.9)	21.6 (16.8)	13.9 (13.0)	15.8 (14.6)	13.6 (12.5)	14.2 (14.6)	12.5 (11.8)
	12	37.5 (29.9)	36.6 (30.4)	38.3 (29.3)	33.5 (26.1)	28.8 (26.9)	36.0 (33.3)	34.3 (31.6)	26.1 (26.7)	25.1 (23.6)
	13	1.0 (0.8)	1.0 (0.9)	0.3 (0.2)	1.1 (0.8)	1.6 (1.4)	0.9 (0.8)	0.9 (0.8)	0.2 (0.2)	0.7 (0.6)
	14	0.2 (0.1)	0.2 (0.1)	0.3 (0.2)	1.1 (0.8)	0.8 (0.8)	0.7 (0.6)	0.3 (0.3)	0.1 (0.1)	0.1 (0.1)
	15	1.7 (1.4)	0.8 (0.7)	1.1 (0.8)	0.9 (0.7)	0.9 (0.8)	1.1 (1.0)	1.4 (1.2)	1.3 (1.3)	1.1 (1.0)
	16	2.5 (2.0)	3.0 (2.5)	2.9 (2.2)	3.1 (2.4)	2.3 (2.2)	2.1 (1.9)	1.8 (1.7)	1.0 (1.0)	1.9 (1.8)
	17	0.0 (0.0)	0.0 (0.0)	0.1 (0.0)	0.0 (0.0)	0.4 (0.4)	0.4 (0.3)	0.4 (0.4)	0.1 (0.1)	0.1 (0.1)
	18	NA	NA	NA	NA	NA	NA	NA	0.0 (0.0)	0.4 (0.4)
	19	NA	NA	NA	NA	NA	NA	NA	0.0 (0.0)	0.0 (0.0)
	20	NA	NA	NA	NA	NA	NA	NA	0.0 (0.0)	0.0 (0.0)
	21	NA	NA	NA	NA	NA	NA	NA	0.0 (0.0)	0.0 (0.0)
	22	NA	NA	NA	NA	NA	NA	NA	0.0 (0.0)	0.0 (0.0)
	23	NA	NA	NA	NA	NA	NA	NA	0.0 (0.0)	0.0 (0.0)
Italy	1	42.3 (24.7)	27.8 (21.1)	44.1 (27.7)	33.1 (16.9)	34.8 (16.0)	42.3 (17.7)	57.7 (20.9)	61.9 (20.9)	65.7 (20.1)
	2	15.2 (8.9)	5.0 (3.8)	7.1 (4.5)	5.0 (2.6)	7.1 (3.2)	13.7 (5.7)	35.9 (13.0)	16.7 (5.6)	33.9 (10.4)
	3	5.7 (3.3)	5.9 (4.5)	8.4 (5.3)	13.8 (7.0)	10.6 (4.9)	22.7 (9.5)	21.9 (7.9)	24.6 (8.3)	24.2 (7.4)
	4	2.2 (1.3)	1.7 (1.3)	1.9 (1.2)	1.0 (0.5)	3.1 (1.4)	1.1 (0.4)	2.2 (0.8)	3.0 (1.0)	2.9 (0.9)
	5	2.0 (1.2)	2.8 (2.2)	1.6 (1.0)	3.4 (1.8)	4.7 (2.1)	3.5 (1.4)	2.9 (1.0)	2.6 (0.9)	3.9 (1.2)
	6	7.6 (4.4)	6.6 (5.0)	10.3 (6.5)	8.3 (4.2)	9.4 (4.3)	10.1 (4.2)	7.9 (2.9)	11.2 (3.8)	12.7 (3.9)
	7	0.0 (0.0)	1.3 (1.0)	0.4 (0.3)	0.8 (0.4)	0.3 (0.1)	0.9 (0.4)	2.4 (0.9)	3.4 (1.2)	1.1 (0.3)
	8	8.4 (4.9)	10.0 (7.6)	13.6 (8.5)	12.1 (6.2)	23.4 (10.7)	13.2 (5.5)	53.5 (19.4)	21.8 (7.4)	24.2 (7.4)
	9	4.1 (2.4)	2.0 (1.5)	2.0 (1.3)	13.7 (7.0)	10.6 (4.9)	14.7 (6.2)	1.8 (0.7)	17.3 (5.8)	21.7 (6.6)
	10	0.9 (0.5)	0.6 (0.4)	0.8 (0.5)	5.9 (3.0)	5.8 (2.6)	9.0 (3.8)	1.1 (0.4)	9.9 (3.3)	11.0 (3.4)
	11	15.7 (9.2)	5.6 (4.3)	11.8 (7.4)	30.2 (15.5)	20.0 (9.2)	26.2 (11.0)	2.5 (0.9)	19.9 (6.7)	23.3 (7.1)
	12	38.4 (22.5)	34.4 (26.2)	29.5 (18.6)	32.1 (16.4)	40.0 (18.4)	34.2 (14.3)	39.1 (14.2)	37.5 (12.7)	46.1 (14.1)
	13	5.6 (3.3)	5.2 (4.0)	7.8 (4.9)	7.0 (3.6)	4.4 (2.0)	2.2 (0.9)	3.6 (1.3)	5.3 (1.8)	4.3 (1.3)
	14	0.5 (0.3)	0.2 (0.2)	0.2 (0.1)	0.9 (0.5)	1.3 (0.6)	1.3 (0.5)	0.9 (0.3)	0.8 (0.3)	1.3 (0.4)
	15	13.1 (7.7)	17.0 (12.9)	14.5 (9.1)	21.2 (10.8)	35.4 (16.2)	32.9 (13.7)	20.7 (7.5)	52.3 (17.7)	38.7 (11.8)
	16	9.2 (5.4)	5.2 (4.0)	4.3 (2.7)	6.4 (3.3)	6.5 (3.0)	10.4 (4.3)	20.2 (7.3)	4.7 (1.6)	5.8 (1.8)
	17	0.1 (0.0)	0.2 (0.2)	0.6 (0.3)	0.7 (0.3)	0.6 (0.3)	0.8 (0.3)	1.3 (0.5)	2.0 (0.7)	0.7 (0.2)
	18	NA	NA	NA	NA	NA	NA	NA	0.3 (0.1)	4.2 (1.3)
	19	NA	NA	NA	NA	NA	NA	NA	0.6 (0.2)	0.3 (0.1)
	20	NA	NA	NA	NA	NA	NA	NA	0.0 (0.0)	0.1 (0.0)
	21	NA	NA	NA	NA	NA	NA	NA	0.0 (0.0)	0.1 (0.0)
	22	NA	NA	NA	NA	NA	NA	NA	0.3 (0.1)	1.0 (0.3)
	23	NA	NA	NA	NA	NA	NA	NA	0.0 (0.0)	0.0 (0.0)
Japan	1	59.4 (8.5)	57.9 (6.8)	64.9 (9.1)	71.0 (8.3)	30.3 (3.5)	70.7 (8.0)	53.7 (4.9)	64.5 (6.8)	75.6 (7.4)
	2	47.0 (6.7)	68.3 (8.0)	35.5 (5.0)	59.1 (6.9)	73.5 (8.5)	74.5 (8.4)	56.1 (5.1)	67.6 (7.1)	67.7 (6.6)
	3	11.7 (1.7)	41.6 (4.9)	111.3 (15.5)	65.4 (7.6)	18.9 (2.2)	23.3 (2.6)	39.1 (3.6)	41.4 (4.4)	43.4 (4.2)
	4	3.3 (0.5)	3.1 (0.4)	5.9 (0.8)	6.6 (0.8)	5.7 (0.7)	2.7 (0.3)	5.0 (0.5)	8.4 (0.9)	6.2 (0.6)

	5	3.4 (0.5)	7.0 (0.8)	12.4 (1.7)	3.9 (0.5)	12.3 (1.4)	23.5 (2.6)	11.8 (1.1)	12.5 (1.3)	8.6 (0.8)
	6	53.9 (7.7)	46.5 (5.4)	52.7 (7.3)	57.0 (6.7)	58.2 (6.7)	68.3 (7.7)	55.6 (5.1)	57.5 (6.1)	48.6 (4.8)
	7	7.1 (1.0)	9.1 (1.1)	2.1 (0.3)	2.6 (0.3)	1.8 (0.2)	4.3 (0.5)	3.0 (0.3)	10.6 (1.1)	3.9 (0.4)
	8	112.6 (16.2)	107.4 (12.6)	46.6 (6.5)	59.2 (6.9)	206.9 (23.9)	132.0 (14.9)	163.6 (14.9)	148.8 (15.7)	179.6 (17.6)
	9	35.0 (5.0)	88.2 (10.3)	68.0 (9.5)	92.2 (10.8)	68.1 (7.9)	61.5 (6.9)	115.1 (10.5)	101.6 (10.7)	127.0 (12.4)
	10	26.4 (3.8)	47.8 (5.6)	28.4 (4.0)	59.5 (7.0)	37.6 (4.3)	38.9 (4.4)	73.0 (6.7)	50.0 (5.3)	58.6 (5.7)
	11	95.4 (13.7)	171.3 (20.0)	84.8 (11.8)	173.2 (20.3)	113.0 (13.0)	87.0 (9.8)	125.6 (11.5)	102.5 (10.8)	116.3 (11.4)
	12	154.8 (22.2)	143.0 (16.7)	110.5 (15.4)	102.0 (11.9)	106.2 (12.3)	165.7 (18.6)	227.7 (20.8)	131.4 (13.9)	112.2 (11.0)
	13	7.2 (1.0)	3.4 (0.4)	3.2 (0.4)	9.0 (1.1)	2.2 (0.3)	8.6 (1.0)	35.7 (3.3)	30.1 (3.2)	22.7 (2.2)
	14	0.7 (0.1)	0.3 (0.0)	6.9 (1.0)	3.0 (0.3)	2.8 (0.3)	5.0 (0.6)	2.9 (0.3)	2.4 (0.3)	4.1 (0.4)
	15	68.9 (9.9)	30.3 (3.5)	55.3 (7.7)	64.0 (7.5)	98.9 (11.4)	109.7 (12.3)	122.1 (11.1)	98.7 (10.4)	128.3 (12.6)
	16	8.4 (1.2)	22.5 (2.6)	16.7 (2.3)	17.7 (2.1)	14.9 (1.7)	6.6 (0.7)	4.9 (0.4)	13.2 (1.4)	4.8 (0.5)
	17	1.6 (0.2)	8.1 (1.0)	11.9 (1.7)	10.0 (1.2)	15.0 (1.7)	6.4 (0.7)	1.1 (0.1)	7.2 (0.8)	1.1 (0.1)
	18	NA	NA	NA	NA	NA	NA	NA	0.0 (0.0)	6.7 (0.7)
	19	NA	NA	NA	NA	NA	NA	NA	0.0 (0.0)	0.0 (0.0)
	20	NA	NA	NA	NA	NA	NA	NA	0.0 (0.0)	0.0 (0.0)
	21	NA	NA	NA	NA	NA	NA	NA	0.0 (0.0)	0.1 (0.0)
	22	NA	NA	NA	NA	NA	NA	NA	0.0 (0.0)	5.7 (0.6)
	23	NA	NA	NA	NA	NA	NA	NA	0.0 (0.0)	0.0 (0.0)
Luxembourg	1	7.8 (12.4)	8.0 (12.7)	13.7 (19.3)	15.8 (23.6)	5.2 (8.6)	5.0 (7.8)	4.7 (7.7)	6.0 (9.8)	8.1 (11.9)
	2	7.1 (11.2)	7.7 (12.2)	8.5 (12.0)	4.7 (7.0)	4.1 (6.7)	0.8 (1.3)	2.3 (3.7)	2.3 (3.8)	1.8 (2.6)
	3	3.0 (4.8)	3.0 (4.7)	2.1 (3.0)	1.8 (2.7)	1.0 (1.7)	1.6 (2.5)	2.5 (4.0)	2.4 (3.9)	5.6 (8.1)
	4	1.3 (2.1)	1.7 (2.7)	1.5 (2.1)	1.0 (1.5)	0.8 (1.2)	0.3 (0.4)	0.7 (1.1)	0.7 (1.2)	1.3 (1.8)
	5	1.6 (2.5)	2.9 (4.6)	1.2 (1.7)	1.8 (2.7)	1.9 (3.2)	0.6 (0.9)	0.8 (1.4)	1.3 (2.1)	1.0 (1.4)
	6	4.8 (7.7)	2.4 (3.9)	2.6 (3.6)	3.6 (5.3)	5.0 (8.3)	3.5 (5.5)	4.4 (7.2)	5.3 (8.5)	3.0 (4.5)
	7	0.0 (0.0)	0.5 (0.8)	0.8 (1.1)	0.5 (0.8)	0.7 (1.1)	8.1 (12.8)	0.8 (1.2)	4.0 (6.4)	3.1 (4.5)
	8	5.4 (8.6)	3.7 (5.8)	4.0 (5.6)	2.8 (4.2)	4.3 (7.1)	1.9 (2.9)	2.5 (4.0)	1.9 (3.1)	5.3 (7.7)
	9	0.2 (0.3)	0.6 (0.9)	0.2 (0.3)	1.1 (1.7)	1.1 (1.8)	1.1 (1.8)	1.3 (2.1)	1.3 (2.0)	1.1 (1.7)
	10	0.5 (0.8)	0.2 (0.4)	0.1 (0.2)	0.6 (0.9)	0.7 (1.1)	0.9 (1.5)	1.0 (1.6)	0.7 (1.1)	0.5 (0.7)
	11	8.3 (13.2)	8.3 (13.2)	7.0 (9.8)	7.2 (10.8)	7.0 (11.5)	8.1 (12.8)	8.0 (13.1)	8.6 (14.0)	5.5 (8.1)
	12	13.7 (21.7)	11.5 (18.3)	16.6 (23.4)	10.7 (16.0)	13.6 (22.6)	18.7 (29.5)	20.9 (34.0)	17.8 (29.0)	25.5 (37.3)
	13	0.3 (0.5)	0.4 (0.7)	0.3 (0.4)	0.3 (0.4)	0.2 (0.4)	0.5 (0.7)	0.2 (0.3)	0.4 (0.6)	0.1 (0.1)
	14	0.3 (0.5)	1.2 (2.0)	0.4 (0.6)	1.9 (2.9)	1.5 (2.4)	0.5 (0.8)	2.2 (3.7)	2.4 (3.8)	0.8 (1.2)
	15	5.3 (8.4)	5.8 (9.2)	8.1 (11.3)	7.8 (11.7)	9.4 (15.6)	9.9 (15.7)	5.8 (9.4)	4.6 (7.5)	4.3 (6.3)
	16	3.3 (5.2)	5.1 (8.0)	4.0 (5.6)	5.0 (7.5)	3.3 (5.4)	1.4 (2.2)	2.5 (4.1)	1.7 (2.8)	0.8 (1.2)
	17	0.0 (0.0)	0.0 (0.0)	0.0 (0.0)	0.2 (0.3)	0.8 (1.3)	0.6 (0.9)	1.0 (1.6)	0.2 (0.3)	0.1 (0.1)
	18	NA	NA	NA	NA	NA	NA	NA	0.0 (0.0)	0.5 (0.8)
	19	NA	NA	NA	NA	NA	NA	NA	0.0 (0.0)	0.0 (0.0)
	20	NA	NA	NA	NA	NA	NA	NA	0.0 (0.0)	0.0 (0.0)
	21	NA	NA	NA	NA	NA	NA	NA	0.0 (0.0)	0.0 (0.0)
	22	NA	NA	NA	NA	NA	NA	NA	0.0 (0.0)	0.0 (0.0)
	23	NA	NA	NA	NA	NA	NA	NA	0.0 (0.0)	0.0 (0.0)
Netherlands	1	131.2 (23.6)	90.0 (18.4)	71.0 (13.3)	59.6 (11.8)	60.8 (12.4)	62.6 (11.0)	77.8 (14.8)	67.1 (11.0)	65.2 (12.5)
	2	1.4 (0.2)	2.8 (0.6)	3.0 (0.6)	1.2 (0.2)	2.6 (0.5)	4.6 (0.8)	7.7 (1.5)	3.7 (0.6)	5.1 (1.0)
	3	8.3 (1.5)	9.0 (1.8)	27.1 (5.1)	31.2 (6.2)	43.4 (8.9)	61.2 (10.8)	28.8 (5.5)	73.1 (12.0)	40.2 (7.7)
	4	2.0 (0.4)	1.3 (0.3)	1.8 (0.3)	0.6 (0.1)	0.9 (0.2)	1.0 (0.2)	1.9 (0.4)	1.0 (0.2)	1.2 (0.2)
	5	3.7 (0.7)	1.3 (0.3)	0.7 (0.1)	1.1 (0.2)	1.5 (0.3)	1.2 (0.2)	1.3 (0.2)	0.7 (0.1)	1.5 (0.3)

	6	105.2 (18.9)	106.8 (21.9)	134.7 (25.2)	134.6 (26.7)	140.0 (28.6)	200.5 (35.2)	171.8 (32.8)	186.4 (30.5)	182.8 (34.9)
	7	3.2 (0.6)	8.8 (1.8)	15.1 (2.8)	9.1 (1.8)	10.2 (2.1)	25.7 (4.5)	31.9 (6.1)	30.7 (5.0)	32.4 (6.2)
	8	16.9 (3.0)	9.7 (2.0)	12.0 (2.2)	9.9 (2.0)	27.7 (5.7)	18.2 (3.2)	22.1 (4.2)	24.5 (4.0)	28.2 (5.4)
	9	26.3 (4.7)	16.6 (3.4)	24.8 (4.6)	28.2 (5.6)	20.7 (4.2)	21.4 (3.8)	22.0 (4.2)	22.3 (3.6)	26.7 (5.1)
	10	20.2 (3.6)	9.7 (2.0)	20.5 (3.8)	17.4 (3.4)	11.2 (2.3)	12.9 (2.3)	11.9 (2.3)	10.9 (1.8)	17.3 (3.3)
	11	124.5 (22.4)	83.7 (17.2)	84.7 (15.8)	79.1 (15.7)	75.0 (15.3)	66.9 (11.7)	57.5 (11.0)	73.7 (12.0)	41.9 (8.0)
	12	54.8 (9.9)	60.7 (12.4)	42.9 (8.0)	53.7 (10.6)	51.0 (10.4)	28.9 (5.1)	22.3 (4.2)	42.5 (6.9)	31.5 (6.0)
	13	4.3 (0.8)	3.5 (0.7)	0.8 (0.1)	0.8 (0.2)	0.7 (0.1)	1.3 (0.2)	0.6 (0.1)	0.9 (0.2)	0.4 (0.1)
	14	18.6 (3.3)	10.4 (2.1)	17.4 (3.2)	21.7 (4.3)	5.6 (1.1)	2.9 (0.5)	1.3 (0.2)	0.7 (0.1)	1.4 (0.3)
	15	6.3 (1.1)	3.1 (0.6)	3.1 (0.6)	2.5 (0.5)	3.7 (0.8)	4.9 (0.9)	2.2 (0.4)	5.6 (0.9)	4.5 (0.9)
	16	28.5 (5.1)	69.9 (14.3)	74.5 (13.9)	51.0 (10.1)	23.2 (4.7)	43.5 (7.6)	56.0 (10.7)	61.1 (10.0)	39.1 (7.5)
	17	0.9 (0.2)	0.9 (0.2)	0.9 (0.2)	2.6 (0.5)	10.8 (2.2)	11.4 (2.0)	7.1 (1.4)	6.4 (1.0)	2.2 (0.4)
	18	NA	NA	NA	NA	NA	NA	NA	0.0 (0.0)	2.1 (0.4)
	19	NA	NA	NA	NA	NA	NA	NA	0.0 (0.0)	0.0 (0.0)
	20	NA	NA	NA	NA	NA	NA	NA	0.0 (0.0)	0.0 (0.0)
	21	NA	NA	NA	NA	NA	NA	NA	0.0 (0.0)	0.0 (0.0)
	22	NA	NA	NA	NA	NA	NA	NA	0.0 (0.0)	0.1 (0.0)
	23	NA	NA	NA	NA	NA	NA	NA	0.0 (0.0)	0.0 (0.0)
New Zealand	1	7.2 (19.7)	8.4 (23.7)	2.9 (9.0)	5.2 (18.2)	6.5 (29.2)	1.9 (9.9)	1.5 (7.2)	2.9 (11.7)	3.8 (15.9)
	2	0.0 (0.1)	0.0 (0.0)	4.6 (14.2)	0.1 (0.2)	0.1 (0.3)	0.0 (0.2)	0.1 (0.5)	0.8 (3.0)	1.6 (6.6)
	3	1.0 (2.6)	0.6 (1.6)	0.6 (1.9)	0.5 (1.7)	0.5 (2.4)	0.6 (2.9)	0.6 (3.1)	2.6 (10.5)	0.7 (2.8)
	4	1.4 (3.7)	0.6 (1.6)	0.1 (0.4)	0.5 (1.6)	0.4 (1.6)	0.3 (1.6)	1.3 (6.6)	0.2 (0.6)	0.7 (2.7)
	5	0.4 (1.0)	0.3 (0.9)	0.6 (1.9)	1.3 (4.7)	0.1 (0.6)	1.8 (9.1)	1.3 (6.4)	1.1 (4.5)	1.0 (4.1)
	6	8.8 (24.0)	6.1 (17.2)	6.7 (20.7)	4.5 (15.6)	3.8 (17.4)	3.5 (18.0)	3.3 (16.2)	3.0 (11.9)	2.7 (11.1)
	7	0.2 (0.7)	0.5 (1.3)	0.4 (1.1)	0.4 (1.5)	0.8 (3.8)	0.7 (3.6)	0.7 (3.4)	1.1 (4.3)	1.1 (4.4)
	8	3.3 (9.1)	2.1 (5.8)	1.7 (5.2)	2.3 (7.9)	0.8 (3.6)	0.6 (2.9)	3.7 (18.3)	2.8 (11.2)	5.2 (21.6)
	9	0.3 (0.9)	0.0 (0.1)	0.2 (0.5)	0.1 (0.3)	0.0 (0.1)	0.1 (0.3)	0.2 (1.0)	0.2 (0.7)	0.2 (1.0)
	10	0.0 (0.1)	0.0 (0.0)	0.1 (0.2)	0.0 (0.1)	0.0 (0.1)	0.1 (0.3)	0.1 (0.5)	0.1 (0.4)	0.1 (0.4)
	11	5.2 (14.3)	3.3 (9.3)	2.2 (6.6)	0.7 (2.4)	1.1 (4.9)	1.1 (5.7)	0.9 (4.4)	0.9 (3.7)	0.4 (1.5)
	12	4.6 (12.6)	6.5 (18.4)	3.6 (11.2)	1.7 (5.8)	1.0 (4.3)	1.2 (5.9)	1.3 (6.6)	2.2 (8.6)	1.2 (5.1)
	13	0.0 (0.1)	0.1 (0.2)	0.1 (0.2)	0.1 (0.2)	0.0 (0.1)	1.1 (5.7)	1.0 (5.0)	0.6 (2.3)	0.7 (2.9)
	14	0.0 (0.0)	0.0 (0.0)	0.0 (0.0)	0.0 (0.2)	0.0 (0.2)	0.1 (0.3)	0.1 (0.3)	0.1 (0.2)	0.1 (0.4)
	15	2.2 (5.9)	4.0 (11.4)	5.8 (17.7)	8.5 (29.5)	5.0 (22.4)	5.2 (26.6)	3.0 (14.5)	2.4 (9.4)	3.5 (14.4)
	16	1.8 (5.0)	3.0 (8.4)	3.0 (9.1)	2.9 (10.2)	1.5 (6.8)	0.9 (4.7)	0.8 (3.8)	1.0 (4.1)	0.5 (2.1)
	17	0.0 (0.0)	0.0 (0.0)	0.0 (0.0)	0.0 (0.0)	0.5 (2.1)	0.4 (2.2)	0.4 (2.1)	0.1 (0.4)	0.0 (0.2)
	18	NA	NA	NA	NA	NA	NA	NA	0.1 (0.6)	0.2 (1.0)
	19	NA	NA	NA	NA	NA	NA	NA	0.0 (0.0)	0.0 (0.0)
	20	NA	NA	NA	NA	NA	NA	NA	0.5 (1.9)	0.0 (0.0)
	21	NA	NA	NA	NA	NA	NA	NA	0.0 (0.1)	0.0 (0.0)
	22	NA	NA	NA	NA	NA	NA	NA	2.5 (9.9)	0.4 (1.8)
	23	NA	NA	NA	NA	NA	NA	NA	0.0 (0.0)	0.0 (0.0)
Norway	1	76.4 (20.2)	100.7 (25.7)	106.8 (22.5)	118.3 (23.4)	131.2 (25.1)	211.1 (41.5)	215.8 (40.1)	193.9 (37.7)	191.7 (38.2)
	2	4.4 (1.2)	2.8 (0.7)	1.9 (0.4)	4.8 (0.9)	5.9 (1.1)	3.7 (0.7)	6.9 (1.3)	4.2 (0.8)	2.7 (0.5)
	3	3.9 (1.0)	10.1 (2.6)	4.9 (1.0)	4.7 (0.9)	6.1 (1.2)	5.7 (1.1)	6.0 (1.1)	10.4 (2.0)	6.8 (1.4)
	4	4.9 (1.3)	4.5 (1.2)	6.0 (1.3)	3.2 (0.6)	4.2 (0.8)	4.0 (0.8)	7.8 (1.4)	4.5 (0.9)	3.7 (0.7)
	5	2.6 (0.7)	2.0 (0.5)	6.0 (1.3)	9.5 (1.9)	12.8 (2.4)	4.6 (0.9)	10.1 (1.9)	4.1 (0.8)	2.3 (0.5)
	6	48.8 (12.9)	40.6 (10.4)	86.8 (18.3)	80.8 (16.0)	78.9 (15.1)	16.6 (3.3)	34.8 (6.5)	35.4 (6.9)	30.9 (6.1)

	7	3.2 (0.8)	4.2 (1.1)	18.3 (3.9)	19.5 (3.9)	6.3 (1.2)	10.1 (2.0)	6.5 (1.2)	17.8 (3.5)	19.3 (3.8)
	8	29.1 (7.7)	12.9 (3.3)	14.2 (3.0)	41.8 (8.3)	54.4 (10.4)	49.7 (9.8)	68.3 (12.7)	71.0 (13.8)	72.5 (14.4)
	9	15.5 (4.1)	17.7 (4.5)	15.4 (3.3)	18.7 (3.7)	22.3 (4.3)	22.9 (4.5)	23.6 (4.4)	25.1 (4.9)	27.9 (5.6)
	10	11.6 (3.1)	10.2 (2.6)	12.9 (2.7)	11.3 (2.2)	14.4 (2.8)	15.6 (3.1)	15.3 (2.8)	15.0 (2.9)	14.6 (2.9)
	11	82.1 (21.7)	75.6 (19.3)	68.6 (14.5)	62.1 (12.3)	70.4 (13.5)	59.4 (11.7)	47.0 (8.7)	46.8 (9.1)	33.8 (6.7)
	12	41.4 (10.9)	51.0 (13.0)	54.2 (11.4)	56.2 (11.1)	53.9 (10.3)	53.6 (10.5)	48.6 (9.0)	43.1 (8.4)	63.1 (12.6)
	13	7.9 (2.1)	6.4 (1.6)	5.4 (1.1)	6.4 (1.3)	4.8 (0.9)	7.2 (1.4)	3.7 (0.7)	2.9 (0.6)	3.3 (0.7)
	14	11.8 (3.1)	9.8 (2.5)	20.7 (4.4)	12.3 (2.4)	14.0 (2.7)	18.9 (3.7)	17.0 (3.2)	13.7 (2.7)	9.6 (1.9)
	15	14.4 (3.8)	15.1 (3.9)	16.7 (3.5)	18.0 (3.5)	16.2 (3.1)	8.2 (1.6)	9.8 (1.8)	7.4 (1.4)	7.2 (1.4)
	16	19.9 (5.2)	27.5 (7.0)	35.4 (7.5)	38.2 (7.5)	20.3 (3.9)	12.0 (2.4)	10.3 (1.9)	14.2 (2.7)	8.7 (1.7)
	17	1.2 (0.3)	0.1 (0.0)	0.6 (0.1)	0.6 (0.1)	6.2 (1.2)	5.1 (1.0)	7.3 (1.4)	2.1 (0.4)	1.6 (0.3)
	18	NA	NA	NA	NA	NA	NA	NA	1.1 (0.2)	1.4 (0.3)
	19	NA	NA	NA	NA	NA	NA	NA	0.0 (0.0)	0.0 (0.0)
	20	NA	NA	NA	NA	NA	NA	NA	1.5 (0.3)	0.2 (0.0)
	21	NA	NA	NA	NA	NA	NA	NA	0.7 (0.1)	0.6 (0.1)
	22	NA	NA	NA	NA	NA	NA	NA	0.1 (0.0)	0.2 (0.0)
	23	NA	NA	NA	NA	NA	NA	NA	0.0 (0.0)	0.1 (0.0)
Poland	1	NA	NA	5.6 (36.8)	4.0 (28.9)	5.0 (28.3)	6.9 (28.1)	5.5 (22.5)	4.8 (25.9)	5.5 (15.3)
	2	NA	NA	0.3 (1.7)	0.2 (1.3)	0.3 (1.9)	2.2 (8.9)	5.3 (21.6)	2.0 (10.8)	3.3 (9.1)
	3	NA	NA	1.2 (7.8)	1.9 (13.6)	1.3 (7.6)	3.3 (13.4)	2.5 (10.0)	2.3 (12.5)	3.3 (9.2)
	4	NA	NA	0.1 (0.9)	0.1 (0.8)	0.1 (0.7)	0.1 (0.4)	0.1 (0.4)	0.0 (0.3)	0.1 (0.3)
	5	NA	NA	0.2 (1.4)	0.2 (1.5)	0.4 (2.3)	0.4 (1.5)	0.4 (1.5)	0.4 (2.4)	0.7 (1.8)
	6	NA	NA	1.0 (6.6)	1.0 (7.0)	1.4 (7.6)	1.6 (6.4)	0.6 (2.6)	0.5 (2.8)	0.8 (2.2)
	7	NA	NA	0.1 (0.4)	0.1 (0.9)	0.0 (0.2)	0.1 (0.3)	0.4 (1.8)	0.1 (0.5)	0.1 (0.2)
	8	NA	NA	1.6 (10.8)	1.0 (7.2)	1.9 (10.7)	1.3 (5.4)	0.8 (3.2)	1.0 (5.2)	1.1 (3.0)
	9	NA	NA	0.0 (0.2)	0.1 (0.6)	0.1 (0.5)	0.2 (0.6)	0.1 (0.4)	0.1 (0.5)	0.2 (0.6)
	10	NA	NA	0.0 (0.1)	0.0 (0.3)	0.1 (0.5)	0.2 (0.7)	0.1 (0.3)	0.1 (0.4)	0.3 (0.8)
	11	NA	NA	0.5 (3.2)	0.3 (2.1)	0.4 (2.5)	0.3 (1.3)	0.2 (0.7)	0.2 (1.0)	0.3 (0.7)
	12	NA	NA	2.8 (18.5)	2.9 (20.9)	4.1 (23.2)	4.8 (19.5)	4.9 (20.1)	4.9 (26.8)	6.0 (16.9)
	13	NA	NA	0.2 (1.2)	0.2 (1.4)	0.2 (1.0)	0.3 (1.3)	0.1 (0.3)	0.4 (2.2)	0.6 (1.7)
	14	NA	NA	0.0 (0.2)	0.2 (1.3)	0.2 (1.3)	0.2 (0.9)	0.2 (0.7)	0.2 (1.3)	0.3 (1.0)
	15	NA	NA	0.8 (5.3)	0.7 (4.9)	1.0 (5.6)	1.1 (4.4)	0.7 (2.7)	0.9 (4.9)	11.8 (33.2)
	16	NA	NA	0.7 (4.7)	1.0 (7.3)	1.1 (6.0)	1.6 (6.8)	2.5 (10.2)	0.3 (1.5)	0.2 (0.6)
	17	NA	NA	0.0 (0.2)	0.0 (0.0)	0.0 (0.1)	0.0 (0.1)	0.2 (0.9)	0.2 (0.9)	0.1 (0.3)
	18	NA	NA	NA	NA	NA	NA	NA	0.0 (0.1)	1.0 (2.9)
	19	NA	NA	NA	NA	NA	NA	NA	0.0 (0.0)	0.0 (0.0)
	20	NA	NA	NA	NA	NA	NA	NA	0.0 (0.0)	0.0 (0.0)
	21	NA	NA	NA	NA	NA	NA	NA	0.0 (0.0)	0.0 (0.0)
	22	NA	NA	NA	NA	NA	NA	NA	0.0 (0.0)	0.0 (0.1)
	23	NA	NA	NA	NA	NA	NA	NA	0.0 (0.0)	0.0 (0.0)
Portugal	1	5.0 (18.7)	3.2 (16.6)	4.5 (17.4)	3.2 (13.6)	3.6 (11.2)	4.1 (17.9)	4.1 (21.2)	3.5 (20.8)	3.6 (15.5)
	2	0.3 (1.2)	0.1 (0.4)	0.1 (0.3)	0.0 (0.2)	0.1 (0.3)	0.8 (3.4)	2.5 (12.9)	1.3 (8.0)	1.3 (5.6)
	3	0.5 (1.9)	0.4 (2.1)	0.7 (2.7)	1.0 (4.3)	0.6 (2.0)	1.3 (5.8)	1.7 (8.6)	1.1 (6.8)	1.7 (7.5)
	4	0.1 (0.3)	0.1 (0.3)	0.1 (0.6)	0.0 (0.2)	0.0 (0.1)	0.0 (0.1)	0.1 (0.4)	0.0 (0.2)	0.0 (0.2)
	5	0.1 (0.5)	0.1 (0.3)	0.2 (0.7)	0.0 (0.1)	0.1 (0.4)	0.1 (0.5)	0.1 (0.4)	0.1 (0.4)	0.2 (1.0)
	6	1.0 (3.8)	0.5 (2.5)	0.9 (3.7)	0.7 (3.1)	0.9 (2.9)	1.2 (5.1)	1.0 (5.2)	1.2 (7.4)	1.0 (4.4)
	7	0.0 (0.0)	0.1 (0.7)	0.0 (0.1)	0.1 (0.3)	0.0 (0.0)	0.0 (0.0)	0.2 (1.2)	0.1 (0.6)	0.1 (0.2)

	8	1.0 (3.8)	0.2 (1.3)	1.4 (5.3)	0.5 (2.1)	4.3 (13.4)	0.6 (2.6)	0.8 (4.1)	0.5 (3.0)	0.6 (2.6)
	9	0.5 (1.8)	0.1 (0.5)	0.1 (0.3)	0.1 (0.2)	0.1 (0.2)	0.1 (0.3)	0.2 (1.1)	0.1 (0.8)	0.2 (0.9)
	10	0.2 (0.9)	0.0 (0.1)	0.0 (0.1)	0.0 (0.0)	0.0 (0.1)	0.1 (0.2)	0.1 (0.5)	0.1 (0.4)	0.1 (0.5)
	11	1.6 (6.1)	0.3 (1.6)	0.3 (1.3)	0.1 (0.4)	0.2 (0.7)	0.3 (1.1)	0.3 (1.4)	0.3 (1.6)	0.2 (0.9)
	12	2.3 (8.4)	0.8 (4.3)	2.9 (11.4)	0.9 (3.8)	2.0 (6.2)	2.2 (9.5)	3.1 (15.9)	2.5 (15.0)	2.8 (12.2)
	13	0.5 (1.9)	0.3 (1.7)	0.3 (1.3)	0.1 (0.5)	0.1 (0.3)	0.1 (0.6)	0.1 (0.4)	0.1 (0.9)	0.1 (0.5)
	14	1.1 (4.2)	0.6 (3.3)	0.6 (2.4)	0.6 (2.6)	0.7 (2.2)	0.7 (3.1)	0.5 (2.8)	0.1 (0.6)	0.3 (1.4)
	15	11.4 (42.5)	11.9 (61.8)	12.8 (50.1)	15.5 (66.2)	18.8 (58.6)	10.6 (46.4)	3.0 (15.7)	5.3 (31.6)	10.1 (44.1)
	16	0.9 (3.5)	0.5 (2.5)	0.3 (1.3)	0.5 (2.2)	0.5 (1.5)	0.7 (3.1)	1.4 (7.4)	0.2 (1.3)	0.1 (0.5)
	17	0.1 (0.4)	0.0 (0.0)	0.2 (0.9)	0.0 (0.2)	0.0 (0.0)	0.1 (0.4)	0.1 (0.7)	0.1 (0.4)	0.0 (0.1)
	18	NA	NA	NA	NA	NA	NA	NA	0.0 (0.1)	0.4 (1.9)
	19	NA	NA	NA	NA	NA	NA	NA	0.0 (0.0)	0.0 (0.0)
	20	NA	NA	NA	NA	NA	NA	NA	0.0 (0.0)	0.0 (0.1)
	21	NA	NA	NA	NA	NA	NA	NA	0.0 (0.0)	0.0 (0.0)
	22	NA	NA	NA	NA	NA	NA	NA	0.0 (0.0)	0.0 (0.2)
	23	NA	NA	NA	NA	NA	NA	NA	0.0 (0.0)	0.0 (0.0)
Slovak Republic	1	NA	NA	1.2 (36.8)	0.8 (26.5)	1.0 (26.4)	1.4 (32.2)	2.1 (31.3)	2.1 (42.2)	1.1 (23.1)
	2	NA	NA	0.2 (6.7)	0.0 (0.9)	0.1 (3.7)	0.3 (7.1)	1.3 (20.2)	0.3 (6.5)	0.6 (12.8)
	3	NA	NA	0.2 (5.2)	0.4 (12.3)	0.4 (9.1)	0.5 (12.5)	0.8 (12.9)	0.5 (10.0)	0.6 (12.3)
	4	NA	NA	0.1 (1.7)	0.1 (2.2)	0.0 (0.8)	0.0 (0.3)	0.0 (0.2)	0.0 (0.4)	0.0 (0.2)
	5	NA	NA	0.0 (0.8)	0.0 (1.3)	0.1 (1.5)	0.2 (4.1)	0.0 (0.5)	0.0 (0.7)	0.1 (1.9)
	6	NA	NA	0.2 (4.9)	0.3 (8.8)	0.2 (6.1)	0.2 (4.2)	0.1 (1.3)	0.3 (5.8)	0.2 (3.1)
	7	NA	NA	0.0 (0.4)	0.0 (0.8)	0.0 (0.2)	0.0 (0.3)	0.1 (1.3)	0.0 (0.7)	0.0 (0.3)
	8	NA	NA	0.3 (8.7)	0.2 (6.7)	0.5 (13.3)	0.2 (4.5)	0.4 (6.6)	0.2 (4.1)	0.2 (4.6)
	9	NA	NA	0.0 (0.3)	0.0 (0.7)	0.0 (0.5)	0.0 (0.3)	0.0 (0.2)	0.0 (0.8)	0.0 (0.6)
	10	NA	NA	0.0 (0.1)	0.0 (0.3)	0.0 (0.5)	0.0 (0.3)	0.0 (0.2)	0.0 (0.4)	0.0 (0.4)
	11	NA	NA	0.0 (1.5)	0.0 (1.5)	0.1 (3.0)	0.0 (0.9)	0.0 (0.5)	0.0 (0.6)	0.0 (0.6)
	12	NA	NA	0.5 (15.7)	0.6 (19.3)	0.8 (21.2)	0.8 (17.3)	0.9 (14.0)	0.9 (18.0)	1.2 (25.6)
	13	NA	NA	0.0 (1.0)	0.0 (1.2)	0.0 (0.7)	0.1 (1.3)	0.0 (0.3)	0.2 (3.5)	0.0 (0.8)
	14	NA	NA	0.1 (2.9)	0.1 (3.4)	0.1 (1.4)	0.0 (1.0)	0.0 (0.5)	0.0 (0.8)	0.1 (1.1)
	15	NA	NA	0.3 (9.3)	0.2 (7.8)	0.2 (5.6)	0.3 (7.2)	0.2 (2.7)	0.2 (4.0)	0.4 (7.8)
	16	NA	NA	0.1 (4.2)	0.2 (6.4)	0.2 (5.8)	0.3 (6.4)	0.5 (7.3)	0.1 (1.1)	0.1 (1.1)
	17	NA	NA	0.0 (0.0)	0.0 (0.0)	0.0 (0.1)	0.0 (0.1)	0.0 (0.2)	0.0 (0.2)	0.0 (0.1)
	18	NA	NA	NA	NA	NA	NA	NA	0.0 (0.1)	0.2 (3.6)
	19	NA	NA	NA	NA	NA	NA	NA	0.0 (0.0)	0.0 (0.0)
	20	NA	NA	NA	NA	NA	NA	NA	0.0 (0.0)	0.0 (0.0)
	21	NA	NA	NA	NA	NA	NA	NA	0.0 (0.0)	0.0 (0.0)
	22	NA	NA	NA	NA	NA	NA	NA	0.0 (0.0)	0.0 (0.1)
	23	NA	NA	NA	NA	NA	NA	NA	0.0 (0.0)	0.0 (0.0)
Slovenia	1	0.6 (28.5)	0.4 (14.3)	0.7 (24.9)	0.4 (26.6)	0.5 (26.0)	0.7 (27.7)	0.7 (21.6)	0.5 (26.4)	0.6 (23.2)
	2	0.0 (1.3)	0.0 (1.1)	0.0 (1.1)	0.0 (0.9)	0.1 (4.6)	0.2 (7.1)	0.6 (18.3)	0.2 (9.4)	0.3 (12.1)
	3	0.1 (4.1)	0.1 (3.1)	0.1 (4.0)	0.2 (12.7)	0.2 (7.3)	0.3 (12.8)	0.3 (10.1)	0.3 (13.5)	0.3 (13.5)
	4	0.0 (2.0)	0.0 (1.0)	0.0 (0.6)	0.0 (0.6)	0.0 (0.4)	0.0 (0.4)	0.0 (0.3)	0.0 (0.4)	0.0 (0.3)
	5	0.0 (1.8)	0.0 (1.0)	0.0 (0.7)	0.1 (3.8)	0.0 (1.5)	0.0 (0.8)	0.0 (0.7)	0.0 (0.7)	0.0 (2.1)
	6	0.1 (3.1)	0.1 (2.8)	0.1 (3.9)	0.1 (6.1)	0.2 (7.7)	0.2 (6.6)	0.1 (3.5)	0.1 (4.0)	0.1 (3.8)
	7	0.0 (0.0)	0.0 (0.9)	0.0 (0.2)	0.0 (0.8)	0.0 (0.2)	0.0 (0.3)	0.0 (1.6)	0.0 (0.9)	0.0 (0.4)
	8	0.2 (8.1)	0.2 (6.2)	0.2 (8.0)	0.1 (6.9)	0.2 (10.6)	0.1 (4.8)	0.1 (3.9)	0.1 (5.1)	0.1 (4.1)

	9	0.0 (1.2)	0.0 (1.0)	0.0 (0.8)	0.0 (1.1)	0.0 (0.5)	0.0 (0.6)	0.0 (0.7)	0.0 (0.9)	0.0 (0.7)
	10	0.0 (0.5)	0.0 (0.2)	0.0 (0.3)	0.0 (0.4)	0.0 (0.5)	0.0 (0.6)	0.0 (0.4)	0.0 (0.4)	0.0 (0.4)
	11	0.1 (6.0)	0.1 (2.5)	0.1 (2.1)	0.0 (1.8)	0.0 (2.0)	0.0 (1.2)	0.0 (0.7)	0.0 (0.6)	0.0 (0.7)
	12	0.5 (20.3)	0.6 (20.5)	0.4 (14.3)	0.4 (21.7)	0.5 (21.9)	0.5 (19.1)	0.6 (19.9)	0.5 (23.7)	0.6 (24.0)
	13	0.1 (2.6)	0.3 (8.7)	0.1 (4.2)	0.0 (1.4)	0.0 (1.1)	0.1 (4.0)	0.0 (0.3)	0.0 (2.6)	0.1 (4.7)
	14	0.0 (0.4)	0.0 (0.1)	0.0 (0.1)	0.0 (1.2)	0.0 (1.3)	0.0 (0.8)	0.0 (0.7)	0.0 (0.8)	0.0 (1.0)
	15	0.3 (12.8)	1.1 (33.5)	0.8 (32.0)	0.1 (7.6)	0.2 (8.7)	0.2 (6.5)	0.2 (7.9)	0.2 (8.5)	0.1 (3.7)
	16	0.2 (7.3)	0.1 (3.1)	0.1 (2.8)	0.1 (6.5)	0.1 (5.8)	0.2 (6.3)	0.3 (9.2)	0.0 (1.6)	0.0 (1.0)
	17	0.0 (0.0)	0.0 (0.0)	0.0 (0.0)	0.0 (0.0)	0.0 (0.1)	0.0 (0.1)	0.0 (0.3)	0.0 (0.3)	0.0 (0.1)
	18	NA	NA	NA	NA	NA	NA	NA	0.0 (0.1)	0.1 (3.9)
	19	NA	NA	NA	NA	NA	NA	NA	0.0 (0.0)	0.0 (0.0)
	20	NA	NA	NA	NA	NA	NA	NA	0.0 (0.0)	0.0 (0.0)
	21	NA	NA	NA	NA	NA	NA	NA	0.0 (0.0)	0.0 (0.0)
	22	NA	NA	NA	NA	NA	NA	NA	0.0 (0.0)	0.0 (0.2)
	23	NA	NA	NA	NA	NA	NA	NA	0.0 (0.0)	0.0 (0.0)
South Korea	1	10.9 (7.8)	12.5 (6.9)	16.2 (8.1)	29.1 (14.7)	34.7 (16.6)	29.5 (12.0)	25.8 (10.7)	26.3 (11.5)	26.4 (11.7)
	2	54.9 (39.4)	75.7 (41.9)	66.5 (33.1)	41.6 (21.1)	45.2 (21.6)	66.0 (26.7)	37.6 (15.5)	35.3 (15.4)	34.2 (15.1)
	3	2.5 (1.8)	2.7 (1.5)	2.9 (1.5)	2.8 (1.4)	5.4 (2.6)	10.8 (4.4)	8.8 (3.6)	10.6 (4.7)	6.3 (2.8)
	4	0.5 (0.4)	1.4 (0.8)	2.5 (1.2)	1.7 (0.8)	2.2 (1.1)	4.0 (1.6)	4.6 (1.9)	4.8 (2.1)	5.2 (2.3)
	5	0.4 (0.3)	1.2 (0.7)	2.3 (1.1)	1.7 (0.9)	11.9 (5.7)	7.3 (2.9)	6.7 (2.8)	4.2 (1.8)	2.6 (1.1)
	6	3.3 (2.3)	5.9 (3.3)	7.8 (3.9)	17.1 (8.7)	15.4 (7.3)	14.3 (5.8)	21.6 (8.9)	15.1 (6.6)	9.0 (3.9)
	7	0.1 (0.1)	0.4 (0.2)	0.4 (0.2)	0.0 (0.0)	0.1 (0.0)	0.3 (0.1)	0.1 (0.1)	2.0 (0.9)	3.0 (1.3)
	8	9.7 (6.9)	10.1 (5.6)	9.8 (4.9)	8.5 (4.3)	11.5 (5.5)	9.8 (4.0)	17.0 (7.0)	19.2 (8.4)	19.2 (8.5)
	9	2.3 (1.6)	2.6 (1.4)	3.5 (1.8)	3.4 (1.7)	3.5 (1.7)	2.9 (1.2)	2.5 (1.0)	2.7 (1.2)	2.8 (1.2)
	10	2.0 (1.4)	2.8 (1.5)	3.4 (1.7)	1.8 (0.9)	3.4 (1.6)	2.7 (1.1)	1.6 (0.7)	2.2 (0.9)	3.1 (1.4)
	11	5.5 (4.0)	4.3 (2.4)	5.2 (2.6)	4.3 (2.2)	4.0 (1.9)	3.9 (1.6)	4.0 (1.6)	2.6 (1.1)	2.7 (1.2)
	12	14.4 (10.3)	17.6 (9.7)	21.5 (10.7)	21.7 (11.0)	31.2 (14.9)	28.9 (11.7)	31.4 (12.9)	28.5 (12.5)	25.3 (11.2)
	13	4.6 (3.3)	7.5 (4.2)	11.9 (5.9)	20.0 (10.1)	7.7 (3.7)	20.6 (8.4)	15.4 (6.4)	6.8 (3.0)	6.9 (3.0)
	14	0.2 (0.1)	0.3 (0.1)	0.1 (0.1)	0.8 (0.4)	1.2 (0.6)	0.7 (0.3)	0.6 (0.2)	5.7 (2.5)	4.9 (2.2)
	15	27.1 (19.4)	33.8 (18.7)	44.0 (22.0)	41.4 (20.9)	25.6 (12.2)	41.7 (16.9)	62.5 (25.8)	60.3 (26.4)	74.2 (32.7)
	16	0.1 (0.1)	0.4 (0.2)	0.2 (0.1)	0.4 (0.2)	2.8 (1.4)	0.5 (0.2)	0.6 (0.3)	0.3 (0.1)	0.3 (0.1)
	17	1.0 (0.7)	1.4 (0.8)	2.3 (1.2)	1.1 (0.6)	3.7 (1.8)	2.9 (1.2)	1.4 (0.6)	1.7 (0.8)	0.1 (0.0)
	18	NA	NA	NA	NA	NA	NA	NA	0.0 (0.0)	0.7 (0.3)
	19	NA	NA	NA	NA	NA	NA	NA	0.2 (0.1)	0.0 (0.0)
	20	NA	NA	NA	NA	NA	NA	NA	0.0 (0.0)	0.0 (0.0)
	21	NA	NA	NA	NA	NA	NA	NA	0.0 (0.0)	0.0 (0.0)
	22	NA	NA	NA	NA	NA	NA	NA	0.0 (0.0)	0.1 (0.0)
	23	NA	NA	NA	NA	NA	NA	NA	0.0 (0.0)	0.0 (0.0)
Spain	1	42.3 (17.9)	24.9 (23.3)	26.2 (20.9)	20.8 (19.9)	18.5 (20.9)	32.2 (22.8)	26.7 (16.9)	31.4 (25.1)	22.9 (17.9)
	2	10.7 (4.5)	5.4 (5.1)	4.1 (3.3)	2.7 (2.5)	2.2 (2.5)	8.4 (6.0)	19.0 (12.0)	7.0 (5.6)	11.0 (8.6)
	3	16.0 (6.8)	7.0 (6.6)	5.6 (4.5)	10.8 (10.3)	10.5 (11.9)	17.3 (12.2)	19.2 (12.1)	16.6 (13.2)	18.5 (14.5)
	4	2.4 (1.0)	1.7 (1.6)	2.7 (2.2)	2.1 (2.0)	0.8 (0.9)	1.3 (0.9)	1.3 (0.8)	1.4 (1.2)	1.0 (0.7)
	5	6.8 (2.9)	1.3 (1.2)	1.7 (1.4)	1.3 (1.2)	1.7 (2.0)	2.1 (1.5)	1.9 (1.2)	1.6 (1.3)	2.5 (1.9)
	6	22.7 (9.6)	11.4 (10.6)	15.3 (12.2)	11.1 (10.7)	9.9 (11.2)	14.9 (10.5)	11.6 (7.3)	14.1 (11.3)	13.8 (10.8)
	7	0.7 (0.3)	2.4 (2.2)	1.9 (1.5)	0.5 (0.5)	0.3 (0.4)	1.5 (1.1)	1.9 (1.2)	1.7 (1.3)	1.0 (0.8)
	8	15.6 (6.6)	6.1 (5.7)	8.5 (6.8)	8.9 (8.5)	5.6 (6.3)	5.9 (4.2)	13.3 (8.4)	4.5 (3.6)	6.6 (5.1)
	9	3.1 (1.3)	3.6 (3.4)	0.8 (0.6)	0.5 (0.5)	0.8 (1.0)	1.4 (1.0)	3.3 (2.1)	2.6 (2.1)	1.7 (1.3)

	10	0.8 (0.3)	0.0 (0.0)	0.2 (0.2)	0.2 (0.2)	0.3 (0.3)	1.2 (0.9)	1.1 (0.7)	0.7 (0.6)	0.7 (0.5)
	11	15.1 (6.4)	1.3 (1.3)	2.7 (2.2)	1.6 (1.6)	1.3 (1.5)	2.0 (1.4)	1.6 (1.0)	1.2 (0.9)	1.6 (1.2)
	12	70.2 (29.8)	23.8 (22.3)	32.7 (26.1)	25.3 (24.3)	24.1 (27.2)	28.6 (20.2)	33.3 (21.0)	22.0 (17.6)	26.7 (20.8)
	13	3.3 (1.4)	1.0 (0.9)	3.4 (2.7)	2.3 (2.2)	1.5 (1.7)	1.6 (1.1)	1.1 (0.7)	2.2 (1.8)	1.1 (0.9)
	14	6.6 (2.8)	5.2 (4.9)	10.5 (8.3)	7.3 (6.9)	2.3 (2.6)	11.2 (7.9)	5.6 (3.5)	6.3 (5.1)	6.6 (5.2)
	15	7.7 (3.3)	5.4 (5.1)	5.1 (4.1)	2.5 (2.4)	4.2 (4.8)	5.7 (4.0)	7.8 (4.9)	7.8 (6.2)	7.0 (5.5)
	16	10.4 (4.4)	4.6 (4.3)	2.3 (1.8)	5.0 (4.8)	3.8 (4.3)	5.1 (3.6)	9.0 (5.7)	2.4 (1.9)	1.2 (1.0)
	17	1.4 (0.6)	1.5 (1.4)	1.7 (1.4)	1.5 (1.5)	0.5 (0.6)	1.1 (0.8)	0.9 (0.5)	1.1 (0.9)	0.7 (0.5)
	18	NA	NA	NA	NA	NA	NA	NA	0.1 (0.1)	2.9 (2.3)
	19	NA	NA	NA	NA	NA	NA	NA	0.0 (0.0)	0.0 (0.0)
	20	NA	NA	NA	NA	NA	NA	NA	0.2 (0.1)	0.2 (0.1)
	21	NA	NA	NA	NA	NA	NA	NA	0.1 (0.1)	0.1 (0.1)
	22	NA	NA	NA	NA	NA	NA	NA	0.0 (0.0)	0.4 (0.3)
	23	NA	NA	NA	NA	NA	NA	NA	0.0 (0.0)	0.0 (0.0)
Sweden	1	125.8 (28.7)	66.7 (14.3)	124.9 (26.1)	89.8 (20.8)	77.8 (16.9)	65.9 (14.2)	90.7 (17.8)	93.2 (18.3)	100.4 (21.7)
	2	1.6 (0.4)	1.0 (0.2)	2.4 (0.5)	3.0 (0.7)	1.6 (0.3)	2.8 (0.6)	9.2 (1.8)	4.8 (0.9)	6.2 (1.3)
	3	6.5 (1.5)	6.3 (1.3)	12.3 (2.6)	12.6 (2.9)	12.3 (2.7)	18.1 (3.9)	20.8 (4.1)	14.3 (2.8)	15.5 (3.4)
	4	4.5 (1.0)	4.6 (1.0)	8.1 (1.7)	10.9 (2.5)	3.6 (0.8)	5.6 (1.2)	6.4 (1.2)	13.2 (2.6)	10.7 (2.3)
	5	3.4 (0.8)	2.3 (0.5)	6.1 (1.3)	5.1 (1.2)	10.5 (2.3)	5.7 (1.2)	7.0 (1.4)	5.3 (1.0)	2.6 (0.6)
	6	55.0 (12.5)	94.1 (20.2)	63.4 (13.3)	70.9 (16.4)	80.7 (17.6)	107.7 (23.2)	121.7 (23.9)	112.2 (22.0)	121.9 (26.3)
	7	0.0 (0.0)	4.2 (0.9)	4.4 (0.9)	5.9 (1.4)	5.0 (1.1)	5.2 (1.1)	6.4 (1.3)	9.1 (1.8)	4.0 (0.9)
	8	13.5 (3.1)	4.3 (0.9)	14.1 (2.9)	17.3 (4.0)	36.4 (7.9)	20.4 (4.4)	25.4 (5.0)	28.1 (5.5)	31.4 (6.8)
	9	20.3 (4.6)	35.9 (7.7)	25.6 (5.3)	20.4 (4.7)	30.5 (6.6)	30.9 (6.6)	30.7 (6.0)	31.3 (6.1)	33.1 (7.1)
	10	13.0 (3.0)	17.6 (3.8)	18.3 (3.8)	10.1 (2.3)	15.6 (3.4)	17.6 (3.8)	16.5 (3.2)	15.3 (3.0)	14.7 (3.2)
	11	122.4 (27.9)	145.5 (31.2)	117.9 (24.6)	81.7 (18.9)	93.3 (20.3)	94.8 (20.4)	73.0 (14.3)	75.6 (14.8)	44.8 (9.7)
	12	20.6 (4.7)	23.7 (5.1)	18.9 (3.9)	27.1 (6.3)	24.6 (5.4)	45.1 (9.7)	41.9 (8.2)	45.3 (8.9)	35.1 (7.6)
	13	2.0 (0.5)	0.7 (0.2)	1.0 (0.2)	0.9 (0.2)	0.7 (0.2)	1.0 (0.2)	0.8 (0.1)	1.0 (0.2)	0.3 (0.1)
	14	23.0 (5.2)	18.1 (3.9)	20.8 (4.4)	28.6 (6.6)	28.8 (6.3)	15.9 (3.4)	31.9 (6.3)	34.1 (6.7)	19.6 (4.2)
	15	2.9 (0.7)	4.8 (1.0)	5.0 (1.0)	5.5 (1.3)	9.9 (2.2)	5.4 (1.2)	5.8 (1.1)	6.4 (1.3)	6.8 (1.5)
	16	24.0 (5.5)	36.5 (7.8)	35.6 (7.4)	42.7 (9.9)	21.5 (4.7)	16.6 (3.6)	14.2 (2.8)	18.8 (3.7)	9.8 (2.1)
	17	0.0 (0.0)	0.0 (0.0)	0.0 (0.0)	0.1 (0.0)	6.2 (1.4)	6.0 (1.3)	6.8 (1.3)	2.1 (0.4)	1.4 (0.3)
	18	NA	NA	NA	NA	NA	NA	NA	0.0 (0.0)	3.1 (0.7)
	19	NA	NA	NA	NA	NA	NA	NA	0.0 (0.0)	0.0 (0.0)
	20	NA	NA	NA	NA	NA	NA	NA	0.0 (0.0)	0.0 (0.0)
	21	NA	NA	NA	NA	NA	NA	NA	0.0 (0.0)	1.9 (0.4)
	22	NA	NA	NA	NA	NA	NA	NA	0.0 (0.0)	0.0 (0.0)
	23	NA	NA	NA	NA	NA	NA	NA	0.1 (0.0)	0.0 (0.0)
Switzerland	1	36.2 (25.4)	35.6 (24.6)	43.3 (29.4)	44.8 (21.8)	45.1 (22.7)	49.3 (26.6)	44.7 (20.9)	46.0 (22.7)	45.6 (22.6)
	2	6.1 (4.3)	8.0 (5.5)	1.5 (1.0)	1.6 (0.8)	2.8 (1.4)	1.7 (0.9)	3.2 (1.5)	2.7 (1.3)	5.5 (2.7)
	3	2.1 (1.4)	1.8 (1.3)	2.7 (1.8)	2.8 (1.4)	3.8 (1.9)	4.8 (2.6)	12.4 (5.8)	17.3 (8.5)	12.3 (6.1)
	4	0.6 (0.5)	0.5 (0.4)	0.7 (0.5)	0.4 (0.2)	0.5 (0.3)	0.4 (0.2)	2.9 (1.4)	3.3 (1.6)	5.5 (2.7)
	5	0.4 (0.3)	0.5 (0.4)	0.3 (0.2)	0.6 (0.3)	0.8 (0.4)	0.3 (0.2)	0.5 (0.2)	0.4 (0.2)	0.6 (0.3)
	6	20.8 (14.6)	17.2 (11.9)	14.3 (9.7)	15.2 (7.4)	14.0 (7.0)	14.9 (8.1)	15.6 (7.3)	14.1 (6.9)	14.8 (7.4)
	7	0.0 (0.0)	1.8 (1.2)	0.4 (0.2)	1.6 (0.8)	1.2 (0.6)	1.8 (1.0)	1.7 (0.8)	2.6 (1.3)	1.3 (0.6)
	8	9.4 (6.6)	10.0 (6.9)	13.5 (9.2)	33.2 (16.2)	28.5 (14.4)	11.6 (6.3)	17.8 (8.3)	16.1 (7.9)	18.2 (9.0)
	9	4.1 (2.9)	3.9 (2.7)	4.0 (2.7)	7.2 (3.5)	7.3 (3.7)	10.3 (5.6)	11.3 (5.3)	10.9 (5.4)	12.4 (6.1)
	10	2.1 (1.5)	1.5 (1.0)	2.5 (1.7)	3.4 (1.6)	3.4 (1.7)	4.3 (2.3)	4.3 (2.0)	3.8 (1.9)	3.8 (1.9)

	11	20.8 (14.6)	19.4 (13.4)	20.0 (13.5)	20.5 (10.0)	27.2 (13.7)	28.0 (15.1)	25.4 (11.9)	22.8 (11.2)	14.6 (7.3)
	12	30.8 (21.6)	25.5 (17.7)	36.7 (24.9)	49.8 (24.3)	46.6 (23.5)	40.6 (21.9)	51.5 (24.0)	46.5 (22.9)	45.8 (22.7)
	13	0.5 (0.4)	0.6 (0.4)	2.7 (1.8)	1.5 (0.7)	3.2 (1.6)	4.1 (2.2)	1.8 (0.8)	0.8 (0.4)	0.5 (0.2)
	14	1.3 (0.9)	0.4 (0.3)	0.0 (0.0)	8.4 (4.1)	0.5 (0.3)	0.4 (0.2)	0.5 (0.3)	0.3 (0.2)	0.8 (0.4)
	15	1.6 (1.1)	1.8 (1.2)	2.1 (1.4)	3.3 (1.6)	5.0 (2.5)	6.8 (3.7)	15.1 (7.0)	9.5 (4.7)	13.0 (6.5)
	16	5.8 (4.0)	16.1 (11.1)	2.9 (1.9)	11.1 (5.4)	6.9 (3.5)	4.0 (2.1)	3.3 (1.6)	3.9 (1.9)	2.3 (1.2)
	17	0.0 (0.0)	0.0 (0.0)	0.0 (0.0)	0.1 (0.0)	1.8 (0.9)	1.8 (1.0)	2.2 (1.0)	0.8 (0.4)	0.3 (0.2)
	18	NA	NA	NA	NA	NA	NA	NA	0.1 (0.0)	0.8 (0.4)
	19	NA	NA	NA	NA	NA	NA	NA	0.0 (0.0)	0.0 (0.0)
	20	NA	NA	NA	NA	NA	NA	NA	0.1 (0.0)	0.1 (0.1)
	21	NA	NA	NA	NA	NA	NA	NA	1.0 (0.5)	2.8 (1.4)
	22	NA	NA	NA	NA	NA	NA	NA	0.1 (0.0)	0.3 (0.1)
	23	NA	NA	NA	NA	NA	NA	NA	0.0 (0.0)	0.0 (0.0)
United Kingdom	1	332.4 (16.2)	511.6 (23.4)	760.5 (23.2)	707.0 (25.1)	567.9 (25.5)	484.8 (22.1)	485.6 (17.3)	454.2 (15.8)	412.4 (14.4)
	2	10.4 (0.5)	3.7 (0.2)	10.4 (0.3)	8.6 (0.3)	8.0 (0.4)	13.6 (0.6)	35.6 (1.3)	14.8 (0.5)	18.0 (0.6)
	3	55.3 (2.7)	68.8 (3.2)	112.0 (3.4)	108.7 (3.9)	122.6 (5.5)	193.7 (8.8)	242.4 (8.6)	206.1 (7.2)	196.5 (6.9)
	4	5.8 (0.3)	26.9 (1.2)	42.3 (1.3)	40.5 (1.4)	29.2 (1.3)	24.3 (1.1)	30.9 (1.1)	24.2 (0.8)	29.7 (1.0)
	5	5.1 (0.2)	6.1 (0.3)	4.8 (0.1)	11.6 (0.4)	9.4 (0.4)	6.3 (0.3)	21.1 (0.8)	18.3 (0.6)	18.0 (0.6)
	6	303.2 (14.7)	294.4 (13.5)	330.7 (10.1)	386.4 (13.7)	342.4 (15.4)	333.2 (15.2)	264.4 (9.4)	240.4 (8.4)	209.7 (7.3)
	7	22.0 (1.1)	159.0 (7.3)	53.9 (1.6)	229.9 (8.2)	148.1 (6.7)	121.0 (5.5)	195.0 (6.9)	239.1 (8.3)	325.3 (11.4)
	8	276.5 (13.4)	194.8 (8.9)	393.1 (12.0)	221.1 (7.8)	290.3 (13.1)	214.4 (9.8)	275.7 (9.8)	297.1 (10.3)	377.5 (13.2)
	9	187.0 (9.1)	196.8 (9.0)	461.0 (14.1)	343.3 (12.2)	166.6 (7.5)	200.7 (9.1)	219.2 (7.8)	211.5 (7.4)	197.3 (6.9)
	10	69.3 (3.4)	39.2 (1.8)	170.8 (5.2)	92.5 (3.3)	42.3 (1.9)	47.0 (2.1)	82.8 (3.0)	83.7 (2.9)	77.5 (2.7)
	11	469.6 (22.8)	298.3 (13.7)	516.4 (15.8)	290.6 (10.3)	122.2 (5.5)	135.0 (6.2)	192.3 (6.9)	194.0 (6.7)	164.3 (5.7)
	12	168.7 (8.2)	208.8 (9.6)	260.9 (8.0)	228.3 (8.1)	201.1 (9.0)	215.4 (9.8)	295.1 (10.5)	319.3 (11.1)	274.5 (9.6)
	13	3.0 (0.1)	3.1 (0.1)	2.3 (0.1)	2.8 (0.1)	1.9 (0.1)	3.2 (0.1)	1.7 (0.1)	4.4 (0.2)	1.5 (0.1)
	14	100.4 (4.9)	121.3 (5.6)	110.6 (3.4)	93.8 (3.3)	120.6 (5.4)	157.3 (7.2)	382.5 (13.6)	493.6 (17.2)	478.7 (16.7)
	15	11.4 (0.6)	8.3 (0.4)	6.5 (0.2)	7.1 (0.3)	12.6 (0.6)	12.9 (0.6)	18.8 (0.7)	31.5 (1.1)	15.3 (0.5)
	16	36.1 (1.8)	40.6 (1.9)	38.3 (1.2)	33.5 (1.2)	25.9 (1.2)	17.4 (0.8)	28.6 (1.0)	15.2 (0.5)	15.8 (0.6)
	17	0.1 (0.0)	1.3 (0.1)	1.5 (0.0)	14.4 (0.5)	13.0 (0.6)	14.4 (0.7)	35.4 (1.3)	26.8 (0.9)	19.3 (0.7)
	18	NA	NA	NA	NA	NA	NA	NA	0.1 (0.0)	9.0 (0.3)
	19	NA	NA	NA	NA	NA	NA	NA	0.0 (0.0)	1.2 (0.0)
	20	NA	NA	NA	NA	NA	NA	NA	0.0 (0.0)	0.0 (0.0)
	21	NA	NA	NA	NA	NA	NA	NA	0.0 (0.0)	1.0 (0.0)
	22	NA	NA	NA	NA	NA	NA	NA	0.0 (0.0)	0.8 (0.0)
	23	NA	NA	NA	NA	NA	NA	NA	0.0 (0.0)	19.3 (0.7)
United States	1	290.7 (2.9)	323.5 (3.3)	349.7 (3.4)	341.9 (3.3)	406.0 (4.4)	527.0 (4.7)	654.5 (5.9)	583.7 (5.7)	612.7 (7.6)
	2	39.0 (0.4)	28.1 (0.3)	87.5 (0.9)	6.2 (0.1)	14.7 (0.2)	25.5 (0.2)	16.2 (0.1)	8.4 (0.1)	4.5 (0.1)
	3	128.0 (1.3)	272.0 (2.8)	310.4 (3.0)	262.5 (2.5)	314.5 (3.4)	172.1 (1.5)	187.8 (1.7)	160.5 (1.6)	161.7 (2.0)
	4	8.4 (0.1)	8.6 (0.1)	8.7 (0.1)	2.6 (0.0)	4.9 (0.1)	6.3 (0.1)	12.9 (0.1)	12.4 (0.1)	9.2 (0.1)
	5	12.4 (0.1)	8.6 (0.1)	7.0 (0.1)	23.4 (0.2)	21.5 (0.2)	3.2 (0.0)	6.4 (0.1)	4.3 (0.0)	7.1 (0.1)
	6	527.1 (5.3)	587.8 (5.9)	566.6 (5.5)	595.8 (5.7)	720.2 (7.9)	665.0 (6.0)	724.9 (6.6)	667.7 (6.5)	576.8 (7.2)
	7	591.0 (6.0)	545.4 (5.5)	648.3 (6.3)	568.3 (5.4)	681.1 (7.4)	482.9 (4.3)	496.8 (4.5)	624.9 (6.1)	419.7 (5.2)
	8	332.7 (3.4)	282.8 (2.9)	306.1 (3.0)	352.8 (3.4)	735.4 (8.0)	897.1 (8.1)	920.0 (8.3)	461.1 (4.5)	447.0 (5.6)
	9	810.1 (8.2)	970.4 (9.8)	904.7 (8.8)	1258.4 (12.0)	872.5 (9.5)	1243.2 (11.2)	901.4 (8.2)	886.1 (8.6)	1222.9 (15.2)
	10	398.8 (4.0)	413.4 (4.2)	498.2 (4.9)	540.0 (5.2)	303.6 (3.3)	519.2 (4.7)	449.6 (4.1)	421.6 (4.1)	356.5 (4.4)
	11	6365.5 (64.3)	6196.9 (62.7)	6293.0 (61.5)	6151.2 (58.8)	4837.4 (52.8)	6011.3 (54.1)	6493.4 (58.8)	5893.4 (57.4)	3945.2 (49.0)

	12	326.2 (3.3)	185.6 (1.9)	182.4 (1.8)	289.9 (2.8)	179.6 (2.0)	488.2 (4.4)	109.2 (1.0)	471.2 (4.6)	213.7 (2.7)
	13	6.0 (0.1)	6.3 (0.1)	5.1 (0.1)	4.8 (0.0)	3.2 (0.0)	3.0 (0.0)	3.7 (0.0)	4.1 (0.0)	1.6 (0.0)
	14	0.8 (0.0)	0.3 (0.0)	0.5 (0.0)	6.5 (0.1)	6.0 (0.1)	2.9 (0.0)	5.2 (0.0)	4.5 (0.0)	8.8 (0.1)
	15	19.5 (0.2)	16.4 (0.2)	14.2 (0.1)	13.3 (0.1)	16.8 (0.2)	11.7 (0.1)	17.0 (0.2)	16.4 (0.2)	10.8 (0.1)
	16	50.7 (0.5)	42.8 (0.4)	42.6 (0.4)	45.3 (0.4)	45.7 (0.5)	44.0 (0.4)	35.6 (0.3)	39.5 (0.4)	41.6 (0.5)
	17	0.0 (0.0)	1.0 (0.0)	0.2 (0.0)	0.4 (0.0)	4.1 (0.0)	4.2 (0.0)	0.6 (0.0)	1.7 (0.0)	0.3 (0.0)
	18	NA	NA	NA	NA	NA	NA	NA	0.0 (0.0)	8.2 (0.1)
	19	NA	NA	NA	NA	NA	NA	NA	0.0 (0.0)	0.0 (0.0)
	20	NA	NA	NA	NA	NA	NA	NA	0.0 (0.0)	0.0 (0.0)
	21	NA	NA	NA	NA	NA	NA	NA	0.0 (0.0)	0.0 (0.0)
	22	NA	NA	NA	NA	NA	NA	NA	0.0 (0.0)	1.2 (0.0)
	23	NA	NA	NA	NA	NA	NA	NA	0.0 (0.0)	0.0 (0.0)
All members	1	1771.6 (9.5)	1801.1 (9.6)	2224.4 (10.9)	2031.7 (10.0)	1839.3 (10.1)	2142.7 (10.5)	2246.2 (10.5)	2157.0 (10.4)	2182.7 (11.8)
	2	287.3 (1.5)	301.3 (1.6)	351.8 (1.7)	265.9 (1.3)	341.5 (1.9)	453.1 (2.2)	565.7 (2.6)	377.6 (1.8)	467.7 (2.5)
	3	411.7 (2.2)	662.5 (3.5)	848.0 (4.2)	817.9 (4.0)	804.2 (4.4)	785.9 (3.8)	833.9 (3.9)	856.7 (4.1)	810.0 (4.4)
	4	65.6 (0.4)	98.5 (0.5)	138.5 (0.7)	105.5 (0.5)	91.9 (0.5)	95.5 (0.5)	127.9 (0.6)	124.3 (0.6)	121.5 (0.7)
	5	84.8 (0.5)	87.0 (0.5)	92.3 (0.5)	108.0 (0.5)	134.5 (0.7)	112.8 (0.6)	119.7 (0.6)	96.2 (0.5)	108.4 (0.6)
	6	1432.0 (7.7)	1468.2 (7.9)	1572.7 (7.7)	1667.0 (8.2)	1759.9 (9.7)	1712.3 (8.4)	1721.7 (8.0)	1646.0 (7.9)	1512.0 (8.2)
	7	646.5 (3.5)	772.0 (4.1)	794.1 (3.9)	892.6 (4.4)	893.8 (4.9)	704.8 (3.5)	838.6 (3.9)	1050.2 (5.1)	914.9 (4.9)
	8	1084.8 (5.8)	867.7 (4.6)	1141.4 (5.6)	1100.8 (5.4)	1911.7 (10.5)	1909.6 (9.3)	2090.6 (9.7)	1718.2 (8.3)	1785.6 (9.6)
	9	1359.1 (7.3)	1677.5 (9.0)	1792.8 (8.8)	2065.9 (10.2)	1530.4 (8.4)	1890.0 (9.3)	1662.5 (7.8)	1647.8 (7.9)	2031.8 (11.0)
	10	769.2 (4.1)	729.4 (3.9)	971.1 (4.8)	887.6 (4.4)	621.0 (3.4)	857.4 (4.2)	848.6 (4.0)	824.1 (4.0)	748.6 (4.0)
	11	8236.1 (44.3)	7876.5 (42.2)	7946.2 (39.1)	7566.4 (37.2)	6023.1 (33.1)	7041.7 (34.5)	7477.5 (34.9)	6873.6 (33.1)	4776.3 (25.8)
	12	1536.3 (8.3)	1331.7 (7.1)	1359.5 (6.7)	1606.2 (7.9)	1176.4 (6.5)	1584.0 (7.8)	1428.3 (6.7)	1726.8 (8.3)	1436.6 (7.8)
	13	87.4 (0.5)	78.9 (0.4)	83.0 (0.4)	97.1 (0.5)	76.4 (0.4)	111.4 (0.5)	126.8 (0.6)	106.3 (0.5)	80.2 (0.4)
	14	223.9 (1.2)	239.0 (1.3)	279.5 (1.4)	262.1 (1.3)	261.3 (1.4)	282.5 (1.4)	500.1 (2.3)	654.5 (3.2)	634.3 (3.4)
	15	286.1 (1.5)	241.7 (1.3)	283.1 (1.4)	325.9 (1.6)	338.5 (1.9)	379.2 (1.9)	398.0 (1.9)	464.6 (2.2)	488.7 (2.6)
	16	298.8 (1.6)	410.9 (2.2)	376.1 (1.9)	457.5 (2.3)	276.2 (1.5)	239.2 (1.2)	304.1 (1.4)	308.4 (1.5)	232.9 (1.3)
	17	26.3 (0.1)	40.6 (0.2)	61.1 (0.3)	73.7 (0.4)	119.8 (0.7)	123.2 (0.6)	157.6 (0.7)	123.4 (0.6)	87.5 (0.5)
	18	NA	NA	NA	NA	NA	NA	NA	2.8 (0.0)	63.0 (0.3)
	19	NA	NA	NA	NA	NA	NA	NA	0.8 (0.0)	1.5 (0.0)
	20	NA	NA	NA	NA	NA	NA	NA	2.3 (0.0)	0.8 (0.0)
	21	NA	NA	NA	NA	NA	NA	NA	2.1 (0.0)	9.0 (0.0)
	22	NA	NA	NA	NA	NA	NA	NA	3.3 (0.0)	14.7 (0.1)
	23	NA	NA	NA	NA	NA	NA	NA	2.3 (0.0)	26.5 (0.1)

DAH: development assistance for health; DAH: Development Assistance Committee; 1: Basic health care; 2: Basic health infrastructure; 3: Basic nutrition; 4: Health education; 5: Health personnel development; 6: Reproductive health care; 7: Family planning; 8: Infectious disease control; 9: Malaria control; 10: Tuberculosis control; 11: STD control incl. HIV/AIDS; 12: Health policy & admin. management; 13: Medical education/training; 14: Medical research; 15: Medical services; 16: Population policy & admin. management; 17: Personnel development for population & reproductive health; 18: NCDs control, general; 19: Tobacco use control; 20: Control of harmful use of alcohol and drugs; 21: Promotion of mental health and well-being; 22: Other prevention and treatment of NCDs; 23: Research for prevention and control of NCDs.

Table S7: Estimated DAH in constant prices at 2019 from all the 29 DAC member countries for PHC delivery and HSS in support of PHC delivery (%), 2011–2019

Country	PHC and HSS	2011	2012	2013	2014	2015	2016	2017	2018	2019
Australia	PHC definition #1	111.9 (23.2)	178.4 (31.9)	119.9 (26.0)	154.2 (35.0)	104.2 (28.4)	83.7 (24.2)	87.6 (35.2)	82.9 (25.5)	143.6 (46.8)
	PHC definition #2	74.7 (15.5)	54.8 (9.8)	61.5 (13.3)	38.9 (8.8)	40.0 (10.8)	22.5 (6.5)	25.8 (10.3)	24.8 (7.7)	19.4 (6.3)
	PHC definition #3	38.4 (7.9)	52.1 (9.3)	57.6 (12.5)	68.7 (15.5)	70.4 (19.2)	104.3 (30.3)	39.5 (15.8)	104.4 (32.1)	73.1 (23.9)
	PHC definition #4	92.3 (19.2)	115.8 (20.7)	114.3 (24.8)	101.5 (23.0)	82.7 (22.5)	75.8 (22.0)	17.8 (7.1)	33.8 (10.4)	7.9 (2.6)
	Broader PHC definition	317.3 (65.8)	401.1 (71.7)	353.3 (76.6)	363.3 (82.3)	297.3 (80.9)	286.3 (83.0)	170.7 (68.4)	245.9 (75.7)	244.0 (79.6)
	HSS definition #1	133.6 (27.8)	118.1 (21.1)	78.9 (17.1)	60.5 (13.7)	48.5 (13.2)	38.8 (11.2)	50.6 (20.3)	69.3 (21.3)	45.8 (15.0)
	HSS definition #2	25.2 (5.3)	20.3 (3.6)	19.9 (4.3)	8.1 (1.8)	15.1 (4.1)	14.4 (4.1)	9.8 (4.0)	7.5 (2.3)	11.7 (3.8)
	HSS definition #3	4.3 (0.9)	19.2 (3.4)	9.6 (2.1)	8.7 (2.0)	6.8 (1.9)	5.6 (1.6)	18.0 (7.2)	2.4 (0.8)	4.7 (1.5)
	Broader HSS definition	163.1 (34.0)	157.6 (28.1)	108.4 (23.5)	77.3 (17.5)	70.4 (19.2)	58.8 (16.9)	78.4 (31.5)	79.2 (24.4)	62.2 (20.3)
Austria	PHC definition #1	13.9 (29.7)	12.6 (32.3)	14.3 (37.0)	15.5 (36.9)	10.7 (27.8)	18.9 (42.4)	22.4 (46.9)	15.5 (40.8)	17.7 (38.9)
	PHC definition #2	2.7 (5.8)	2.3 (5.8)	2.7 (6.8)	1.7 (4.2)	2.0 (5.1)	3.6 (8.1)	2.8 (5.7)	2.5 (6.7)	2.4 (5.4)
	PHC definition #3	5.7 (12.1)	4.4 (11.1)	5.2 (13.5)	8.6 (20.2)	9.7 (25.0)	3.5 (7.6)	5.2 (10.9)	3.2 (8.6)	4.9 (10.8)
	PHC definition #4	3.7 (7.9)	2.6 (6.6)	2.0 (5.3)	1.3 (3.0)	1.5 (3.8)	0.9 (2.1)	0.7 (1.5)	0.5 (1.3)	0.8 (1.8)
	Broader PHC definition	26.0 (55.5)	21.9 (55.8)	24.2 (62.6)	27.1 (64.3)	23.9 (61.7)	26.9 (60.2)	31.1 (65.0)	21.7 (57.4)	25.8 (56.9)
	HSS definition #1	10.7 (23.0)	10.0 (25.5)	8.9 (23.1)	7.6 (18.1)	6.3 (16.4)	7.0 (15.6)	9.1 (19.0)	7.9 (20.7)	9.0 (19.7)
	HSS definition #2	8.1 (17.3)	6.3 (16.2)	4.5 (11.8)	6.2 (14.6)	6.6 (17.2)	9.2 (20.6)	4.8 (9.9)	7.4 (19.7)	10.1 (22.3)
	HSS definition #3	1.9 (4.1)	1.0 (2.5)	0.9 (2.4)	1.2 (3.0)	1.9 (4.7)	1.6 (3.7)	2.9 (6.1)	0.8 (2.1)	0.6 (1.2)
	Broader HSS definition	20.7 (44.4)	17.3 (44.2)	14.3 (37.3)	15.0 (35.7)	14.8 (38.3)	17.8 (39.9)	16.8 (35.0)	16.1 (42.5)	19.7 (43.2)
Belgium	PHC definition #1	65.6 (26.8)	61.2 (26.8)	59.0 (26.2)	64.7 (26.8)	59.5 (28.3)	58.8 (28.8)	62.8 (29.7)	57.2 (30.7)	63.0 (34.2)
	PHC definition #2	12.0 (4.9)	8.5 (3.8)	9.6 (4.3)	10.7 (4.4)	6.9 (3.3)	12.1 (5.9)	18.5 (8.7)	13.8 (7.4)	16.0 (8.6)
	PHC definition #3	39.3 (16.0)	38.8 (17.1)	32.9 (14.6)	33.5 (13.9)	34.5 (16.4)	33.3 (16.3)	28.8 (13.5)	33.8 (18.2)	38.1 (20.6)
	PHC definition #4	31.5 (12.8)	23.8 (10.5)	20.7 (9.2)	19.1 (7.9)	18.0 (8.5)	16.4 (8.0)	11.8 (5.6)	11.7 (6.3)	7.4 (4.0)
	Broader PHC definition	148.4 (60.5)	132.3 (58.2)	122.2 (54.3)	128.0 (53.0)	118.9 (56.5)	120.6 (59.0)	121.9 (57.5)	116.5 (62.6)	124.5 (67.4)
	HSS definition #1	53.6 (21.8)	51.3 (22.5)	64.9 (28.9)	65.8 (27.3)	44.5 (21.2)	36.6 (18.0)	46.0 (21.8)	34.7 (18.8)	25.8 (14.0)
	HSS definition #2	38.0 (15.4)	36.0 (15.8)	30.9 (13.8)	38.5 (16.0)	40.2 (19.1)	40.6 (20.0)	34.9 (16.5)	27.8 (15.1)	31.5 (17.2)
	HSS definition #3	5.7 (2.3)	8.1 (3.5)	7.1 (3.1)	8.8 (3.6)	6.4 (3.1)	6.0 (2.9)	8.8 (4.2)	6.1 (3.3)	2.7 (1.5)
	Broader HSS definition	97.3 (39.5)	95.4 (41.8)	102.9 (45.8)	113.1 (46.9)	91.1 (43.4)	83.2 (40.9)	89.7 (42.5)	68.6 (37.2)	60.0 (32.7)
Canada	PHC definition #1	293.2 (35.9)	311.2 (38.7)	371.8 (42.8)	274.4 (45.2)	259.6 (30.0)	340.8 (43.4)	341.7 (38.9)	302.3 (33.6)	267.2 (31.6)
	PHC definition #2	29.7 (3.7)	34.5 (4.3)	39.4 (4.5)	36.6 (6.0)	35.7 (4.1)	37.1 (4.7)	78.8 (9.0)	114.0 (12.7)	117.4 (13.9)
	PHC definition #3	219.8 (27.0)	186.9 (23.2)	198.7 (22.9)	96.3 (15.9)	268.4 (31.0)	184.3 (23.5)	214.8 (24.5)	217.7 (24.3)	204.3 (24.3)
	PHC definition #4	139.5 (17.2)	137.6 (17.1)	105.7 (12.2)	23.7 (3.9)	171.1 (19.8)	86.3 (11.0)	84.7 (9.6)	79.6 (8.9)	68.4 (8.1)
	Broader PHC definition	682.2 (83.8)	670.2 (83.3)	715.6 (82.4)	431.0 (71.0)	734.8 (84.9)	648.5 (82.6)	720.0 (82.0)	713.6 (79.5)	657.3 (77.9)
	HSS definition #1	79.4 (9.8)	69.3 (8.6)	61.1 (7.0)	56.4 (9.3)	42.8 (4.9)	50.6 (6.4)	61.9 (7.0)	60.3 (6.7)	47.6 (5.6)
	HSS definition #2	26.7 (3.2)	47.6 (5.8)	57.4 (6.6)	54.0 (8.9)	49.0 (5.7)	52.2 (6.6)	28.7 (3.2)	30.7 (3.3)	31.3 (3.6)
	HSS definition #3	24.7 (3.0)	18.8 (2.4)	33.8 (3.9)	67.1 (11.0)	38.5 (4.4)	34.5 (4.4)	68.4 (7.8)	92.7 (10.3)	108.1 (12.8)
	Broader HSS definition	130.8 (16.0)	135.7 (16.8)	152.3 (17.5)	177.5 (29.2)	130.3 (15.0)	137.3 (17.4)	159.0 (18.0)	183.7 (20.3)	187.0 (22.0)
Czech Republic	PHC definition #1	3.8 (37.6)	3.2 (39.1)	3.9 (40.6)	4.2 (50.7)	4.3 (44.1)	6.2 (48.9)	9.9 (58.7)	4.2 (50.5)	7.5 (58.2)
	PHC definition #2	0.6 (5.8)	0.4 (5.3)	0.6 (6.5)	0.6 (6.9)	0.7 (7.1)	0.7 (5.9)	0.8 (4.7)	0.5 (6.1)	0.3 (2.7)
	PHC definition #3	0.8 (8.8)	0.7 (8.6)	1.1 (10.7)	0.4 (5.2)	0.7 (8.0)	0.7 (4.7)	0.6 (3.4)	0.5 (5.7)	0.5 (4.1)
	PHC definition #4	0.5 (4.7)	0.6 (6.7)	0.2 (2.0)	0.1 (1.0)	0.2 (1.9)	0.1 (1.1)	0.1 (0.6)	0.0 (0.5)	0.1 (0.6)
	Broader PHC definition	5.7 (56.9)	4.9 (59.7)	5.8 (59.8)	5.3 (63.8)	5.9 (61.1)	7.7 (60.6)	11.4 (67.4)	5.2 (62.8)	8.4 (65.6)
	HSS definition #1	2.0 (19.7)	1.6 (19.7)	1.9 (19.6)	0.9 (10.1)	2.0 (20.6)	2.2 (17.2)	2.7 (15.9)	1.8 (22.0)	2.5 (19.7)
	HSS definition #2	1.7 (16.7)	1.3 (15.3)	1.6 (17.0)	1.8 (20.7)	1.3 (13.3)	2.1 (16.4)	1.5 (9.1)	1.1 (13.6)	1.8 (14.0)
	HSS definition #3	0.7 (6.6)	0.4 (5.3)	0.4 (3.8)	0.4 (5.3)	0.5 (5.1)	0.7 (5.8)	1.2 (7.6)	0.1 (1.6)	0.1 (0.8)

	Broader HSS definition	4.4 (43.0)	3.3 (40.3)	3.9 (40.4)	3.1 (36.1)	3.8 (39.0)	5.0 (39.4)	5.4 (32.6)	3.0 (37.2)	4.4 (34.5)
Denmark	PHC definition #1	60.8 (25.7)	60.2 (28.6)	50.7 (27.0)	52.7 (23.8)	29.5 (18.2)	32.6 (22.3)	32.6 (18.4)	21.5 (10.6)	17.8 (10.5)
	PHC definition #2	41.4 (17.5)	26.3 (12.6)	38.1 (20.4)	47.3 (21.5)	39.0 (24.1)	24.5 (16.8)	61.0 (34.6)	50.7 (25.3)	60.6 (35.6)
	PHC definition #3	18.5 (7.8)	14.5 (6.9)	15.3 (8.2)	21.6 (9.9)	15.8 (9.7)	7.7 (5.3)	3.6 (2.0)	20.7 (10.3)	18.8 (11.0)
	PHC definition #4	56.3 (23.8)	37.7 (17.9)	27.6 (14.7)	24.1 (11.0)	18.8 (11.6)	8.9 (6.1)	6.7 (3.8)	15.6 (7.8)	10.0 (5.9)
	Broader PHC definition	177.0 (74.8)	138.7 (66.0)	131.7 (70.3)	145.7 (66.2)	103.1 (63.6)	73.7 (50.5)	103.9 (58.8)	108.5 (54.0)	107.2 (63.0)
	HSS definition #1	41.3 (17.5)	40.6 (19.3)	27.6 (14.8)	33.7 (15.3)	44.9 (27.8)	57.0 (39.1)	41.9 (23.8)	35.8 (17.9)	40.5 (23.9)
	HSS definition #2	2.9 (1.2)	2.5 (1.2)	3.9 (2.1)	2.4 (1.2)	2.9 (1.8)	2.1 (1.5)	2.1 (1.2)	2.4 (1.1)	9.0 (5.3)
	HSS definition #3	15.2 (6.4)	28.4 (13.5)	24.2 (12.9)	38.5 (17.4)	10.9 (6.8)	12.8 (8.8)	28.5 (16.2)	53.4 (26.8)	13.2 (7.8)
	Broader HSS definition	59.4 (25.1)	71.5 (34.0)	55.7 (29.8)	74.6 (33.9)	58.7 (36.4)	71.9 (49.4)	72.5 (41.2)	91.6 (45.8)	62.7 (37.0)
Finland	PHC definition #1	19.8 (18.6)	18.4 (17.3)	20.7 (19.5)	32.9 (23.3)	20.8 (22.5)	18.4 (34.9)	19.5 (34.6)	16.3 (32.8)	18.8 (39.8)
	PHC definition #2	27.7 (26.1)	19.8 (18.7)	22.9 (21.7)	23.1 (16.4)	11.3 (12.2)	9.1 (17.2)	14.2 (25.4)	11.6 (23.5)	10.9 (23.2)
	PHC definition #3	6.0 (5.5)	5.5 (5.3)	6.5 (6.1)	15.9 (11.3)	9.4 (10.2)	2.1 (4.1)	2.7 (4.8)	2.7 (5.6)	2.4 (5.0)
	PHC definition #4	16.9 (15.9)	17.1 (16.1)	13.6 (12.9)	6.5 (4.6)	11.0 (11.8)	2.8 (5.4)	0.7 (1.3)	1.2 (2.4)	0.6 (1.3)
	Broader PHC definition	70.4 (66.1)	60.8 (57.4)	63.7 (60.2)	78.4 (55.6)	52.5 (56.7)	32.4 (61.6)	37.1 (66.1)	31.8 (64.3)	32.7 (69.3)
	HSS definition #1	14.9 (14.1)	16.5 (15.5)	8.6 (8.1)	10.7 (7.6)	9.4 (10.2)	6.1 (11.6)	8.5 (15.2)	6.4 (12.8)	6.2 (13.1)
	HSS definition #2	4.2 (3.9)	3.6 (3.3)	4.1 (3.7)	10.0 (7.0)	9.2 (10.0)	4.9 (9.4)	3.4 (6.0)	5.9 (11.9)	4.0 (8.4)
	HSS definition #3	17.0 (16.0)	25.2 (23.7)	29.5 (28.0)	42.1 (29.8)	21.6 (23.3)	9.1 (17.3)	7.2 (12.8)	5.5 (11.1)	4.3 (9.3)
	Broader HSS definition	36.1 (34.0)	45.3 (42.5)	42.2 (39.8)	62.8 (44.4)	40.2 (43.5)	20.1 (38.3)	19.1 (34.0)	17.8 (35.8)	14.5 (30.8)
France	PHC definition #1	144.8 (19.1)	100.5 (13.3)	133.3 (15.9)	85.6 (7.9)	100.6 (13.5)	141.4 (16.5)	139.3 (16.9)	203.1 (23.2)	187.0 (20.5)
	PHC definition #2	29.6 (3.9)	38.2 (5.1)	43.7 (5.2)	51.7 (4.8)	44.3 (6.0)	29.1 (3.5)	37.9 (4.6)	42.8 (4.9)	32.8 (3.5)
	PHC definition #3	197.0 (25.9)	230.2 (30.5)	231.0 (27.4)	243.9 (22.7)	290.4 (39.0)	355.0 (41.8)	312.5 (38.0)	300.9 (34.3)	331.6 (36.3)
	PHC definition #4	244.7 (32.2)	260.6 (34.5)	223.6 (26.5)	256.6 (23.9)	183.5 (24.6)	161.0 (18.9)	173.2 (21.0)	148.7 (17.0)	149.5 (16.4)
	Broader PHC definition	616.1 (81.1)	629.5 (83.4)	631.6 (75.0)	637.8 (59.3)	618.8 (83.1)	686.5 (80.7)	662.9 (80.5)	695.5 (79.4)	700.9 (76.7)
	HSS definition #1	71.8 (9.4)	60.5 (8.0)	129.2 (15.3)	325.7 (30.3)	49.5 (6.6)	66.0 (7.8)	81.6 (9.9)	69.7 (8.0)	144.2 (15.8)
	HSS definition #2	47.7 (6.2)	29.6 (3.9)	57.0 (6.8)	71.1 (6.6)	34.8 (4.7)	59.3 (7.0)	48.2 (5.8)	97.5 (11.0)	55.5 (6.3)
	HSS definition #3	24.7 (3.2)	35.8 (4.8)	25.3 (3.0)	39.7 (3.7)	41.8 (5.6)	37.7 (4.4)	31.5 (3.8)	12.8 (1.4)	12.5 (1.4)
	Broader HSS definition	144.2 (18.8)	125.9 (16.7)	211.5 (25.1)	436.5 (40.6)	126.1 (16.9)	163.0 (19.2)	161.3 (19.5)	180.0 (20.4)	212.2 (23.5)
Germany	PHC definition #1	175.5 (19.1)	163.0 (18.4)	237.6 (25.2)	248.6 (25.1)	284.3 (28.3)	379.4 (32.3)	388.8 (29.6)	326.0 (23.8)	403.6 (28.5)
	PHC definition #2	64.7 (7.1)	93.1 (10.5)	103.1 (11.0)	118.1 (12.0)	125.8 (12.6)	155.5 (13.2)	128.6 (9.7)	119.7 (8.7)	117.4 (8.3)
	PHC definition #3	186.4 (20.4)	192.4 (21.8)	228.2 (24.3)	245.0 (24.8)	273.6 (27.2)	294.5 (25.1)	405.3 (30.7)	467.0 (34.2)	427.7 (30.2)
	PHC definition #4	286.6 (31.3)	238.1 (27.0)	196.0 (20.8)	208.8 (21.1)	149.6 (14.9)	148.9 (12.7)	136.2 (10.3)	125.4 (9.2)	123.8 (8.7)
	Broader PHC definition	713.2 (77.9)	686.6 (77.7)	764.9 (81.3)	820.5 (83.0)	833.3 (83.0)	978.3 (83.3)	1058.9 (80.3)	1038.1 (75.9)	1072.5 (75.7)
	HSS definition #1	146.3 (15.9)	130.5 (14.8)	120.5 (12.8)	105.4 (10.7)	115.5 (11.5)	122.6 (10.4)	155.6 (11.8)	194.6 (14.3)	173.3 (12.2)
	HSS definition #2	36.2 (4.0)	43.9 (5.0)	35.4 (3.7)	34.7 (3.5)	33.0 (3.3)	44.4 (3.7)	72.7 (5.5)	109.0 (7.9)	158.6 (11.2)
	HSS definition #3	21.6 (2.3)	21.9 (2.5)	22.0 (2.4)	27.6 (2.7)	21.2 (2.1)	30.0 (2.6)	34.1 (2.6)	23.1 (1.7)	10.9 (0.7)
	Broader HSS definition	204.1 (22.2)	196.3 (22.3)	177.9 (18.9)	167.7 (16.9)	169.7 (16.9)	197.0 (16.7)	262.4 (19.9)	326.7 (23.9)	342.8 (24.1)
Greece	PHC definition #1	4.1 (37.3)	2.8 (34.8)	3.8 (41.4)	3.1 (42.9)	3.4 (40.6)	5.0 (50.4)	6.8 (52.8)	4.3 (52.4)	4.8 (58.7)
	PHC definition #2	0.3 (2.7)	0.5 (6.1)	0.5 (5.7)	0.6 (7.4)	0.6 (7.4)	0.5 (5.1)	0.6 (4.5)	0.4 (4.0)	0.1 (2.0)
	PHC definition #3	0.8 (7.3)	0.4 (5.6)	1.1 (12.6)	0.5 (7.5)	0.6 (8.6)	0.5 (6.3)	0.8 (6.0)	0.6 (7.3)	0.3 (3.4)
	PHC definition #4	0.3 (2.7)	0.2 (2.0)	0.1 (1.3)	0.1 (1.2)	0.1 (1.4)	0.1 (0.9)	0.1 (0.8)	0.1 (0.8)	0.0 (0.2)
	Broader PHC definition	5.5 (50.0)	3.9 (48.5)	5.5 (61.0)	4.3 (59.0)	4.7 (58.0)	6.1 (62.7)	8.3 (64.1)	5.4 (64.5)	5.2 (64.3)
	HSS definition #1	3.4 (30.5)	3.0 (38.5)	2.3 (25.2)	1.7 (22.5)	2.3 (27.8)	2.3 (22.7)	2.8 (21.7)	2.1 (25.6)	2.2 (27.1)
	HSS definition #2	1.3 (10.5)	0.5 (6.0)	0.9 (9.8)	0.8 (11.6)	0.8 (8.4)	0.7 (7.5)	0.6 (4.4)	0.6 (7.7)	0.6 (7.4)
	HSS definition #3	1.0 (9.0)	0.6 (7.0)	0.4 (3.9)	0.5 (6.9)	0.5 (6.0)	0.7 (7.3)	1.2 (9.8)	0.1 (2.1)	0.1 (1.2)
	Broader HSS definition	5.7 (50.0)	4.1 (51.5)	3.6 (38.9)	3.0 (41.0)	3.6 (42.2)	3.7 (37.5)	4.6 (35.9)	2.8 (35.4)	2.9 (35.7)
Hungary	PHC definition #1	NA	NA	NA	2.1 (39.0)	2.3 (32.5)	3.7 (49.6)	3.1 (48.9)	4.4 (32.3)	3.4 (26.3)

	PHC definition #2	NA	NA	NA	0.3 (6.2)	0.4 (5.5)	0.4 (6.2)	0.3 (4.2)	0.2 (2.1)	0.2 (1.7)
	PHC definition #3	NA	NA	NA	0.5 (10.0)	0.9 (13.0)	0.6 (6.7)	0.3 (5.5)	1.4 (10.0)	0.3 (2.4)
	PHC definition #4	NA	NA	NA	0.1 (2.2)	0.2 (2.4)	0.1 (1.4)	0.1 (0.9)	0.1 (0.7)	0.0 (0.4)
	Broader PHC definition	NA	NA	NA	3.0 (57.4)	3.8 (53.4)	4.8 (63.9)	3.8 (59.5)	6.1 (45.1)	3.9 (30.8)
	HSS definition #1	NA	NA	NA	1.1 (20.1)	1.3 (18.3)	1.5 (19.5)	1.3 (21.7)	1.9 (13.6)	2.7 (20.8)
	HSS definition #2	NA	NA	NA	0.4 (5.3)	0.4 (4.9)	0.5 (5.8)	0.3 (5.2)	1.5 (10.3)	4.1 (31.1)
	HSS definition #3	NA	NA	NA	0.9 (17.2)	1.7 (23.6)	0.8 (10.9)	0.8 (13.6)	4.2 (30.7)	2.2 (17.3)
	Broader HSS definition	NA	NA	NA	2.4 (42.6)	3.4 (46.8)	2.8 (36.2)	2.4 (40.5)	7.6 (54.6)	9.0 (69.2)
Iceland	PHC definition #1	1.6 (59.0)	2.2 (75.8)	3.5 (84.9)	2.7 (87.3)	1.6 (72.1)	2.1 (78.7)	0.9 (60.5)	1.2 (39.6)	2.8 (60.1)
	PHC definition #2	0.7 (25.8)	0.3 (10.4)	0.3 (7.0)	0.1 (4.2)	0.1 (6.4)	0.2 (7.2)	0.1 (9.0)	0.3 (9.8)	1.2 (27.3)
	PHC definition #3	0.1 (3.5)	0.0 (2.3)	0.1 (2.6)	0.1 (2.4)	0.2 (9.8)	0.1 (3.7)	0.1 (8.7)	0.1 (3.1)	0.1 (1.6)
	PHC definition #4	0.1 (2.9)	0.1 (1.8)	0.1 (1.6)	0.0 (1.1)	0.1 (2.4)	0.0 (1.7)	0.0 (2.2)	0.0 (0.9)	0.2 (3.4)
	Broader PHC definition	2.5 (91.2)	2.6 (90.3)	4.0 (96.1)	2.9 (95.0)	2.0 (90.7)	2.4 (91.3)	1.1 (80.4)	1.6 (53.4)	4.3 (92.4)
	HSS definition #1	0.1 (4.2)	0.1 (4.4)	0.1 (3.0)	0.1 (3.8)	0.1 (5.6)	0.2 (6.3)	0.2 (9.9)	0.2 (4.7)	0.1 (2.6)
	HSS definition #2	0.0 (1.4)	0.1 (3.2)	0.0 (0.8)	0.0 (1.0)	0.0 (2.1)	0.0 (1.9)	0.1 (4.0)	1.2 (39.4)	0.2 (4.0)
	HSS definition #3	0.1 (3.2)	0.1 (2.2)	0.0 (0.1)	0.0 (0.2)	0.0 (1.6)	0.0 (0.6)	0.1 (5.8)	0.1 (2.5)	0.0 (1.0)
	Broader HSS definition	0.2 (8.8)	0.3 (9.8)	0.1 (3.9)	0.1 (5.0)	0.1 (9.3)	0.2 (8.8)	0.4 (19.7)	1.5 (46.6)	0.3 (7.6)
Ireland	PHC definition #1	35.1 (28.0)	34.1 (28.4)	36.2 (27.7)	46.9 (36.6)	35.2 (32.8)	35.0 (32.4)	40.7 (37.6)	40.6 (41.6)	46.9 (44.0)
	PHC definition #2	4.5 (3.6)	3.3 (2.7)	3.6 (2.8)	2.8 (2.2)	10.3 (9.5)	3.6 (3.3)	3.7 (3.4)	3.1 (3.2)	4.2 (4.0)
	PHC definition #3	11.3 (8.9)	12.9 (10.7)	20.6 (15.8)	17.3 (13.6)	13.2 (12.4)	12.8 (11.7)	11.4 (10.4)	11.1 (11.2)	13.3 (12.6)
	PHC definition #4	31.8 (25.3)	28.6 (23.7)	27.3 (20.9)	21.6 (16.8)	13.9 (13.0)	15.8 (14.6)	13.6 (12.5)	14.2 (14.6)	12.5 (11.8)
	Broader PHC definition	82.7 (65.8)	78.9 (65.5)	87.7 (67.2)	88.6 (69.2)	72.6 (67.7)	67.2 (62.0)	69.4 (63.9)	69.0 (70.6)	76.9 (72.4)
	HSS definition #1	37.5 (29.9)	36.6 (30.4)	38.3 (29.3)	33.5 (26.1)	28.8 (26.9)	36.0 (33.3)	34.3 (31.6)	26.1 (26.7)	25.1 (23.6)
	HSS definition #2	2.9 (2.3)	2.0 (1.7)	1.7 (1.2)	3.1 (2.3)	3.3 (3.0)	2.7 (2.4)	2.6 (2.3)	1.6 (1.6)	2.3 (2.1)
	HSS definition #3	2.5 (2.0)	3.0 (2.5)	3.0 (2.2)	3.1 (2.4)	2.7 (2.6)	2.5 (2.2)	2.2 (2.1)	1.1 (1.1)	2.0 (1.9)
	Broader HSS definition	42.9 (34.2)	41.6 (34.6)	43.0 (32.7)	39.7 (30.8)	34.8 (32.5)	41.2 (37.9)	39.1 (36.0)	28.8 (29.4)	29.4 (27.6)
Italy	PHC definition #1	67.4 (39.4)	43.2 (32.9)	63.1 (39.7)	56.3 (28.8)	60.3 (27.6)	83.3 (34.7)	120.6 (43.6)	108.8 (36.7)	130.6 (40.0)
	PHC definition #2	7.6 (4.4)	7.9 (6.0)	10.7 (6.8)	9.1 (4.6)	9.7 (4.4)	11.0 (4.6)	10.3 (3.8)	14.6 (5.0)	13.8 (4.2)
	PHC definition #3	13.4 (7.8)	12.6 (9.5)	16.4 (10.3)	31.7 (16.2)	39.8 (18.2)	36.9 (15.5)	56.4 (20.5)	49.0 (16.5)	56.9 (17.4)
	PHC definition #4	15.7 (9.2)	5.6 (4.3)	11.8 (7.4)	30.2 (15.5)	20.0 (9.2)	26.2 (11.0)	2.5 (0.9)	19.9 (6.7)	23.3 (7.1)
	Broader PHC definition	104.1 (60.8)	69.3 (52.7)	102.0 (64.2)	127.3 (65.1)	129.8 (59.4)	157.4 (65.8)	189.8 (68.8)	192.3 (64.9)	224.6 (68.7)
	HSS definition #1	38.4 (22.5)	34.4 (26.2)	29.5 (18.6)	32.1 (16.4)	40.0 (18.4)	34.2 (14.3)	39.1 (14.2)	37.5 (12.7)	46.1 (14.1)
	HSS definition #2	19.2 (11.3)	22.4 (17.1)	22.5 (14.1)	29.1 (14.9)	41.1 (18.8)	36.4 (15.1)	25.2 (9.1)	59.6 (20.2)	50.0 (15.2)
	HSS definition #3	9.3 (5.4)	5.4 (4.2)	4.9 (3.0)	7.1 (3.6)	7.1 (3.3)	11.2 (4.6)	21.5 (7.8)	6.7 (2.3)	6.5 (2.0)
	Broader HSS definition	66.9 (39.2)	62.2 (47.5)	56.9 (35.7)	68.3 (34.9)	88.2 (40.5)	81.8 (34.0)	85.8 (31.1)	103.8 (35.2)	102.6 (31.3)
Japan	PHC definition #1	124.8 (17.9)	177.9 (20.9)	230.0 (32.1)	206.0 (24.1)	140.7 (16.3)	194.7 (21.9)	165.7 (15.2)	194.4 (20.5)	201.5 (19.6)
	PHC definition #2	61.0 (8.7)	55.6 (6.5)	54.8 (7.6)	59.6 (7.0)	60.0 (6.9)	72.6 (8.2)	58.6 (5.4)	68.1 (7.2)	52.5 (5.2)
	PHC definition #3	174.0 (25.0)	243.4 (28.5)	143.0 (20.0)	210.9 (24.7)	312.6 (36.1)	232.4 (26.2)	351.7 (32.1)	300.4 (31.7)	365.2 (35.7)
	PHC definition #4	95.4 (13.7)	171.3 (20.0)	84.8 (11.8)	173.2 (20.3)	113.0 (13.0)	87.0 (9.8)	125.6 (11.5)	102.5 (10.8)	116.3 (11.4)
	Broader PHC definition	455.2 (65.3)	648.2 (75.9)	512.6 (71.5)	649.7 (76.1)	626.3 (72.3)	586.7 (66.1)	701.6 (64.2)	665.4 (70.2)	735.5 (71.9)
	HSS definition #1	154.8 (22.2)	143.0 (16.7)	110.5 (15.4)	102.0 (11.9)	106.2 (12.3)	165.7 (18.6)	227.7 (20.8)	131.4 (13.9)	112.2 (11.0)
	HSS definition #2	76.8 (11.0)	34.0 (3.9)	65.4 (9.1)	76.0 (8.9)	103.9 (12.0)	123.3 (13.9)	160.7 (14.7)	131.2 (13.9)	167.6 (16.5)
	HSS definition #3	10.0 (1.4)	30.6 (3.6)	28.6 (4.0)	27.7 (3.3)	29.9 (3.4)	13.0 (1.4)	6.0 (0.5)	20.4 (2.2)	5.9 (0.6)
	Broader HSS definition	241.6 (34.6)	207.6 (24.2)	204.5 (28.5)	205.7 (24.1)	240.0 (27.7)	302.0 (33.9)	394.4 (36.0)	283.0 (30.0)	285.7 (28.1)
Luxembourg	PHC definition #1	20.8 (33.0)	23.3 (36.9)	27.0 (38.1)	25.1 (37.5)	13.0 (21.4)	8.3 (12.9)	11.0 (17.9)	12.7 (20.8)	17.8 (25.8)
	PHC definition #2	4.8 (7.7)	2.9 (4.7)	3.4 (4.7)	4.1 (6.1)	5.7 (9.4)	11.6 (18.3)	5.2 (8.4)	9.3 (14.9)	6.1 (9.0)
	PHC definition #3	6.1 (9.7)	4.5 (7.1)	4.3 (6.1)	4.5 (6.8)	6.1 (10.0)	3.9 (6.2)	4.8 (7.7)	3.9 (6.2)	6.9 (10.1)

	PHC definition #4	8.3 (13.2)	8.3 (13.2)	7.0 (9.8)	7.2 (10.8)	7.0 (11.5)	8.1 (12.8)	8.0 (13.1)	8.6 (14.0)	5.5 (8.1)
	Broader PHC definition	40.0 (63.6)	39.0 (61.9)	41.7 (58.7)	40.9 (61.2)	31.8 (52.3)	31.9 (50.2)	29.0 (47.1)	34.5 (55.9)	36.3 (53.0)
	HSS definition #1	13.7 (21.7)	11.5 (18.3)	16.6 (23.4)	10.7 (16.0)	13.6 (22.6)	18.7 (29.5)	20.9 (34.0)	17.8 (29.0)	25.5 (37.3)
	HSS definition #2	5.9 (9.4)	7.4 (11.9)	8.8 (12.3)	10.0 (15.0)	11.1 (18.4)	10.9 (17.2)	8.2 (13.4)	7.4 (11.9)	5.7 (8.4)
	HSS definition #3	3.3 (5.2)	5.1 (8.0)	4.0 (5.6)	5.2 (7.8)	4.1 (6.7)	2.0 (3.1)	3.5 (5.7)	1.9 (3.1)	0.9 (1.3)
	Broader HSS definition	22.9 (36.3)	24.0 (38.2)	29.4 (41.3)	25.9 (38.8)	28.8 (47.7)	31.6 (49.8)	32.6 (53.1)	27.1 (44.0)	32.1 (47.0)
Netherlands	PHC definition #1	146.6 (26.4)	104.4 (21.4)	103.6 (19.4)	93.7 (18.5)	109.2 (22.3)	130.6 (23.0)	117.5 (22.4)	145.6 (23.9)	113.2 (21.7)
	PHC definition #2	108.4 (19.5)	115.6 (23.7)	149.8 (28.0)	143.7 (28.5)	150.2 (30.7)	226.2 (39.7)	203.7 (38.9)	217.1 (35.5)	215.2 (41.1)
	PHC definition #3	63.4 (11.3)	36.0 (7.4)	57.3 (10.6)	55.5 (11.0)	59.6 (12.2)	52.5 (9.3)	56.0 (10.7)	57.7 (9.4)	72.2 (13.8)
	PHC definition #4	124.5 (22.4)	83.7 (17.2)	84.7 (15.8)	79.1 (15.7)	75.0 (15.3)	66.9 (11.7)	57.5 (11.0)	73.7 (12.0)	41.9 (8.0)
	Broader PHC definition	442.9 (79.6)	339.7 (69.7)	395.4 (73.8)	372.0 (73.7)	394.0 (80.5)	476.2 (83.7)	434.7 (83.0)	494.1 (80.8)	442.5 (84.6)
	HSS definition #1	54.8 (9.9)	60.7 (12.4)	42.9 (8.0)	53.7 (10.6)	51.0 (10.4)	28.9 (5.1)	22.3 (4.2)	42.5 (6.9)	31.5 (6.0)
	HSS definition #2	29.2 (5.2)	17.0 (3.4)	21.3 (3.9)	25.0 (5.0)	10.0 (2.0)	9.1 (1.6)	4.1 (0.7)	7.2 (1.2)	8.5 (1.7)
	HSS definition #3	29.4 (5.3)	70.8 (14.5)	75.4 (14.1)	53.6 (10.6)	34.0 (6.9)	54.9 (9.6)	63.1 (12.1)	67.5 (11.0)	41.3 (7.9)
	Broader HSS definition	113.4 (20.4)	148.5 (30.3)	139.6 (26.0)	132.3 (26.2)	95.0 (19.3)	92.9 (16.3)	89.5 (17.0)	117.2 (19.1)	81.3 (15.6)
New Zealand	PHC definition #1	10.0 (27.1)	9.9 (27.8)	8.8 (27.4)	7.6 (26.4)	7.6 (34.1)	4.6 (23.7)	4.8 (23.8)	7.6 (30.3)	7.8 (32.1)
	PHC definition #2	9.0 (24.7)	6.6 (18.5)	7.1 (21.8)	4.9 (17.1)	4.6 (21.2)	4.2 (21.6)	4.0 (19.6)	4.1 (16.2)	3.8 (15.5)
	PHC definition #3	3.6 (10.1)	2.1 (5.9)	2.0 (5.9)	2.4 (8.3)	0.8 (3.8)	0.8 (3.5)	4.0 (19.8)	3.1 (12.3)	5.5 (23.0)
	PHC definition #4	5.2 (14.3)	3.3 (9.3)	2.2 (6.6)	0.7 (2.4)	1.1 (4.9)	1.1 (5.7)	0.9 (4.4)	0.9 (3.7)	0.4 (1.5)
	Broader PHC definition	27.8 (76.2)	21.9 (61.5)	20.1 (61.7)	15.6 (54.2)	14.1 (64.0)	10.7 (54.5)	13.7 (67.6)	15.7 (62.5)	17.5 (72.1)
	HSS definition #1	4.6 (12.6)	6.5 (18.4)	3.6 (11.2)	1.7 (5.8)	1.0 (4.3)	1.2 (5.9)	1.3 (6.6)	2.2 (8.6)	1.2 (5.1)
	HSS definition #2	2.2 (6.0)	4.1 (11.6)	5.9 (17.9)	8.6 (29.9)	5.0 (22.7)	6.4 (32.6)	4.1 (19.8)	6.2 (24.4)	4.9 (20.5)
	HSS definition #3	1.8 (5.0)	3.0 (8.4)	3.0 (9.1)	2.9 (10.2)	2.0 (8.9)	1.3 (6.9)	1.2 (5.9)	1.1 (4.5)	0.5 (2.3)
	Broader HSS definition	8.6 (23.6)	13.6 (38.4)	12.5 (38.2)	13.2 (45.9)	8.0 (35.9)	8.9 (45.4)	6.6 (32.3)	9.5 (37.5)	6.6 (27.9)
Norway	PHC definition #1	92.2 (24.4)	120.1 (30.7)	125.6 (26.5)	140.5 (27.7)	160.2 (30.6)	229.1 (45.0)	246.6 (45.8)	217.1 (42.2)	207.2 (41.3)
	PHC definition #2	52.0 (13.7)	44.8 (11.5)	105.1 (22.2)	100.3 (19.9)	85.2 (16.3)	26.7 (5.3)	41.3 (7.7)	53.2 (10.4)	50.2 (9.9)
	PHC definition #3	56.2 (14.9)	40.8 (10.4)	42.5 (9.0)	71.8 (14.2)	91.1 (17.5)	88.2 (17.4)	107.2 (19.9)	111.1 (21.6)	115.0 (22.9)
	PHC definition #4	82.1 (21.7)	75.6 (19.3)	68.6 (14.5)	62.1 (12.3)	70.4 (13.5)	59.4 (11.7)	47.0 (8.7)	46.8 (9.1)	33.8 (6.7)
	Broader PHC definition	282.5 (74.7)	281.3 (71.9)	341.8 (72.2)	374.7 (74.1)	406.9 (77.9)	403.4 (79.4)	442.1 (82.1)	428.2 (83.3)	406.2 (80.8)
	HSS definition #1	41.4 (10.9)	51.0 (13.0)	54.2 (11.4)	56.2 (11.1)	53.9 (10.3)	53.6 (10.5)	48.6 (9.0)	43.1 (8.4)	63.1 (12.6)
	HSS definition #2	34.1 (9.0)	31.3 (8.0)	42.8 (9.0)	36.7 (7.2)	35.0 (6.7)	34.3 (6.7)	30.5 (5.7)	27.4 (5.3)	22.6 (4.4)
	HSS definition #3	21.1 (5.5)	27.6 (7.0)	36.0 (7.6)	38.8 (7.6)	26.5 (5.1)	17.1 (3.4)	17.6 (3.3)	16.3 (3.1)	10.3 (2.0)
	Broader HSS definition	96.6 (25.4)	109.9 (28.0)	133.0 (28.0)	131.7 (25.9)	115.4 (22.1)	105.0 (20.6)	96.7 (18.0)	86.8 (16.8)	96.0 (19.0)
Poland	PHC definition #1	NA	NA	7.4 (48.6)	6.4 (46.1)	7.1 (40.8)	12.9 (52.3)	13.8 (56.0)	9.5 (51.9)	12.9 (35.7)
	PHC definition #2	NA	NA	1.1 (7.0)	1.1 (7.9)	1.4 (7.8)	1.7 (6.7)	1.0 (4.4)	0.6 (3.3)	0.9 (2.4)
	PHC definition #3	NA	NA	1.6 (11.1)	1.1 (8.1)	2.1 (11.7)	1.7 (6.7)	1.0 (3.9)	1.2 (6.1)	1.6 (4.4)
	PHC definition #4	NA	NA	0.5 (3.2)	0.3 (2.1)	0.4 (2.5)	0.3 (1.3)	0.2 (0.7)	0.2 (1.0)	0.3 (0.7)
	Broader PHC definition	NA	NA	10.6 (69.9)	8.9 (64.2)	11.0 (62.8)	16.6 (67.0)	16.0 (65.0)	11.5 (62.3)	15.7 (43.2)
	HSS definition #1	NA	NA	2.8 (18.5)	2.9 (20.9)	4.1 (23.2)	4.8 (19.5)	4.9 (20.1)	4.9 (26.8)	6.0 (16.9)
	HSS definition #2	NA	NA	1.0 (6.7)	1.1 (7.6)	1.4 (7.9)	1.6 (6.6)	1.0 (3.7)	1.5 (8.5)	13.7 (38.9)
	HSS definition #3	NA	NA	0.7 (4.9)	1.0 (7.3)	1.1 (6.1)	1.6 (6.9)	2.7 (11.1)	0.5 (2.4)	0.3 (0.9)
	Broader HSS definition	NA	NA	4.5 (30.1)	5.0 (35.8)	6.6 (37.2)	8.0 (33.0)	8.6 (34.9)	6.9 (37.7)	20.0 (56.7)
Portugal	PHC definition #1	6.0 (22.6)	3.9 (19.7)	5.6 (21.7)	4.2 (18.4)	4.4 (14.0)	6.3 (27.7)	8.5 (43.5)	6.0 (36.2)	6.8 (29.8)
	PHC definition #2	1.0 (3.8)	0.6 (3.2)	0.9 (3.8)	0.8 (3.4)	0.9 (2.9)	1.2 (5.1)	1.2 (6.4)	1.3 (8.0)	1.1 (4.6)
	PHC definition #3	1.7 (6.5)	0.3 (1.9)	1.5 (5.7)	0.6 (2.3)	4.4 (13.7)	0.8 (3.1)	1.1 (5.7)	0.7 (4.2)	0.9 (4.0)
	PHC definition #4	1.6 (6.1)	0.3 (1.6)	0.3 (1.3)	0.1 (0.4)	0.2 (0.7)	0.3 (1.1)	0.3 (1.4)	0.3 (1.6)	0.2 (0.9)
	Broader PHC definition	10.3 (39.0)	5.1 (26.4)	8.3 (32.5)	5.7 (24.5)	9.9 (31.3)	8.6 (37.0)	11.1 (57.0)	8.3 (50.0)	9.0 (39.3)

	HSS definition #1	2.3 (8.4)	0.8 (4.3)	2.9 (11.4)	0.9 (3.8)	2.0 (6.2)	2.2 (9.5)	3.1 (15.9)	2.5 (15.0)	2.8 (12.2)
	HSS definition #2	13.0 (48.6)	12.8 (66.8)	13.7 (53.8)	16.2 (69.3)	19.6 (61.1)	11.4 (50.1)	3.6 (18.9)	5.5 (33.2)	10.9 (48.2)
	HSS definition #3	1.0 (3.9)	0.5 (2.5)	0.5 (2.2)	0.5 (2.4)	0.5 (1.5)	0.8 (3.5)	1.5 (8.1)	0.3 (1.7)	0.1 (0.6)
	Broader HSS definition	16.3 (60.9)	14.1 (73.6)	17.1 (67.4)	17.6 (75.5)	22.1 (68.8)	14.4 (63.1)	8.2 (42.9)	8.3 (49.9)	13.8 (61.0)
Slovak Republic	PHC definition #1	NA	NA	1.7 (51.2)	1.3 (43.2)	1.6 (41.5)	2.4 (56.2)	4.2 (65.1)	2.9 (59.8)	2.4 (50.3)
	PHC definition #2	NA	NA	0.2 (5.3)	0.3 (9.6)	0.2 (6.3)	0.2 (4.5)	0.2 (2.6)	0.3 (6.5)	0.2 (3.4)
	PHC definition #3	NA	NA	0.3 (9.1)	0.2 (7.7)	0.5 (14.3)	0.2 (5.1)	0.4 (7.0)	0.2 (5.3)	0.2 (5.6)
	PHC definition #4	NA	NA	0.0 (1.5)	0.0 (1.5)	0.1 (3.0)	0.0 (0.9)	0.0 (0.5)	0.0 (0.6)	0.0 (0.6)
	Broader PHC definition	NA	NA	2.2 (67.1)	1.8 (62.0)	2.4 (65.1)	2.8 (66.7)	4.8 (75.2)	3.4 (72.2)	2.8 (59.9)
	HSS definition #1	NA	NA	0.5 (15.7)	0.6 (19.3)	0.8 (21.2)	0.8 (17.3)	0.9 (14.0)	0.9 (18.0)	1.2 (25.6)
	HSS definition #2	NA	NA	0.4 (13.2)	0.3 (12.4)	0.3 (7.7)	0.4 (9.5)	0.2 (3.5)	0.4 (8.4)	0.7 (13.4)
	HSS definition #3	NA	NA	0.1 (4.2)	0.2 (6.4)	0.2 (5.9)	0.3 (6.5)	0.5 (7.5)	0.1 (1.3)	0.1 (1.2)
	Broader HSS definition	NA	NA	1.0 (33.1)	1.1 (38.1)	1.3 (34.8)	1.5 (33.3)	1.6 (25.0)	1.4 (27.7)	2.0 (40.2)
Slovenia	PHC definition #1	0.7 (37.7)	0.5 (20.5)	0.8 (31.3)	0.7 (44.6)	0.8 (39.8)	1.2 (48.8)	1.6 (51.0)	1.0 (50.4)	1.2 (51.2)
	PHC definition #2	0.1 (3.1)	0.1 (3.7)	0.1 (4.1)	0.1 (6.9)	0.2 (7.9)	0.2 (6.9)	0.1 (5.1)	0.1 (4.9)	0.1 (4.2)
	PHC definition #3	0.2 (9.8)	0.2 (7.4)	0.2 (9.1)	0.1 (8.4)	0.2 (11.6)	0.1 (6.0)	0.1 (5.0)	0.1 (6.4)	0.1 (5.2)
	PHC definition #4	0.1 (6.0)	0.1 (2.5)	0.1 (2.1)	0.0 (1.8)	0.0 (2.0)	0.0 (1.2)	0.0 (0.7)	0.0 (0.6)	0.0 (0.7)
	Broader PHC definition	1.1 (56.6)	0.9 (34.1)	1.2 (46.6)	0.9 (61.7)	1.2 (61.3)	1.5 (62.9)	1.8 (61.8)	1.2 (62.3)	1.4 (61.3)
	HSS definition #1	0.5 (20.3)	0.6 (20.5)	0.4 (14.3)	0.4 (21.7)	0.5 (21.9)	0.5 (19.1)	0.6 (19.9)	0.5 (23.7)	0.6 (24.0)
	HSS definition #2	0.4 (15.8)	1.4 (42.3)	0.9 (36.3)	0.1 (10.2)	0.2 (11.1)	0.3 (11.3)	0.2 (8.9)	0.2 (12.0)	0.3 (13.5)
	HSS definition #3	0.2 (7.3)	0.1 (3.1)	0.1 (2.8)	0.1 (6.5)	0.1 (5.9)	0.2 (6.4)	0.3 (9.5)	0.0 (1.9)	0.0 (1.1)
	Broader HSS definition	1.1 (43.4)	2.1 (65.9)	1.4 (53.4)	0.6 (38.4)	0.8 (38.9)	1.0 (36.8)	1.1 (38.3)	0.7 (37.6)	0.9 (38.6)
South Korea	PHC definition #1	69.2 (49.7)	93.5 (51.8)	90.4 (45.0)	76.9 (38.9)	99.4 (47.6)	117.6 (47.6)	83.5 (34.5)	81.2 (35.5)	74.7 (33.0)
	PHC definition #2	3.4 (2.4)	6.3 (3.5)	8.2 (4.1)	17.1 (8.7)	15.5 (7.3)	14.6 (5.9)	21.7 (9.0)	17.1 (7.5)	12.0 (5.2)
	PHC definition #3	14.0 (9.9)	15.5 (8.5)	16.7 (8.4)	13.7 (6.9)	18.4 (8.8)	15.4 (6.3)	21.1 (8.7)	24.1 (10.5)	25.1 (11.1)
	PHC definition #4	5.5 (4.0)	4.3 (2.4)	5.2 (2.6)	4.3 (2.2)	4.0 (1.9)	3.9 (1.6)	4.0 (1.6)	2.6 (1.1)	2.7 (1.2)
	Broader PHC definition	92.1 (66.0)	119.6 (66.2)	120.5 (60.1)	112.0 (56.7)	137.3 (65.6)	151.5 (61.4)	130.3 (53.8)	125.0 (54.6)	114.5 (50.5)
	HSS definition #1	14.4 (10.3)	17.6 (9.7)	21.5 (10.7)	21.7 (11.0)	31.2 (14.9)	28.9 (11.7)	31.4 (12.9)	28.5 (12.5)	25.3 (11.2)
	HSS definition #2	31.9 (22.8)	41.6 (23.0)	56.0 (28.0)	62.2 (31.4)	34.5 (16.5)	63.0 (25.6)	78.5 (32.4)	73.0 (32.0)	86.8 (38.2)
	HSS definition #3	1.1 (0.8)	1.8 (1.0)	2.5 (1.3)	1.5 (0.8)	6.5 (3.2)	3.4 (1.4)	2.0 (0.9)	2.0 (0.9)	0.4 (0.1)
	Broader HSS definition	47.4 (33.9)	61.0 (33.7)	80.0 (40.0)	85.4 (43.2)	72.2 (34.6)	95.3 (38.7)	111.9 (46.2)	103.5 (45.4)	112.5 (49.5)
Spain	PHC definition #1	78.2 (33.1)	40.3 (37.8)	40.3 (32.3)	37.7 (35.9)	33.7 (38.2)	61.3 (43.4)	68.1 (43.0)	58.0 (46.4)	55.9 (43.6)
	PHC definition #2	23.4 (9.9)	13.8 (12.8)	17.2 (13.7)	11.6 (11.2)	10.2 (11.6)	16.4 (11.6)	13.5 (8.5)	15.8 (12.6)	14.8 (11.6)
	PHC definition #3	19.5 (8.2)	9.7 (9.1)	9.5 (7.6)	9.6 (9.2)	6.7 (7.6)	8.5 (6.1)	17.7 (11.2)	7.8 (6.3)	9.0 (6.9)
	PHC definition #4	15.1 (6.4)	1.3 (1.3)	2.7 (2.2)	1.6 (1.6)	1.3 (1.5)	2.0 (1.4)	1.6 (1.0)	1.2 (0.9)	1.6 (1.2)
	Broader PHC definition	136.2 (57.6)	65.1 (61.0)	69.7 (55.8)	60.5 (57.9)	51.9 (58.9)	88.2 (62.5)	100.9 (63.7)	82.8 (66.2)	81.3 (63.3)
	HSS definition #1	70.2 (29.8)	23.8 (22.3)	32.7 (26.1)	25.3 (24.3)	24.1 (27.2)	28.6 (20.2)	33.3 (21.0)	22.0 (17.6)	26.7 (20.8)
	HSS definition #2	17.6 (7.5)	11.6 (10.9)	19.0 (15.1)	12.1 (11.5)	8.0 (9.1)	18.5 (13.0)	14.5 (9.1)	16.7 (13.4)	18.3 (14.4)
	HSS definition #3	11.8 (5.0)	6.1 (5.7)	4.0 (3.2)	6.5 (6.3)	4.3 (4.9)	6.2 (4.4)	9.9 (6.2)	3.5 (2.8)	1.9 (1.5)
	Broader HSS definition	99.6 (42.3)	41.5 (38.9)	55.7 (44.4)	43.9 (42.1)	36.4 (41.2)	53.3 (37.6)	57.7 (36.3)	42.2 (33.8)	46.9 (36.7)
Sweden	PHC definition #1	141.8 (32.4)	80.9 (17.3)	153.8 (32.2)	121.4 (28.1)	105.8 (23.0)	98.1 (21.1)	134.1 (26.3)	130.8 (25.6)	135.4 (29.3)
	PHC definition #2	55.0 (12.5)	98.3 (21.1)	67.8 (14.2)	76.8 (17.8)	85.7 (18.7)	112.9 (24.3)	128.1 (25.2)	121.3 (23.8)	125.9 (27.2)
	PHC definition #3	46.8 (10.7)	57.8 (12.4)	58.0 (12.0)	47.8 (11.0)	82.5 (17.9)	68.9 (14.8)	72.6 (14.2)	74.7 (14.6)	79.2 (17.1)
	PHC definition #4	122.4 (27.9)	145.5 (31.2)	117.9 (24.6)	81.7 (18.9)	93.3 (20.3)	94.8 (20.4)	73.0 (14.3)	75.6 (14.8)	44.8 (9.7)
	Broader PHC definition	366.0 (83.5)	382.5 (82.0)	397.5 (83.0)	327.7 (75.8)	367.3 (79.9)	374.7 (80.6)	407.8 (80.0)	402.4 (78.8)	385.3 (83.3)
	HSS definition #1	20.6 (4.7)	23.7 (5.1)	18.9 (3.9)	27.1 (6.3)	24.6 (5.4)	45.1 (9.7)	41.9 (8.2)	45.3 (8.9)	35.1 (7.6)
	HSS definition #2	27.9 (6.4)	23.6 (5.1)	26.8 (5.6)	35.0 (8.1)	39.4 (8.7)	22.3 (4.8)	38.5 (7.5)	41.6 (8.2)	31.7 (6.9)

	HSS definition #3	24.0 (5.5)	36.5 (7.8)	35.6 (7.4)	42.8 (9.9)	27.7 (6.1)	22.6 (4.9)	21.0 (4.1)	20.9 (4.1)	11.2 (2.4)
	Broader HSS definition	72.5 (16.6)	83.8 (18.0)	81.3 (16.9)	104.9 (24.3)	91.7 (20.2)	90.0 (19.4)	101.4 (19.8)	107.8 (21.2)	78.0 (16.9)
Switzerland	PHC definition #1	45.4 (31.9)	46.4 (32.2)	48.5 (32.9)	50.2 (24.5)	53.0 (26.7)	56.5 (30.5)	63.7 (29.8)	69.7 (34.3)	69.5 (34.4)
	PHC definition #2	20.8 (14.6)	19.0 (13.1)	14.7 (9.9)	16.8 (8.2)	15.2 (7.6)	16.7 (9.1)	17.3 (8.1)	16.7 (8.2)	16.1 (8.0)
	PHC definition #3	15.6 (11.0)	15.4 (10.6)	20.0 (13.6)	43.8 (21.3)	39.2 (19.8)	26.2 (14.2)	33.4 (15.6)	30.8 (15.2)	34.4 (17.0)
	PHC definition #4	20.8 (14.6)	19.4 (13.4)	20.0 (13.5)	20.5 (10.0)	27.2 (13.7)	28.0 (15.1)	25.4 (11.9)	22.8 (11.2)	14.6 (7.3)
	Broader PHC definition	102.6 (72.1)	100.2 (69.3)	103.2 (69.9)	131.3 (64.0)	134.6 (67.8)	127.4 (68.9)	139.8 (65.4)	140.0 (68.9)	134.6 (66.7)
	HSS definition #1	30.8 (21.6)	25.5 (17.7)	36.7 (24.9)	49.8 (24.3)	46.6 (23.5)	40.6 (21.9)	51.5 (24.0)	46.5 (22.9)	45.8 (22.7)
	HSS definition #2	3.4 (2.4)	2.8 (1.9)	4.8 (3.2)	13.2 (6.4)	8.7 (4.4)	11.3 (6.1)	17.4 (8.1)	11.9 (5.8)	18.3 (9.1)
	HSS definition #3	5.8 (4.0)	16.1 (11.1)	2.9 (1.9)	11.2 (5.4)	8.7 (4.4)	5.8 (3.1)	5.5 (2.6)	4.7 (2.3)	2.6 (1.4)
	Broader HSS definition	40.0 (28.0)	44.4 (30.7)	44.4 (30.0)	74.2 (36.1)	64.0 (32.3)	57.7 (31.1)	74.4 (34.7)	63.1 (31.0)	66.7 (33.2)
United Kingdom	PHC definition #1	409.0 (19.9)	617.1 (28.3)	930.0 (28.3)	876.4 (31.1)	737.1 (33.1)	722.7 (32.9)	815.6 (29.1)	717.6 (24.9)	674.6 (23.5)
	PHC definition #2	325.2 (15.8)	453.4 (20.8)	384.6 (11.7)	616.3 (21.9)	490.5 (22.1)	454.2 (20.7)	459.4 (16.3)	479.5 (16.7)	535.0 (18.7)
	PHC definition #3	532.8 (25.9)	430.8 (19.7)	1024.9 (31.3)	656.9 (23.3)	499.2 (22.5)	462.1 (21.0)	577.7 (20.6)	592.3 (20.6)	652.3 (22.8)
	PHC definition #4	469.6 (22.8)	298.3 (13.7)	516.4 (15.8)	290.6 (10.3)	122.2 (5.5)	135.0 (6.2)	192.3 (6.9)	194.0 (6.7)	164.3 (5.7)
	Broader PHC definition	1736.6 (84.4)	1799.6 (82.5)	2855.9 (87.1)	2440.2 (86.6)	1849.0 (83.2)	1774.0 (80.8)	2045.0 (72.9)	1983.4 (68.9)	2026.2 (70.7)
	HSS definition #1	168.7 (8.2)	208.8 (9.6)	260.9 (8.0)	228.3 (8.1)	201.1 (9.0)	215.4 (9.8)	295.1 (10.5)	319.3 (11.1)	274.5 (9.6)
	HSS definition #2	114.8 (5.6)	132.7 (6.1)	119.4 (3.7)	103.7 (3.7)	135.1 (6.1)	173.4 (7.9)	403.0 (14.4)	529.6 (18.5)	526.8 (18.3)
	HSS definition #3	36.2 (1.8)	41.9 (2.0)	39.8 (1.2)	47.9 (1.7)	38.9 (1.8)	31.8 (1.5)	64.0 (2.3)	42.0 (1.4)	35.1 (1.3)
	Broader HSS definition	319.7 (15.6)	383.4 (17.7)	420.1 (12.9)	379.9 (13.5)	375.1 (16.9)	420.6 (19.2)	762.1 (27.2)	890.9 (31.0)	836.4 (29.2)
United States	PHC definition #1	478.5 (4.8)	640.8 (6.6)	763.3 (7.5)	636.6 (6.1)	761.6 (8.3)	734.1 (6.5)	877.8 (7.9)	769.3 (7.5)	795.2 (9.9)
	PHC definition #2	1118.1 (11.3)	1133.2 (11.4)	1214.9 (11.8)	1164.1 (11.1)	1401.3 (15.3)	1147.9 (10.3)	1221.7 (11.1)	1292.6 (12.6)	996.5 (12.4)
	PHC definition #3	1541.6 (15.6)	1666.6 (16.9)	1709.0 (16.7)	2151.2 (20.6)	1911.5 (20.8)	2659.5 (24.0)	2271.0 (20.6)	1768.8 (17.2)	2026.4 (25.2)
	PHC definition #4	6365.5 (64.3)	6196.9 (62.7)	6293.0 (61.5)	6151.2 (58.8)	4837.4 (52.8)	6011.3 (54.1)	6493.4 (58.8)	5893.4 (57.4)	3945.2 (49.0)
	Broader PHC definition	9503.7 (96.0)	9637.5 (97.6)	9980.2 (97.5)	10103.1 (96.6)	8911.8 (97.2)	10552.8 (94.9)	10863.9 (98.4)	9724.1 (94.7)	7763.3 (96.5)
	HSS definition #1	326.2 (3.3)	185.6 (1.9)	182.4 (1.8)	289.9 (2.8)	179.6 (2.0)	488.2 (4.4)	109.2 (1.0)	471.2 (4.6)	213.7 (2.7)
	HSS definition #2	26.3 (0.3)	23.0 (0.3)	19.8 (0.2)	24.6 (0.2)	26.0 (0.3)	17.6 (0.1)	25.9 (0.2)	25.0 (0.2)	30.6 (0.3)
	HSS definition #3	50.7 (0.5)	43.8 (0.4)	42.8 (0.4)	45.7 (0.4)	49.8 (0.5)	48.2 (0.4)	36.2 (0.3)	41.2 (0.4)	41.9 (0.5)
	Broader HSS definition	403.2 (4.1)	252.4 (2.6)	245.0 (2.4)	360.2 (3.4)	255.4 (2.8)	554.0 (4.9)	171.3 (1.5)	537.4 (5.2)	286.2 (3.5)
All members	PHC definition #1	2621.0 (14.1)	2950.4 (15.7)	3655.0 (18.0)	3329.0 (16.3)	3211.4 (17.6)	3590.0 (17.6)	3893.4 (18.2)	3611.8 (17.4)	3690.3 (20.0)
	PHC definition #2	2078.5 (11.2)	2240.2 (12.0)	2366.8 (11.6)	2559.6 (12.6)	2653.7 (14.6)	2417.1 (11.9)	2560.3 (11.9)	2696.2 (13.0)	2426.9 (13.1)
	PHC definition #3	3213.1 (17.2)	3274.6 (17.5)	3905.3 (19.2)	4054.3 (20.0)	4063.1 (22.3)	4657.0 (22.8)	4601.7 (21.5)	4190.1 (20.2)	4566.0 (24.6)
	PHC definition #4	8236.1 (44.3)	7876.5 (42.2)	7946.2 (39.1)	7566.4 (37.2)	6023.1 (33.1)	7041.7 (34.5)	7477.5 (34.9)	6873.6 (33.1)	4776.3 (25.8)
	Broader PHC definition	16148.7 (86.8)	16341.7 (87.4)	17873.3 (87.9)	17509.3 (86.1)	15951.3 (87.6)	17705.8 (86.8)	18532.9 (86.5)	17371.7 (83.7)	15459.5 (83.5)
	HSS definition #1	1536.3 (8.3)	1331.7 (7.1)	1359.5 (6.7)	1606.2 (7.9)	1176.4 (6.5)	1584.0 (7.8)	1428.3 (6.7)	1726.8 (8.3)	1436.6 (7.8)
	HSS definition #2	597.4 (3.2)	559.6 (3.0)	645.6 (3.2)	685.1 (3.4)	676.2 (3.7)	773.1 (3.8)	1024.9 (4.8)	1239.0 (5.9)	1318.7 (6.9)
	HSS definition #3	325.1 (1.7)	451.5 (2.4)	437.2 (2.2)	531.2 (2.7)	396.0 (2.2)	362.4 (1.8)	461.7 (2.1)	431.8 (2.1)	320.4 (1.8)
	Broader HSS definition	2458.8 (13.2)	2342.8 (12.5)	2442.3 (12.1)	2822.5 (14.0)	2248.6 (12.4)	2719.5 (13.4)	2914.9 (13.6)	3397.6 (16.3)	3075.7 (16.5)

DAH: development assistance for health; DAH: Development Assistance Committee; PHC: public health care; HSS: health system strengthening. PHC definition #1 = Basic health care and infrastructure (CRS purpose codes: 12220, 12230, 12240, 12261, 12281); PHC definition #2 = Reproductive health care and family planning (13020, 13030); PHC definition #3 = Infectious disease control, including malaria and tuberculosis (12250, 12262, 12263); PHC definition #4 = Sexually transmitted disease (STD) control including HIV/AIDS (13040); Broader PHC definition = Definition #1 + #2 + #3 + #4; HSS definition #1 = Health policy, administration & management (12110); HSS definition #2 = Medical

services, training & research (12181, 12182, 12191, 12310, 12320, 12330, 12340, 12350, 12382); HSS definition #3 = Population policy & administration (13010, 13081); Broader HSS definition = Definition #1 + #2 + #3.
